# Fluorescence Lifetime Multiplexing with Fluorogen-Activating FAST Protein Variants and Red-Shifted Arylidene–Imidazolone Derivative as Fluorogen

**DOI:** 10.3390/bios15050274

**Published:** 2025-04-29

**Authors:** Aidar R. Gilvanov, Ivan N. Myasnyanko, Sergey A. Goncharuk, Marina V. Goncharuk, Vadim S. Kublitski, Daria V. Bodunova, Svetlana V. Sidorenko, Eugene G. Maksimov, Mikhail S. Baranov, Yulia A. Bogdanova

**Affiliations:** 1 Institute of Bioorganic Chemistry, Russian Academy of Sciences, Miklukho-Maklaya 16/10, Moscow 117997, Russia; 2 Laboratory of Medicinal Substances Chemistry, Institute of Translational Medicine, Pirogov Russian National Research Medical University, Ostrovitianov 1, Moscow 117997, Russia; 3 Department of Biology, Lomonosov Moscow State University, Leninskye Gory, Build. 12, Moscow 119234, Russia

**Keywords:** arylidene–imidazolone, FLIM, fluorogen-activating protein

## Abstract

Fluorescence-lifetime imaging microscopy (FLIM) is a powerful technique for highly multiplexed imaging in live cells. In this work, we present a genetically encoded FLIM multiplexing platform based on a combination of fluorogen-activating protein FAST and red-shifted fluorogen **N871b** from the arylidene–imidazolone family. We showed that a series of FAST protein mutants exhibit similar steady-state optical properties in complex with **N871b** fluorogen but have different fluorescence lifetimes. The similar brightness and binding strength of pairs of these FAST protein variants with **N871b** allows them to be successfully used for multiplexing up to three intracellular structures of living cells simultaneously.

## 1. Introduction

Modern cell biology often demands real-time, simultaneous observation of multiple biological processes or compartments within a single specimen, which implies the use of multiplex systems. Fluorescence-lifetime imaging microscopy (FLIM) [1] is an attractive platform for the creation of such systems. Unlike conventional multicolor fluorescence microscopy, FLIM relies on the fluorescence lifetime of a fluorophore, which may serve as a unique identifier for specific fluorophore. This enables simultaneous detection of several spectrally similar tags using a single excitation source, preserving other spectral channels for further multiplexing. 

**Figure 1:**
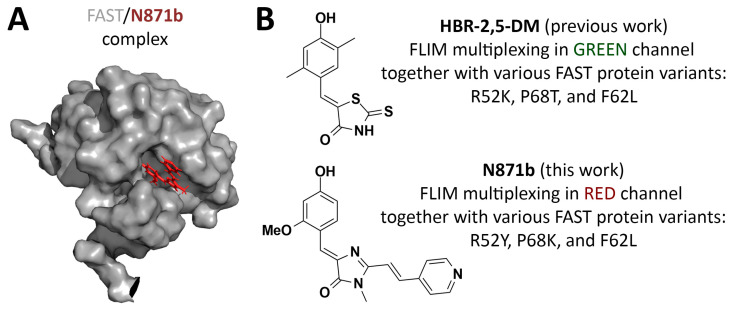
Fluorogen-activating protein FAST and its fluorogens in FLIM multiplexing. (**A**) Structure of the FAST/**N871b** complex. (**B**) Fluorogens used for FLIM multiplexing alongside FAST protein variants proposed previously and in this work.

Heavily multiplexed FLIM-based systems were reported for synthetic fluorescent probes [2], fluorescent proteins [3], and self-labeling protein tags (SLPs) such as HaloTag [4]. Synthetic fluorescent probes may be an attractive approach due to the seeming simplicity and availability of various probes. However, labeling multiple intracellular targets with such probes may require several different protocols [5]. Additionally, nonspecific labeling may lead to a generation of multiple lifetime populations, complicating FLIM multiplexing assays [2]. Instead, high labeling specificity can be achieved with genetically encodable or chemogentic tags fused or bioconjugated to proteins of interest or localization signals [6,7]. Despite the abundance of fluorescent proteins with different spectral properties, their fluorescence lifetime window is quite narrow (2.3–3.5 ns) [8], with only a few known exceptions [9,10,11]. This limitation can be addressed using SLPs with various external fluorophores. However, like fluorescent proteins, SLPs are also prone to photobleaching due to the covalent binding of fluorophores. Moreover, the large molecular weights of fluorescent proteins (27 kDa) and HaloTag (33 kDa) can hinder the co-expression and functionality of fused proteins of interest [12,13,14]. Given the limitations of the aforementioned tags, fluorogen-activating proteins represent a promising alternative [15]. These chemogenetic tags lack intrinsic chromophores and depend on external fluorogens that are weakly fluorescent in solution but become highly emissive upon forming non-covalent complexes with the protein (Figure 1). The non-covalent nature of the complex enables rapid exchange of fluorogen between the solution and protein pocket, thereby enhancing the photostability of the tag. One of the well-established fluorogen-activating proteins is the fluorescence-activating and absorption-shifting tag (FAST) [16], with a molecular weight of only 14 kDa. Its small size and absence of chromophore maturation step make FAST an attractive fluorescent reporter for both eukaryotic cells [16,17] and anaerobic bacteria [18,19]. Furthermore, circularly permuted and split variants of FAST were reported, potentially broadening the range of biosensing platforms and protein–protein interaction studies [20,21].

FAST-based labeling has previously been used in various microscopy applications, including super-resolution imaging and FLIM [22,23,24,25,26]. Recently, our group [27] and Gautier’s [28] team described systems for multiplex FLIM based on different FAST variants and derivatives of several arylidene-rhodanines and one arylidene-imidazolone as fluorogens. These systems demonstrated reliable signal separation of triplicates in spatially distinct intracellular compartments within live cells for both fit-based and non-fitting phasor-based data analysis. However, the fluorescent labeling system we proposed was characterized by emission in the green and yellow spectral channels (emission maxima in the 525–575 nm area). Here we broaden the spectral palette using the previously proposed [22] red-shifted (emission maxima in the 600–650 nm area) arylidene-imidazolone-based fluorogen **N871b** (Figure 1, Supplementary Figure S1). In comparison to arylidene-rhodanines, arylidene-imidazolones provide a vast diversity for structural modification and thereby spectral properties [22,26,29]. **N871b** has been shown to possess a lower quantum yield in unbound form at physiological pH, while its complex with FAST exhibits higher photostability in comparison to two arylidene-rhodanine fluorogens. Importantly, **N871b** is non-toxic in concentrations up to 10 μM and does not hinder cell division [22]. We investigated the spatial structure of the FAST protein with **N871b** [29] (Figure 1), as a result, a series of the FAST point mutants (variants), optimized for complex formation with fluorogens, were obtained through rational design. In the present study, we employed mentioned variants of the FAST protein and fluorogen **N871b** as the basis for the red-shifted FLIM multiplexing system in live cells. As in our previous work [27], we utilized the multiexponential fitting of fluorescence decay cross-verified with phasor plot analysis.

## 2. Materials and Methods

### 2.1. Synthesis

The synthesis of compound **N871b** was described previously [22]. For this work, compound **N871b** was taken from our laboratory stock. Description of NMR and HRMS data as well as freshly registered NMR spectra of this compound are provided in SI.

### 2.2. Plasmids

A pET24b(+) vectors coding FAST variants with C-terminal his-tag (GGGHHHHHH) were used for in vitro screening in *E. coli* and were obtained from a commercial source (Cloning Facility, Russia).

The assembly of plasmids for expression in HeLa Kyoto cells was carried out with Golden Gate assembly following MoClo syntax [30]. The Eco31I (BsaI) restriction endonuclease (Thermo Scientific, Waltham, MA, USA) and T4 DNA Ligase (Evrogen, Moscow, Russia) were used for the cloning procedure. Sequences coding FAST variants (D65K, F62L, P68K, or R52Y) and sequences coding localization signals (H2B, IMS or vimentin) were put under CMV promoter and SV40 poly(A) sequence. Sequences of FAST variants in Level 0 plasmids were obtained from a commercial source (Cloning Facility, Moscow, Russia). The Level 0 plasmids coding CMV promoter, SV40 poly(A) and localization signals were available in-house. 

### 2.3. Primary Fluorescence Lifetime Screening In Vitro with Edinburgh Instruments Mini-Tau Spectrometer

The plasmids coding FAST variants were transformed into chemically competent BL21(DE3) *E. coli* cells with heat-shock transformation followed by plating onto Petri dishes with LB-agar medium supplemented with 100 μg/mL of ampicillin and incubated at 37 °C overnight. Then a single colony of each variant was resuspended in 5 mL of sterile LB medium with 100 μg/mL of ampicillin and incubated in an orbital shaker at 37 °C and 220 rpm for 16 h.

Bacterial cells were pelleted at 1700 g and then resuspended in 2 mL of sterile PBS buffer (pH 7.4). Resuspended biomass was ultrasonicated with a VCX500 Ultrasonic processor, equipped with a 2 mm tapered microtip (Sonics&Material Inc., Newtown, CT, USA). The ultrasonication was carried out for 20 min in pulse mode with an active interval of 5 sec at 40% amplitude followed by cooling for 15 sec.

The lysates were clarified by centrifugation at 14,000× *g* for 30 min at 4 °C; 900 ul of supernatant was mixed with **N871b** fluorogen at the final concentration of 1 uM in 4.2 mL PMMA transparent optical cuvettes (Sarstedt, Hildesheim, Germany). **N871b** was diluted from 10 mM stocks in DMSO.

Fluorescence of complexes of FAST variants with **N871b** was verified on a Cary Eclipse fluorescence spectrometer (Agilent Technologies, Santa Clara, CA, USA) by recording emission spectra in a 590–700 nm window with excitation at 540 nm and was compared to data on a mixture of **N871b** with lysate of BL21(DE3) transformed by empty pcDNA3.1(+) plasmid vector to eliminate false-positive results. Verified mixtures were used for lifetime data acquisition on a time-resolved mini-Tau fluorescence spectrometer (Edinburgh Instruments, Livingston, UK). The EPLED-590 picosecond laser (Edinburgh Instruments, Livingston, UK) was used for fluorescence excitation. The central emission wavelength was set to 589.1 nm with repetition rate of 20 Mhz, and the emission filter with a spectral range of 575–625 was used. The photon count was carried out within a 50 ns window divided into 1024 channels. Fluoracle 2.5.1. software (Edinburgh Instruments, Livingston, UK) was used for lifetime data processing and determination of χ2 (Pearson’s test). Deconvolution with the instrument response function (IRF-based fit) was carried out. The resulting decay graphs were analyzed with Origin 2021 software suite (OriginLab Corporation, Northampton, MA, USA) (see Supplementary Figures S2–S18).

### 2.4. Fluorescence-Lifetime Imaging Microscopy of HeLa Kyoto Live Cells

HeLa Kyoto cells were obtained from our laboratory stock. Cells were seeded onto 35 mm glass-bottomed culture dishes (SPL Life Sciences, Pocheon, Korea) and grown in DMEM medium (PanEco, Moscow, Russia) containing 10% (*v*/*v*) of fetal bovine serum (Sigma, St. Louis, MO, USA), 50 U/mL of penicillin, and 50 μg/mL of streptomycin (PanEco, Moscow, Russia) (DMEM complete) at 37 °C in an atmosphere with 5% CO_2_.

Transient transfection with plasmids containing FAST fusions was performed using polyethilenimine (PEI) (Polysciences, Warrington, PA, USA). The PEI:DNA ratio was 4:1. DMEM complete was changed for Opti-MEM medium 1 h before the transfection procedure, and cells were incubated with transfection mix for 4 h following returning cells to DMEM complete. 

FLIM of live HeLa Kyoto cells was performed in 2 mL of Hanks’ Balanced Salt Solution (PanEco, Moscow, Russia) containing 10 mM HEPES (Sigma, St. Louis, MO, USA) and 5 µM of **N871b** (added from 10 mM stock solution in DMSO) at room temperature, using an Eclipse Ti2 (Nikon, Tokyo, Japan) microscope with a Nikon 60× 1.4 oil immersion lens (Nikon, Tokyo, Japan), equipped with the DCS-120 scanning confocal module, HMP-100-40C detector, and SPC-150 module (Becker&Hickl, Berlin, Germany). For fluorescence excitation, a 473 nm picosecond laser BDS-SM-LS-101 (Becker&Hickl, Berlin, Germany) with 30 ps duration pulses exciting samples at a repetition rate of 50 MHz was used. The average input laser power was set to 5–7% of maximum, and collection time was set to 120 s. The detection was performed using a combination of 485 nm long-pass filter and a bandpass HQ590/40 filter (Chroma, USA).

### 2.5. Fluorescence-Lifetime Imaging Microscopy Processing

The control of the Becker&Hickl system mentioned above and data acquisition were carried out with SPCM data acquisition software SPC-150 v.9.87 (Becker&Hickl, Berlin, Germany). The obtained data in .sdt format was analyzed via SPCImage software 8.9.

The fluorescence decay curve is described by the following equation:

(1) $$F\left(t\right)=\sum_{k=1}^{n}A_{k}e^{-t/\tau_{k}}$$

where F(t) is the fluorescence intensity as a function of time, A_k_ is the amplitude of the k-th decay component, and τ_k_ is the fluorescence lifetime of the k-th component.

Amplitude-weighted average lifetime can be described by the following equation:

(2) $$\tau_{m}=\frac{\sum_{k=1}^{n}A_{k}\tau_{k}}{\sum_{k=1}^{n}A_{k}}$$

The amplitude-weighted average lifetime weights each lifetime component (τ_k_) by their amplitude coefficient (A_k_).

The intensity-weighted average lifetime can be described by the following equation:

(3) $$\tau_{i}=\frac{\sum_{k=1}^{n}A_{k}\tau_{k}^{2}}{\sum_{k=1}^{n}A_{k}\tau_{k}}$$

The intensity-weighted average lifetime weights each k-th lifetime component (τ_k_) by its intensity (A_k_τ_k_).

The spatial binning, which allows aggregation of photons from neighboring pixels, was used for the improvement of fitting quality.

For each FAST protein variant, a comparative analysis of the fitting quality provided by exponential models of different complexity (single-, two-, and three-component models) was carried out. A fitting was considered reliable when the χ^2^ value was equal to or less than 1.2. The simpler fitting model was preferred, when exponential models of different complexity provided fits of comparable quality.

Specific protocols for data analysis and image processing are provided below.

Data processing for Figure 2, panel A of Figure 3, panel B of Figure 4, Supplementary Figures S19–S60, Table 1, Supplementary Tables S3 and S4.

1. The blue crosshair was moved to the uniformly fluorescent region of the cell with medium signal intensity.
2. The spatial binning factor (n) was set to 3–6 (“Bin” in the “Decay-Graph” window). The threshold parameter was set to 100–600. The time channel range used for fitting was adjusted with T1 and T2 values so the whole decay curve was included without the baseline regions. The number of exponential components was set to 2 or 3 in a “Multiexponential Decay” window.
3. The decay matrix was calculated using the corresponding command (Calculate > Decay matrix).
4. In each experiment, the lifetime data was collected from random cells in 4–6 fields of view.
5. The color-coded and total intensity images were exported in .tiff format (File > Export…, “Color-coded image”, and “Gray-scale image”).
6. Fitting results were analyzed using the Origin 2021 Software suite, and micrographs were processed with Fiji 1.54k.

Data processing for panel B of Figure 3.

1. The blue crosshair was moved to the uniformly fluorescent region of the cell with medium signal intensity.
2. The binning factor (n) was set to 3 (“Bin” in the “Decay-Graph” window). The threshold parameter was set to 100. The time channel range used for fitting was adjusted with T1 and T2 values to include the whole decay curve without the baseline regions. The number of exponential components was set to 2 in a “Multiexponential Decay” window.
3. The color-coding was switched from continuous to discrete (Options > Color, “Mode discrete”), and the range of τ_i_ values was assigned to red, green, or blue color channels.
4. The resulting FLIM images were exported as .tiff files and imported to FIJI 1.54k as RGB color-type files.
5. RGB files were transformed to RGB stacks (Image > Type > RGB stack). The stacks were separated into three images (Image > Stacks > Stack to Images), one of which was empty. The resulting images contained pixels with a τ_i_ from an assigned range.
6. Each image was transformed into 16-bit files (Image > Type > 16 bit); a colorblind-friendly palette was used for final figures representation (Image > LUT).

Data processing for panel C of Figure 3.

1. Steps 1–2 are identical to those in the protocol for panel b.
2. Phasor plot was calculated (“Phasor plot” button).
3. The color-coding was switched from continuous to discrete mode (Options > Color, “Mode discrete”) and the range of red color was set from 1 to 2000 ps; green and blue channels were left empty.
4. The box “Select cluster” was marked and the cluster for vimentin was selected.
5. The image was exported in .tiff format.
6. The range of the green channel was set from 1 to 2000 ps, and the red and blue channels were left empty.
7. Steps 4–5 were repeated for the H2B cluster.
8. FIJI 1.54k was used to prepare figures for publication.

Data processing for panels D-F of Figure 4.

**Figure 2:**
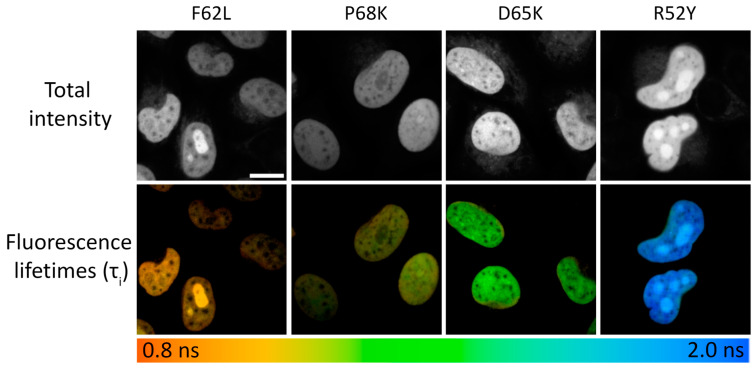
Live-cell FLIM microscopy of **N871b** fluorogen complexes with FAST variants F62L, P68K, D65K, and R52Y expressed as fuses with histone H2B. The upper panel represents total intensity. The lower panel represents color-coded fluorescence lifetime as a result of the bi-exponential fit, and is shown as τ_i_ (for all images n = 30 individual cells). Scale bar—20 μm.

1. Steps 1–2 are identical to those in the protocol for panel c of Figure 2.
2. The color-coding was switched from continuous to discrete mode (Options > Color, “Mode discrete”) and the ranges of the blue and green colors were set from 1 to 2000 ps, the red channel was left empty.
3. The box “Select cluster” was marked, and the cluster for nuclei was selected.
4. The image was exported in .tiff format.
5. The color-coding was set to discrete mode (Options > Color, “Mode discrete”) and the ranges of the red and green colors were set from 1 to 2000 ps, the Blue channel was left empty.
6. Steps 3–4 were repeated for the mitochondria cluster.
7. The range of the green channel was set from 1 to 2000 ps, and the red and blue channels were left empty.
8. Steps 4–5 were repeated for vimentin cluster.
9. FIJI 1.54k was used to prepare figures for publication.

## 3. Results and Discussion

### 3.1. Primary Fluorescence-Lifetime Screening In Vitro

Based on previous data [29] (Supplementary Tables S1 and S2), we selected 17 FAST variants that form complexes with **N871b** with high quantum yields and low K_d_ values. These proteins were expressed in *E. coli*, and bacterial lysates were used for in vitro screening based on time-correlated single photon counting (TCSPC) spectroscopy in the presence of **N871b** (see Section 2 for more details). In all probes, we observed bi-exponential fluorescence decay (Supplementary Figures S2–S18). We chose four FAST variants for further examination: one variant with the longest fluorescence intensity-weighted average lifetime τ_i_ (R52Y), two variants with short τ_i_ (F62L and P68K), and one variant with an intermediate τ_i_ (D65K) (Table 1, Supplementary Table S2). 

### 3.2. Fluorescence-Lifetime Measurements in Cells

We tested selected FAST variants in FLIM in live HeLa Kyoto cells. For this purpose, we expressed selected variants as fusions with the histone protein H2B [31] to selectively tag nuclei and obtain bright and uniform fluorescence signals (Figure 2). It is worth noting that we did not observe any changes in cell morpholgy during prolonged FLIM experiments.

At spatial binning values of 3–6, the decay data typically had 1000 photons at the peak, allowing the use of multicomponent fitting models. In all cases, adequate fitting was achieved using the bi-exponential model without further significant enhancement of the χ^2^ value with the use of the tri-exponential model (Supplementary Table S3). Generally, the lifetimes obtained *in cellulo* were significantly shorter than those obtained in vitro. This discrepancy may be explained by the increased representativity of short-living fluorescence species, probably due to quenching of complex fluorescence by a dense packing of molecules in live cells or the presence of natural quenchers (Table 1, Figure 2, Supplementary Tables S2 and S3, Supplementary Figures S19–S28 and S45).

To explore further multiplexing *in cellulo*, we analyzed two additional intracellular localizations tagged with FAST variants: the mitochondrial intermembrane space (IMS) [32] and the intermediate filament protein vimentin (Table 1, Supplementary Table S4, Supplementary Figures S29–S44, S46 and S47). In both cases, we detected no differences from lifetime data obtained in nuclei, except for the R52Y variant, whose values for IMS and vimentin are ~0.4 ns shorter. The R52Y side chain is exposed outside the fluorogen-binding pocket of FAST, interacting directly with the solvent-exposed pyridine group of **N871b** [29]. Thus, the R52Y/**N871b** complex appears to be the most environment sensitive.

### 3.3. FLIM-Based Visualization of Multiple Intracellular Targets

**Figure 3:**
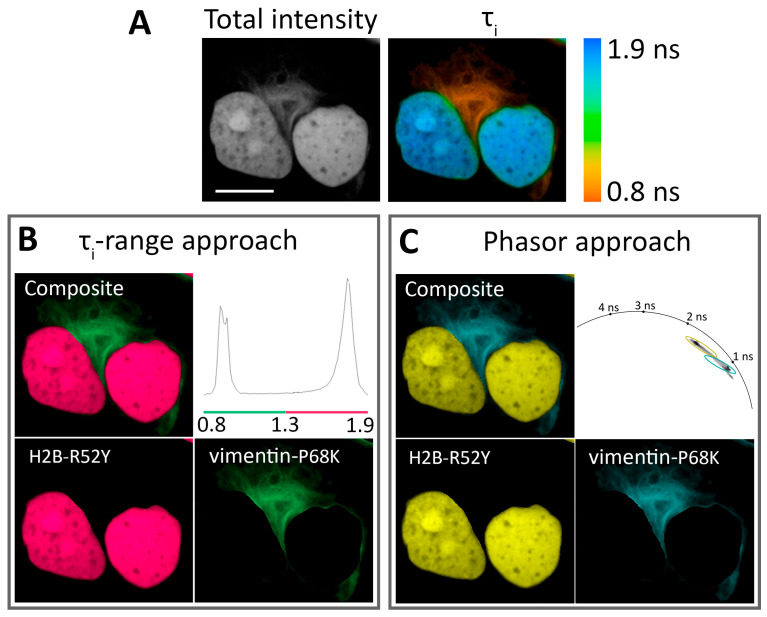
Application of **N871b** fluorogen for FLIM multiplexing with two FAST variants co-expressed in live Hela Kyoto cells: H2B-R52Y and vimentin-P68K. (**A**) Total intensity and color-coded FLIM image with bi-exponential fit (τ_i_) of **N871b** in complex with FAST variants. Color coding represents intensity-weighted lifetime (τ_i_), and is specified on the right; (**B**) results of color assignment to τi ranges of 0.8–1.3 ns for vimentin-P68K and 1.3–1.9 ns for H2B-R52Y (the composite and individual localizations are shown); (**C**) results of color assignments to phasor clusters specific to R52Y and P68K complexes with **N871b** (the composite and individual localizations are shown). Representative image. Scale bar—20 μm.

Since labeling multiple targets allows for the simultaneous monitoring of several intracellular processes, we tried to resolve two different FAST variants expressed simultaneously in HeLa Kyoto cells as H2B and vimentin fuses (Figure 3, Supplementary Figures S48–S59). The best results were achieved with pairs H2B-R52Y/vimentin-F62L and H2B-R52Y/vimentin-P68K. Both pairs showed distinct peaks in parameter distribution histograms and distinct lifetime clusters on the phasor plots, which correlated well with the largest differences in lifetime of these variants at these localizations. An example of pair decomposition with τ_i_-range and phasor approaches is presented in Figure 3. In the case of the τ_i_-range approach, a color was assigned for areas of imaged cells characterized by τ_i_ within a range specific to the FAST/**N871b** complex. In the phasor-based approach, the same algorithm was applied to lifetime clusters on phasor space (Supplementary Figures S45 and S47).

The pairs H2B-D65K/vimentin-F62L, H2B-D65K/vimentin-P68K, H2B-D65K/vimentin-R52Y, and H2B-P68K/vimentin-F62L showed moderate separation efficiency due to lower contrast in lifetimes (Supplementary Figures S50–S57). The pair H2B-F62L/vimentin-R52Y showed the worst contrast due to the lack of specified peaks and lifetime clusters (Supplementary Figures S48 and S49).

Clear, separate peaks on the τ_i_ histograms of co-expressed H2B-R52Y/vimentin-F62L, H2B-R52Y/vimentin-P68K, and H2B-P68K/vimentin-F62L prompted us to co-express these three FAST variants simultaneously as H2B-R52Y (nuclei), IMS-F62L (mitochondria), and vimentin-P68K fuses in live HeLa Kyoto cells.

**Figure 4:**
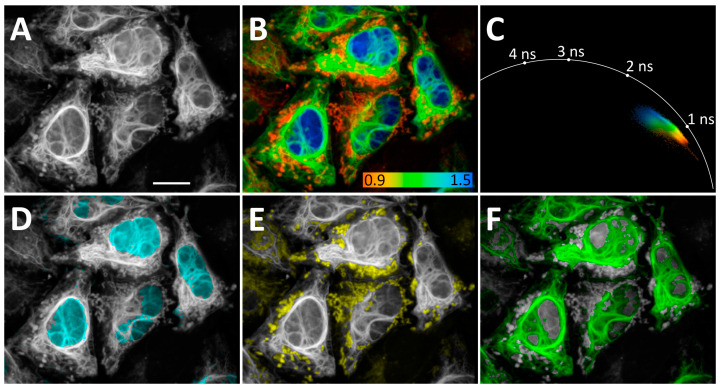
Application of **N871b** fluorogen for FLIM multiplexing with three co-expressing FAST variants in live Hela Kyoto cells: H2B-R52Y (nuclei), IMS-F62L (mitochondria), and vimentin-P68K. (**A**) Total intensity image; (**B**) FLIM image. Color coding represents intensity-weighted lifetime (τ_i_), ns; (**C**) phasor plot. Colors match color coding on B; (**D**–**F**) results of color assignments to phasor clusters specific to **N871b** complexes with H2B-R52Y (**D**), IMS-F62L (**E**), and vimentin-P68K (**F**). Each cluster was colored in one plain color independently from color coding on B. Representative image. Scale bar—20 μm.

In this case, we separated FAST localizations using only a non-fitting phasor approach, shaping phasor clusters based on the characteristic lifetime cloud of each FAST variant in phasor space and the expected morphology of imaged organelles (Figure 4, phasor clusters for D–F are represented in Supplementary Figure S60). This allowed us to separate localizations even in overlapping regions. Nevertheless, the demand for visually guided shaping of clusters may become a drawback when organelle morphology is less predictable.

## 4. Conclusions

This study presents the red-shifted FLIM multiplexing system for live cells, based on FAST point mutation variants and the **N871b** fluorogen. We demonstrated that among 17 previously proposed FAST point mutants, several variants with different fluorescence lifetimes of **N871b** fluorogen complexes can be selected. More detailed testing in living cells allows us to select three of them (F62L, P68K, and R52Y with lifetimes of about 1.009, 1.114, and 1.926 ns, respectively), which allows the clear distinction of up to three overlapping cellular compartments. It is interesting to note that variants used in complexes with **N871b** are different from those proposed for FLIM multiplexing in the green channel (R52K, P68T, and F62L), which were reported earlier for arylidene-rhodanine-based fluorogens (Figure 1) [27]. Thus, the use of various fluorogens in pairs with FAST variants in FLIM microscopy requires individual fine-tuning by introducing point substitutions into protein hot spots. The quantum yields of **N871b** complexes are similar to many popular red fluorescent proteins. The described system is available for further multiplexing at emission wavelengths below 580 nm and above 650 nm.

## Figures and Tables

**Table 1:** Optical properties (see Goncharuk et al. [29]) and fluorescence lifetimes of selected FAST variants in complexes with **N871b** obtained in vitro and *in cellulo*. Data is shown as mean ± SD, number of individual analyzed cells was ≥17 (the exact number of analyzed cells is provided in Supplementary Tables S3 and S4).

	In Vitro Measurements						*In Cellulo* Measurements		
Variant	K_d_, µM	ε, M^−1^·cm^−1^	FQY, %	Brightness	τ_m_, ns *	τ_i_, ns *	H2B Fuse τ_i_, ns *	IMS Fuse τ_i_, ns *	Vimentin Fuse τ_i_, ns *
FAST (original)	0.33 ± 0.01	27,000 ± 410	26 ± 1.4	7000 ± 480	2.65	2.79	1.407 ± 0.064	-	-
D65K	0.25 ± 0.01	27,500 ± 410	26 ± 2.1	7100 ± 680	2.53	2.70	1.342 ± 0.059	1.254 ± 0.067	1.295 ± 0.043
F62L	0.55 ± 0.08	27,500 ± 410	19 ± 0.9	5250 ± 330	2.28	2.44	1.009 ± 0.054	1.060 ± 0.170	0.897 ± 0.046
P68K	0.22 ± 0.01	26,500 ± 400	18 ± 0.7	4750 ± 260	1.94	2.13	1.114 ± 0.079	0.978 ± 0.032	1.027 ± 0.051
R52Y	0.24 ± 0.02	29,000 ± 440	29 ± 1.5	8350 ± 560	2.90	2.90	1.926 ± 0.092	1.512 ± 0.072	1.591 ± 0.070

* Multicomponent fitting data may be averaged in two ways: the amplitude-weighted average lifetime (τ_m_) and the intensity-weighted average lifetime (τ_i_) (see Section 2 for more details). Both parameters were calculated here. The information provided in the main text below is represented only as intensity-weighted lifetimes. Images processed using both parameters are presented in supporting material (Supplementary Figures S19–S60).

## Data Availability

Data is available on request.

## Supplementary Materials

The following supporting information can be downloaded at: https://www.mdpi.com/article/10.3390/bios15050274/s1.

## Abbreviations

The following abbreviations are used in this manuscript:

FLIM	Fluorescence-lifetime imaging microscopy
SLP	Self-labeling protein
FAST	Fluorescence-Activating and Absorption-Shifting Tag
TCSPC	Time-correlated single-photon counting
