# Supplementary material for: Fluorescence Lifetime Multiplexing with Fluorogen-Activating FAST Protein Variants and Red-Shifted Arylidene–Imidazolone Derivative as Fluorogen
Source: Biosensors (Basel). 2025 Apr 29;15(5):274. doi: 10.3390/bios15050274 (PMC12109237; doi:10.3390/bios15050274)
Supplement: Supplementary file 1 [file biosensors-15-00274-s001.zip › biosensors-3528135-supplementary.pdf]

Contents

1 Amino acid sequences

2 Primary fluorescence lifetime screening *in vitro*

3 Fluorescence lifetime measurements in cells

4 FLIM-based visualization of multiple intracellular targets

5 Compound N871b description

6 References

1 Amino acid sequences

Table S1. Amino acid sequences of FAST variants[1]

**FAST (original)**	(M)EHVAFGSEDIENTLAKMDDGQLDGLAFGAIQLDGDGNILQYNAAEGDITGRDP<br>KQVIGKNFFKDVAPGTDSPEFYGKFKEGVASGNLNTMFEWMIPTSRGPTKVKVHM<br>KKALSGDSYWVFVKRV(GGGHHHHHH)
**D65K**	(M)EHVAFGSEDIENTLAKMDDGQLDGLAFGAIQLDGDGNILQYNAAEGDITGRDP<br>KQVIGKNFFK**K**VAPGTDSPEFYGKFKEGVASGNLNTMFEWMIPTSRGPTKVKVHM<br>KKALSGDSYWVFVKRV(GGGHHHHHH)
**D65R**	(M)EHVAFGSEDIENTLAKMDDGQLDGLAFGAIQLDGDGNILQYNAAEGDITGRDP<br>KQVIGKNFFK**R**VAPGTDSPEFYGKFKEGVASGNLNTMFEWMIPTSRGPTKVKVHM<br>KKALSGDSYWVFVKRV(GGGHHHHHH)
**F62L**	(M)EHVAFGSEDIENTLAKMDDGQLDGLAFGAIQLDGDGNILQYNAAEGDITGRDP<br>KQVIGKN**L**FKDVAPGTDSPEFYGKFKEGVASGNLNTMFEWMIPTSRGPTKVKVHM<br>KKALSGDSYWVFVKRV(GGGHHHHHH)
**P68K**	(M)EHVAFGSEDIENTLAKMDDGQLDGLAFGAIQLDGDGNILQYNAAEGDITGRDP<br>KQVIGKNFFKDVA**K**GTDSPEFYGKFKEGVASGNLNTMFEWMIPTSRGPTKVKVHM<br>KKALSGDSYWVFVKRV(GGGHHHHHH)
**P68R**	(M)EHVAFGSEDIENTLAKMDDGQLDGLAFGAIQLDGDGNILQYNAAEGDITGRDP<br>KQVIGKNFFKDVA**R**GTDSPEFYGKFKEGVASGNLNTMFEWMIPTSRGPTKVKVHM<br>KKALSGDSYWVFVKRV(GGGHHHHHH)
**P68T**	(M)EHVAFGSEDIENTLAKMDDGQLDGLAFGAIQLDGDGNILQYNAAEGDITGRDP<br>KQVIGKNFFKDVA**T**GTDSPEFYGKFKEGVASGNLNTMFEWMIPTSRGPTKVKVHM<br>KKALSGDSYWVFVKRV(GGGHHHHHH)
**P73S**	(M)EHVAFGSEDIENTLAKMDDGQLDGLAFGAIQLDGDGNILQYNAAEGDITGRDP<br>KQVIGKNFFKDVAPGTDS**S**EFYGKFKEGVASGNLNTMFEWMIPTSRGPTKVKVHM<br>KKALSGDSYWVFVKRV(GGGHHHHHH)
**R52A**	(M)EHVAFGSEDIENTLAKMDDGQLDGLAFGAIQLDGDGNILQYNAAEGDITG**A**DP<br>KQVIGKNFFKDVAPGTDSPEFYGKFKEGVASGNLNTMFEWMIPTSRGPTKVKVHM<br>KKALSGDSYWVFVKRV(GGGHHHHHH)
**R52F**	(M)EHVAFGSEDIENTLAKMDDGQLDGLAFGAIQLDGDGNILQYNAAEGDITG**F**DP<br>KQVIGKNFFKDVAPGTDSPEFYGKFKEGVASGNLNTMFEWMIPTSRGPTKVKVHM<br>KKALSGDSYWVFVKRV(GGGHHHHHH)
**R52K**	(M)EHVAFGSEDIENTLAKMDDGQLDGLAFGAIQLDGDGNILQYNAAEGDITG**K**DP<br>KQVIGKNFFKDVAPGTDSPEFYGKFKEGVASGNLNTMFEWMIPTSRGPTKVKVHM<br>KKALSGDSYWVFVKRV(GGGHHHHHH)
**R52L**	(M)EHVAFGSEDIENTLAKMDDGQLDGLAFGAIQLDGDGNILQYNAAEGDITG**L**DP<br>KQVIGKNFFKDVAPGTDSPEFYGKFKEGVASGNLNTMFEWMIPTSRGPTKVKVHM<br>KKALSGDSYWVFVKRV(GGGHHHHHH)
**R52Y**	(M)EHVAFGSEDIENTLAKMDDGQLDGLAFGAIQLDGDGNILQYNAAEGDITG**Y**DP<br>KQVIGKNFFKDVAPGTDSPEFYGKFKEGVASGNLNTMFEWMIPTSRGPTKVKVHM<br>KKALSGDSYWVFVKRV(GGGHHHHHH)
**S99E**	(M)EHVAFGSEDIENTLAKMDDGQLDGLAFGAIQLDGDGNILQYNAAEGDITGRDP<br>KQVIGKNFFKDVAPGTDSPEFYGKFKEGVASGNLNTMFEWMIPT**E**RGPTKVKVHM<br>KKALSGDSYWVFVKRV(GGGHHHHHH)
**S99K**	(M)EHVAFGSEDIENTLAKMDDGQLDGLAFGAIQLDGDGNILQYNAAEGDITGRDP<br>KQVIGKNFFKDVAPGTDSPEFYGKFKEGVASGNLNTMFEWMIPT**K**RGPTKVKVHM<br>KKALSGDSYWVFVKRV(GGGHHHHHH)
**S99R**	(M)EHVAFGSEDIENTLAKMDDGQLDGLAFGAIQLDGDGNILQYNAAEGDITGRDP<br>KQVIGKNFFKDVAPGTDSPEFYGKFKEGVASGNLNTMFEWMIPT**R**RGPTKVKVHM<br>KKALSGDSYWVFVKRV(GGGHHHHHH)
**V107I**	(M)EHVAFGSEDIENTLAKMDDGQLDGLAFGAIQLDGDGNILQYNAAEGDITGRDP<br>KQVIGKNFFKDVAPGTDSPEFYGKFKEGVASGNLNTMFEWMIPTSRGPTKVK**I**HM<br>KKALSGDSYWVFVKRV(GGGHHHHHH)

2 Primary fluorescence lifetime screening *in vitro*

Table S2. Properties of FAST mutants complexes with **N871b**. Mutant variants, chosen for further investigation *in cellulo*, are highlighted in cyan.

Mutant	Optical properties[a]						Lifetime properties						
	K_D, µM	ε, $M^{-1}\cdot cm^{-1}$	Fluorescence quantum yield (FQY), %	Brightness (=ε·FQY)	Absorbance maxima position, nm	Emission maxima position, nm	τ_1, ns	A_1, %	τ_2, ns	A_2, %	τ_m, ns	τ_i, ns	χ^2
**FAST (original)**	0.33±0.01	27000±410	26±1.4	7000±480	553	609	0.24	6	2.8	94	2.65	2.79	1.292
**D65K**	0.25±0.01	27500±410	26±2.1	7100±680	555	607	0.13	7	2.71	93	2.53	2.70	1.144
**D65R**	0.34±0.04	27000±410	24±1.2	6450±420	554	609	0.33	6	2.76	94	2.61	2.74	1.214
**F62L**	0.55±0.08	27500±410	19±0.9	5250±330	552	611	0.23	8	2.46	92	2.28	2.44	1.230
**P68K**	0.22±0.01	26500±400	18±0.7	4750±260	548	610	0.09	10	2.14	90	1.94	2.13	1.091
**P68R**	0.31±0.03	27000±410	23±0.4	6100±200	547	609	0.15	9	2.62	91	2.40	2.61	1.174
**P68T**	0.41±0.03	26500±400	17±1.5	4550±470	527	604	0.17	15	2.34	85	2.01	2.31	1.126
**P73S**	0.17±0.01	28000±420	20±1.2	5600±420	558	610	0.96	30	2.47	70	2.02	2.25	1.120
**R52A**	0.79±0.05	28500±430	27±1.0	7700±400	558	607	0.25	5	2.9	95	2.77	2.89	1.195
**R52F**	0.54±0.04	28000±420	23±0.4	6450±210	557	606	0.20	10	2.7	90	2.45	2.68	1.185
**R52K**	0.42±0.02	29500±440	24±1.2	7100±460	549	605	0.22	7	2.86	93	2.68	2.84	1.156
**R52L**	0.27±0.01	30000±450	29±1.7	8750±640	556	604	0.35	7	2.83	93	2.66	2.81	1.057
**R52Y**	0.24±0.02	29000±440	29±1.5	8350±560	558	605	0.16	6	3.07	94	2.90	3.06	1.037
**S99E**	0.21±0.01	28500±430	25±1.1	7050±420	556	608	0.21	6	2.8	94	2.64	2.79	1.143
**S99K**	0.27±0.01	28000±420	25±0.3	7050±190	556	609	0.20	6	2.76	94	2.61	2.75	1.090
**S99R**	0.24±0.01	28500±430	24±1.2	6800±440	555	608	0.19	7	2.79	93	2.61	2.78	1.189
**V107I**	0.38±0.05	26500±400	27±3.2	7150±950	555	607	0.44	5	2.86	95	2.74	2.84	1.143

a – represented data was obtained in previous work[1]

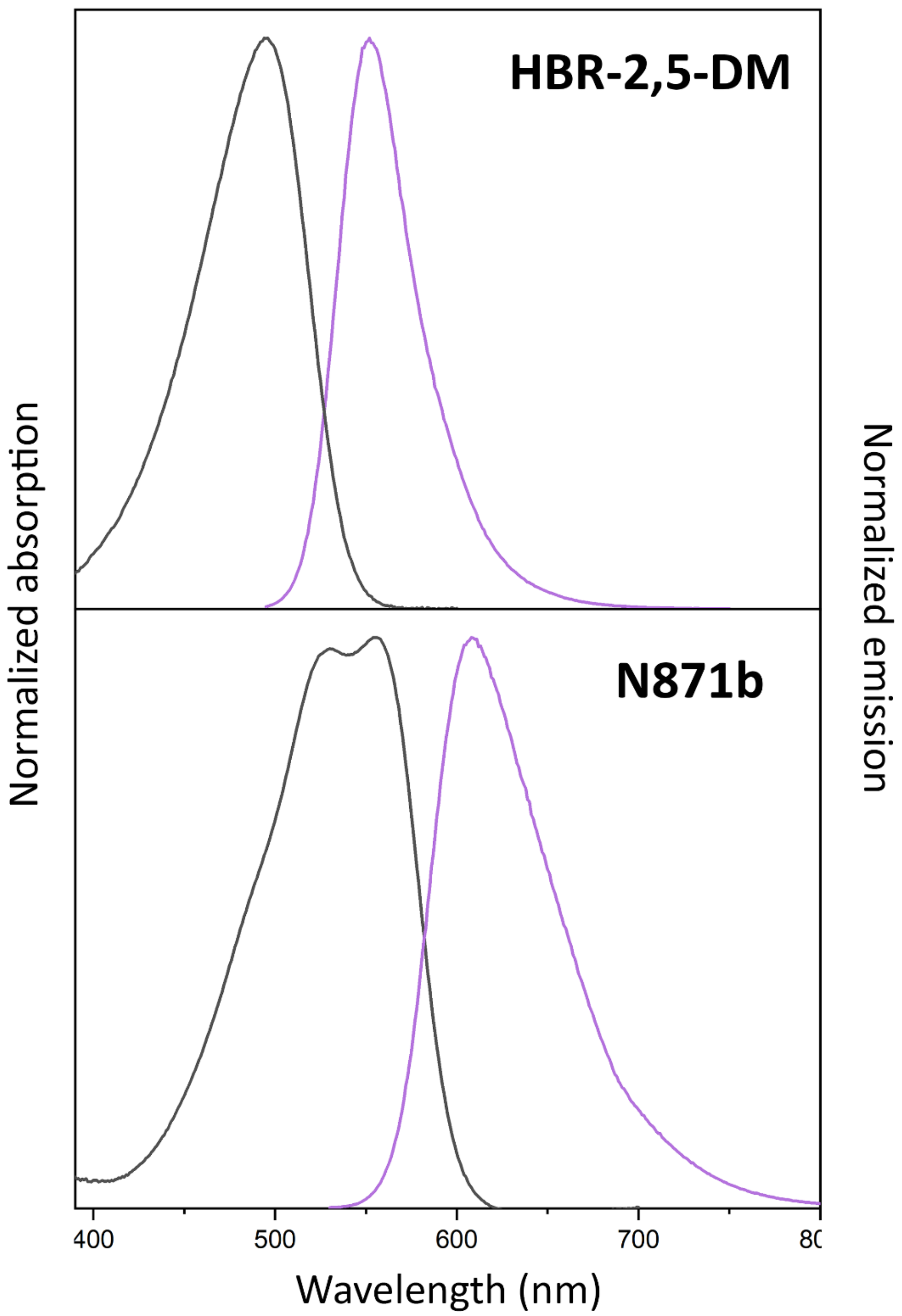

Figure S1. The absorption and emission spectra of **HBR-2,5-DM** (previous work) and **N871b** fluorogens with original FAST protein.

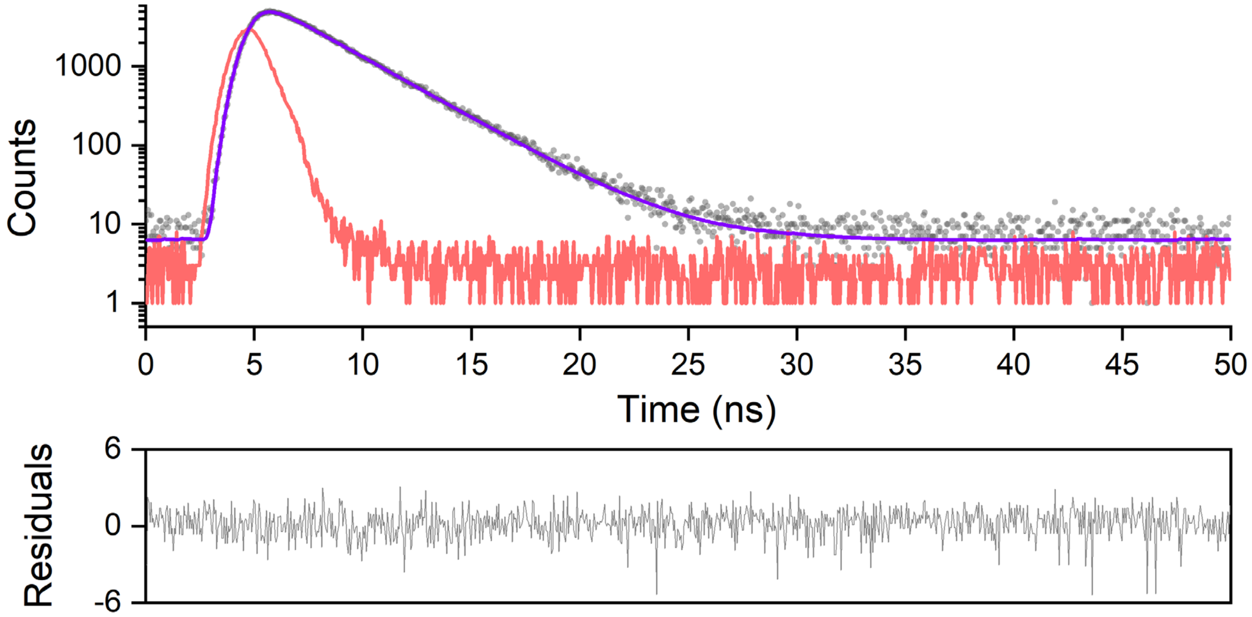

Figure S2. Fluorescence decay kinetics of the **N871b** bound by **original** FAST variant in bacterial lysate (bi-exponential fit). Gray dots represent experimental decay data (photon arrivals), blue line shows exponential fit data, red curve denotes instrument function IRF. Residuals of fitting are shown below.

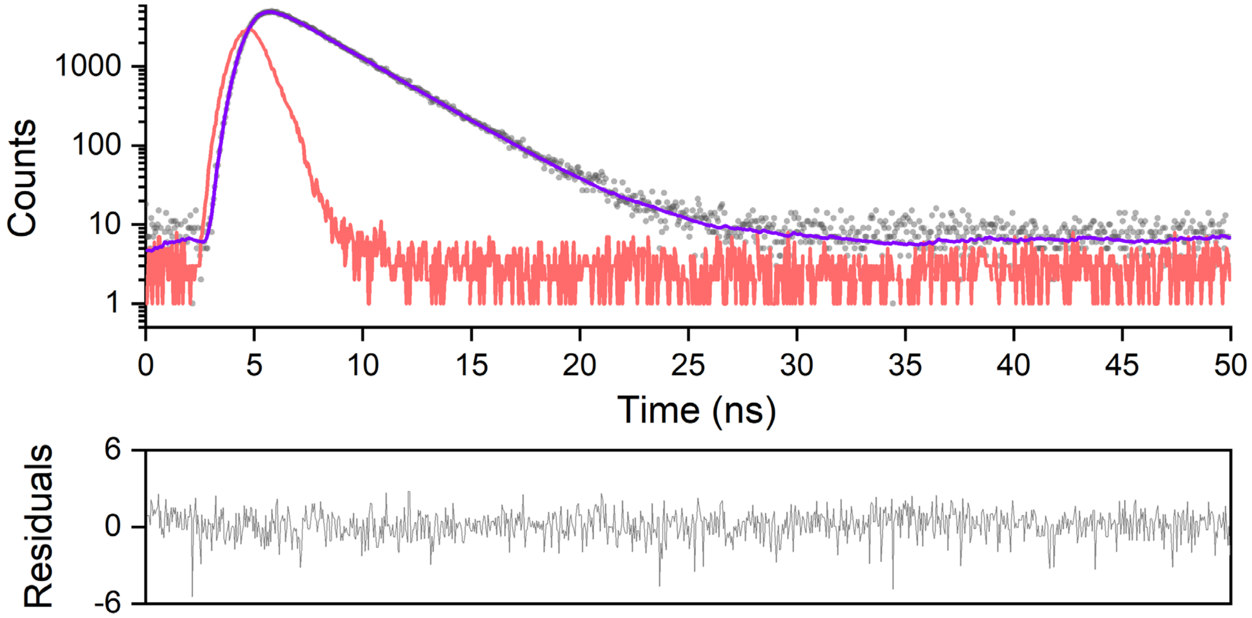

Figure S3. Fluorescence decay kinetics of the **N871b** bound by **D65K** FAST variant in bacterial lysate (bi-exponential fit). Gray dots represent experimental decay data (photon arrivals), blue line shows exponential fit data, red curve denotes instrument function IRF. Residuals of fitting are shown below.

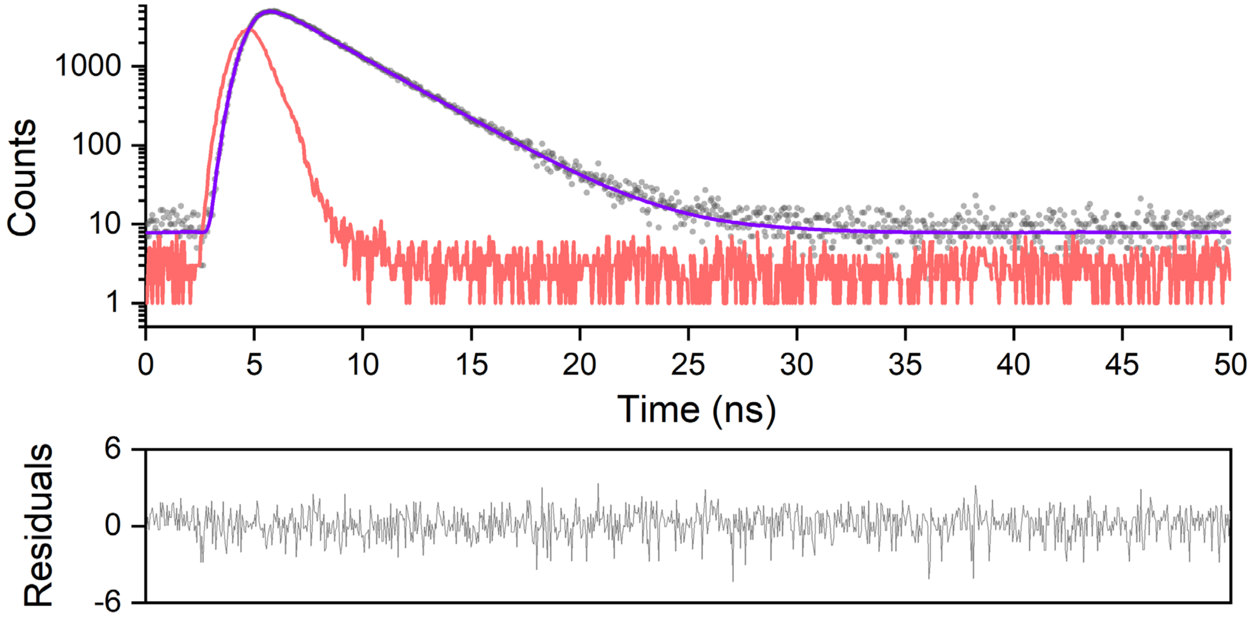

Figure S4. Fluorescence decay kinetics of the **N871b** bound by **D65R** FAST variant in bacterial lysate (bi-exponential fit). Gray dots represent experimental decay data (photon arrivals), blue line shows exponential fit data, red curve denotes instrument function IRF. Residuals of fitting are shown below.

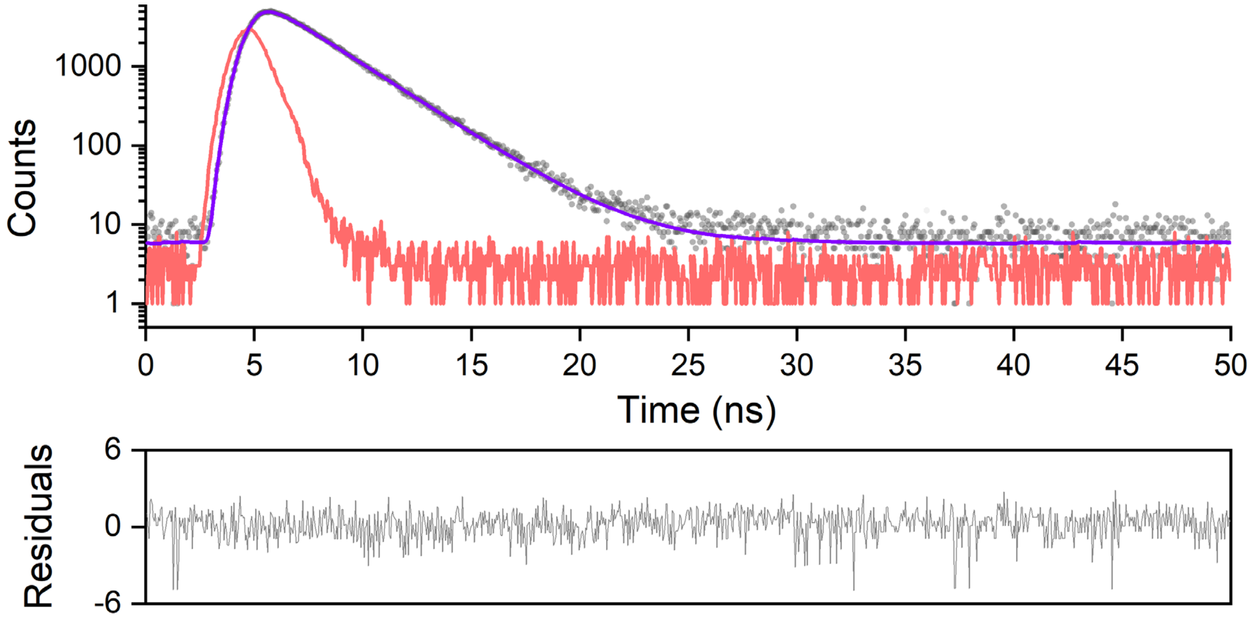

Figure S5. Fluorescence decay kinetics of the **N871b** bound by **F62L** FAST variant in bacterial lysate (bi-exponential fit). Gray dots represent experimental decay data (photon arrivals), blue line shows exponential fit data, red curve denotes instrument function IRF. Residuals of fitting are shown below.

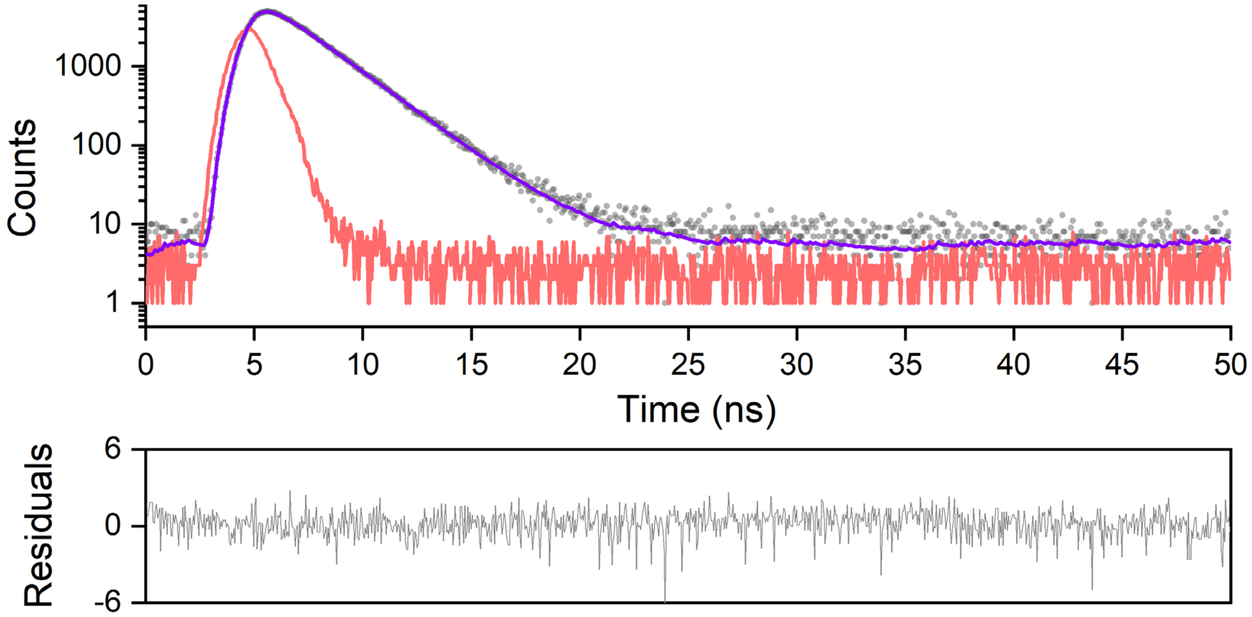

Figure S6. Fluorescence decay kinetics of the **N871b** bound by **P68K** FAST variant in bacterial lysate (bi-exponential fit). Gray dots represent experimental decay data (photon arrivals), blue line shows exponential fit data, red curve denotes instrument function IRF. Residuals of fitting are shown below.

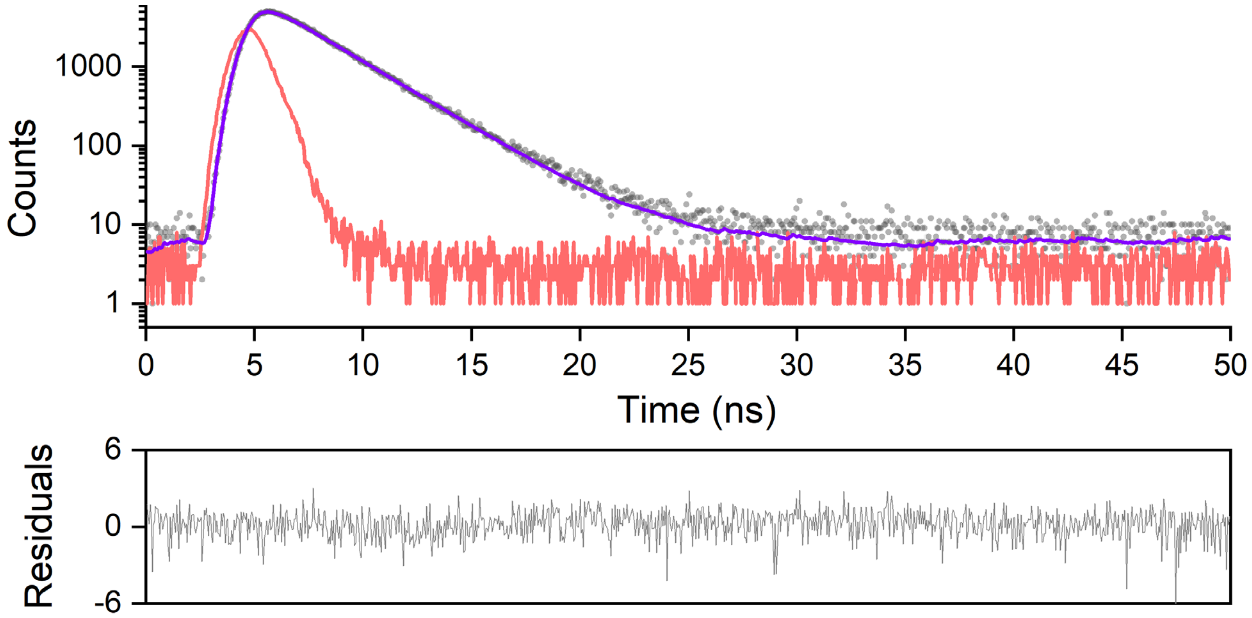

Figure S7. Fluorescence decay kinetics of the **N871b** bound by **P68R** FAST variant in bacterial lysate (bi-exponential fit). Gray dots represent experimental decay data (photon arrivals), blue line shows exponential fit data, red curve denotes instrument function IRF. Residuals of fitting are shown below.

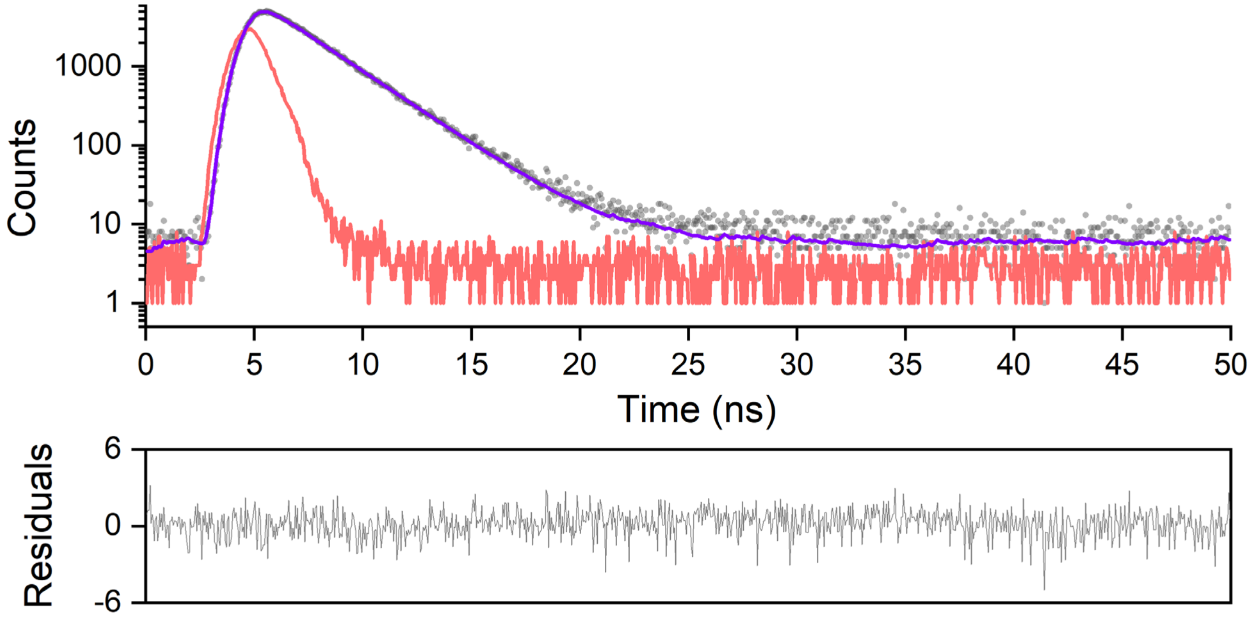

Figure S8. Fluorescence decay kinetics of the **N871b** bound by **P68T** FAST variant in clarified lysate (bi-exponential fit). Gray dots represent experimental decay data (photon arrivals), blue line shows exponential fit data, red curve denotes instrument function IRF. Residuals of fitting are shown below.

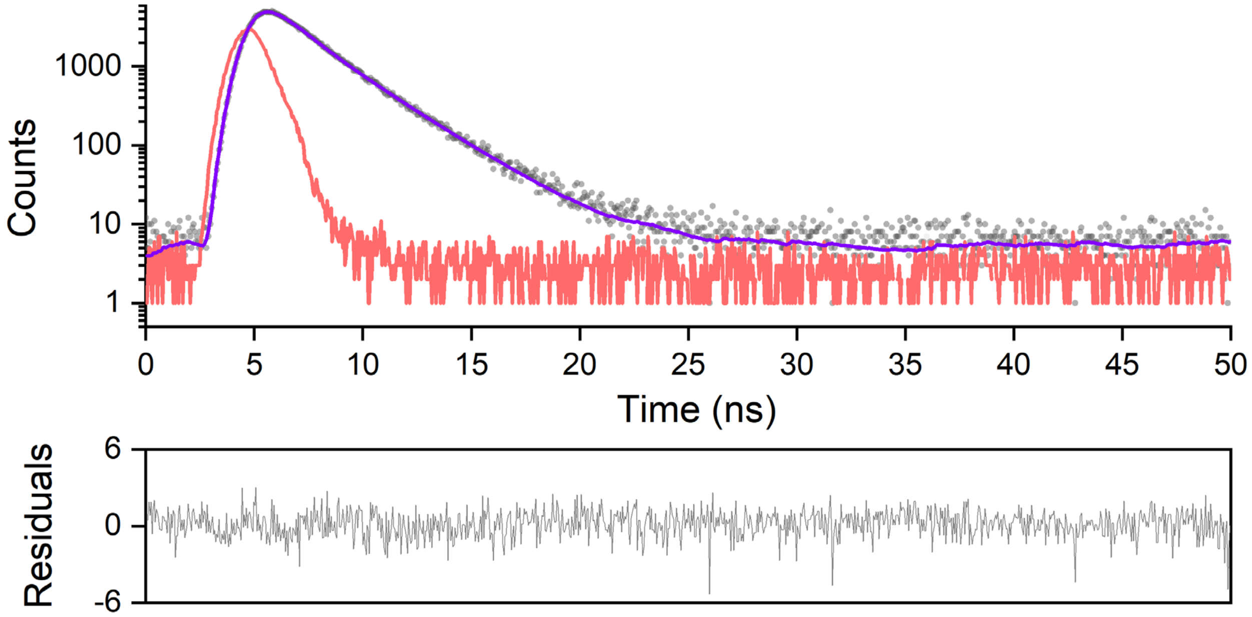

Figure S9. Fluorescence decay kinetics of the **N871b** bound by **P73S** FAST variant in bacterial lysate (bi-exponential fit). Gray dots represent experimental decay data (photon arrivals), blue line shows exponential fit data, red curve denotes instrument function IRF. Residuals of fitting are shown below.

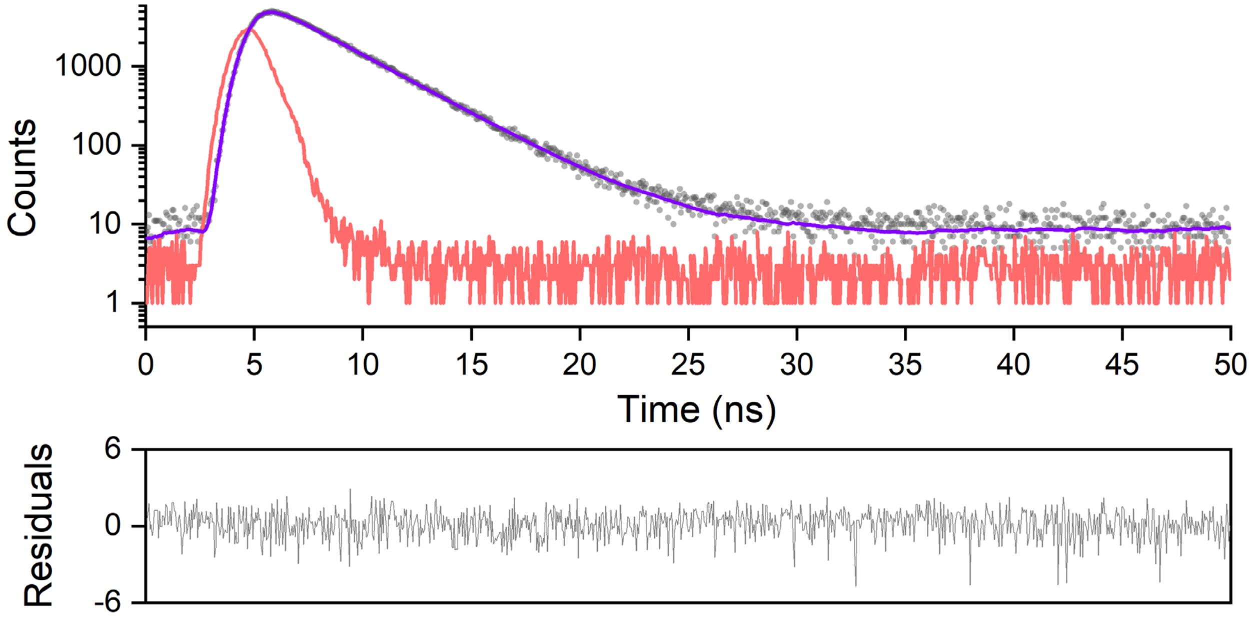

Figure S10. Fluorescence decay kinetics of the **N871b** bound by **R52A** FAST variant in bacterial lysate (bi-exponential fit). Gray dots represent experimental decay data (photon arrivals), blue line shows exponential fit data, red curve denotes instrument function IRF. Residuals of fitting are shown below.

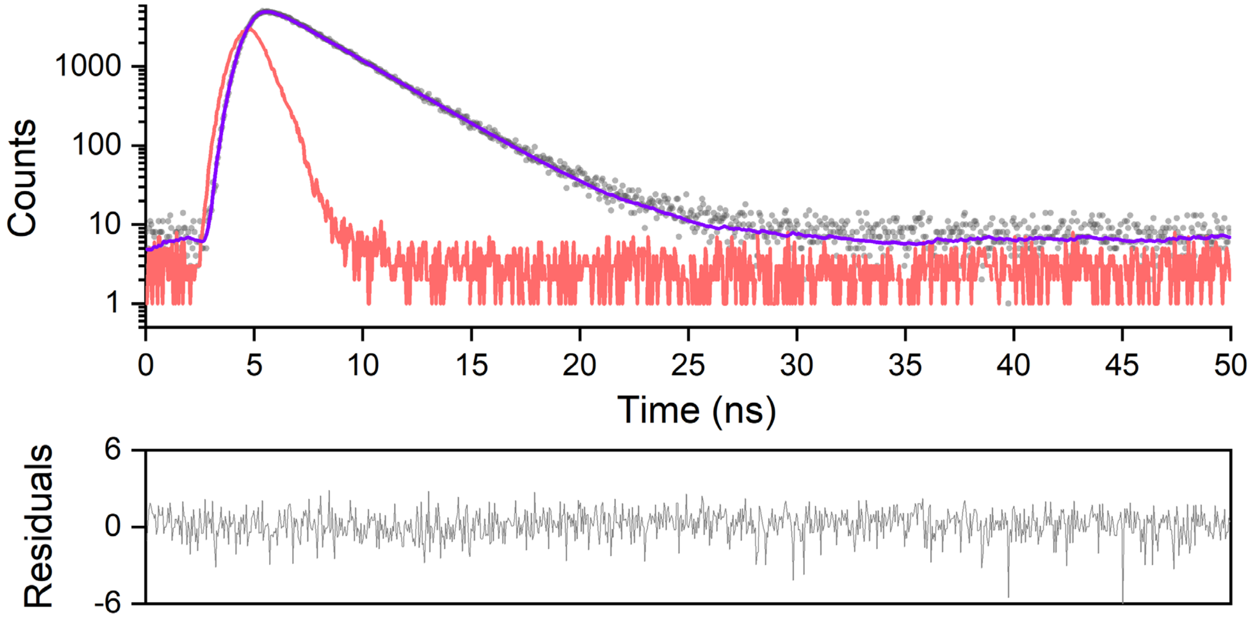

Figure S11. Fluorescence decay kinetics of the **N871b** bound by **R52F** FAST variant in bacterial lysate (bi-exponential fit). Gray dots represent experimental decay data (photon arrivals), blue line shows exponential fit data, red curve denotes instrument function IRF. Residuals of fitting are shown below.

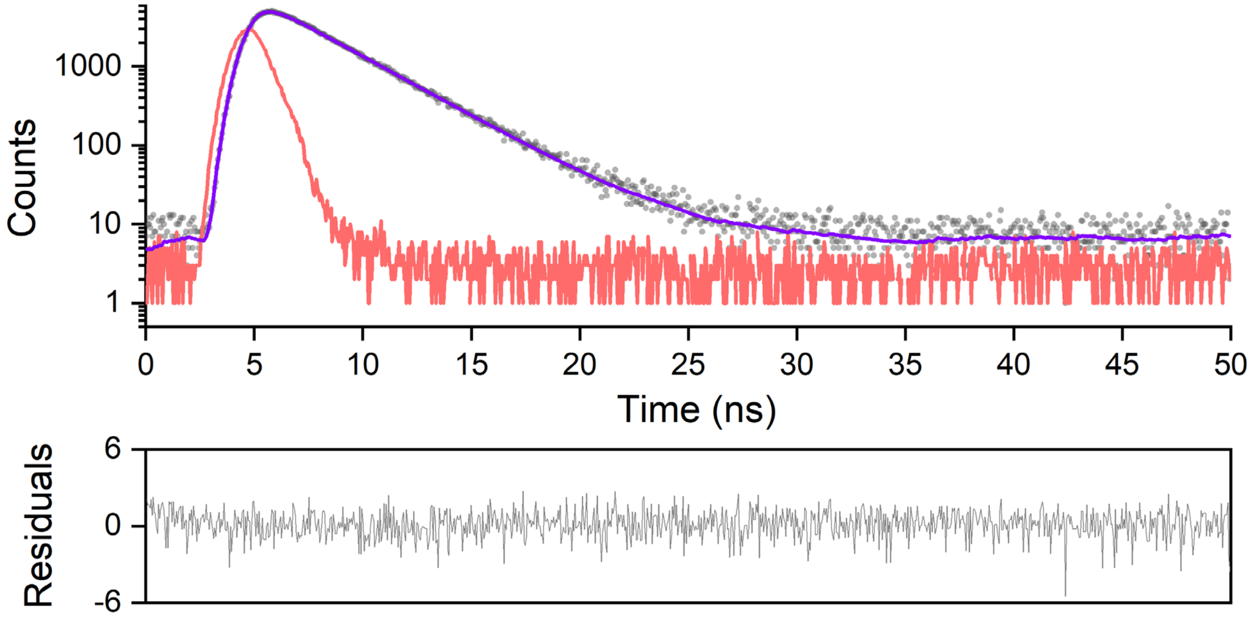

Figure S12. Fluorescence decay kinetics of the **N871b** bound by **R52K** FAST variant in bacterial lysate (bi-exponential fit). Gray dots represent experimental decay data (photon arrivals), blue line shows exponential fit data, red curve denotes instrument function IRF. Residuals of fitting are shown below.

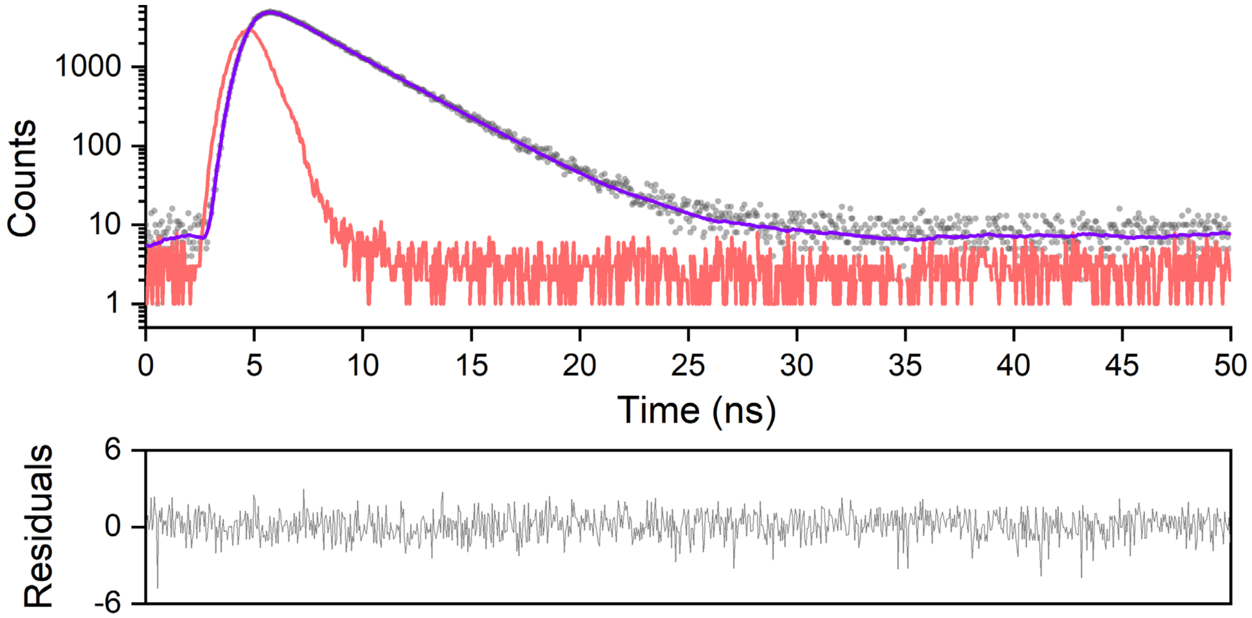

Figure S13. Fluorescence decay kinetics of the **N871b** bound by **R52L** FAST variant in bacterial lysate (bi-exponential fit). Gray dots represent experimental decay data (photon arrivals), blue line shows exponential fit data, red curve denotes instrument function IRF. Residuals of fitting are shown below.

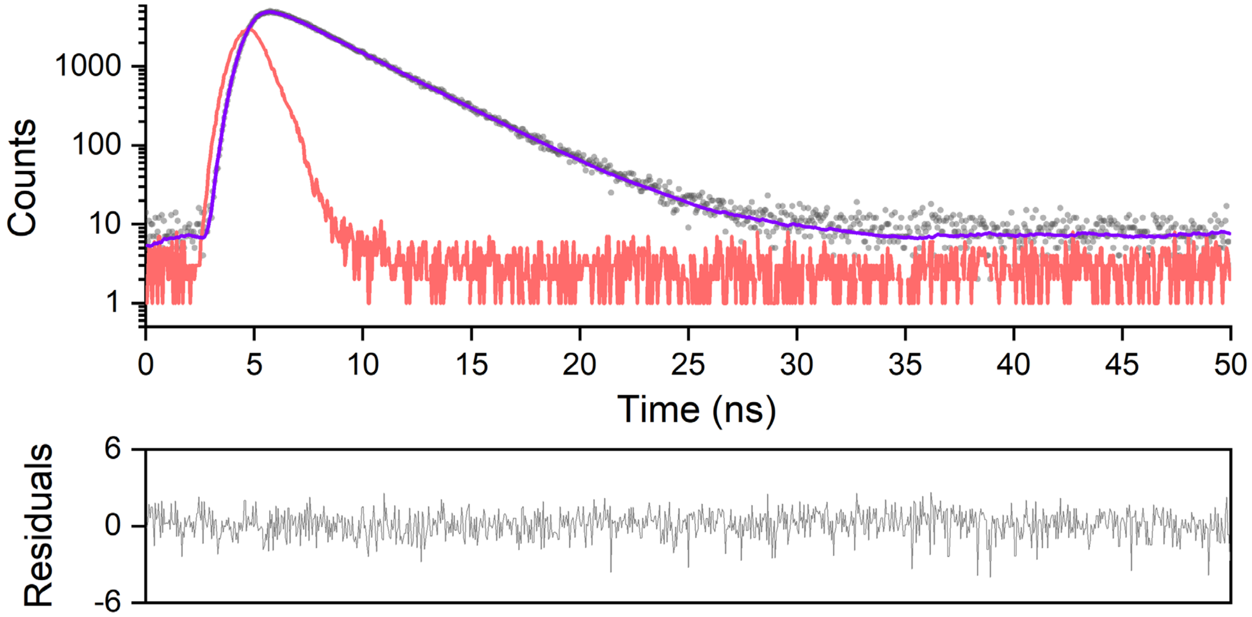

Figure S14. Fluorescence decay kinetics of the **N871b** bound by **R52Y** FAST variant in bacterial lysate (bi-exponential fit). Gray dots represent experimental decay data (photon arrivals), blue line shows exponential fit data, red curve denotes instrument function IRF. Residuals of fitting are shown below.

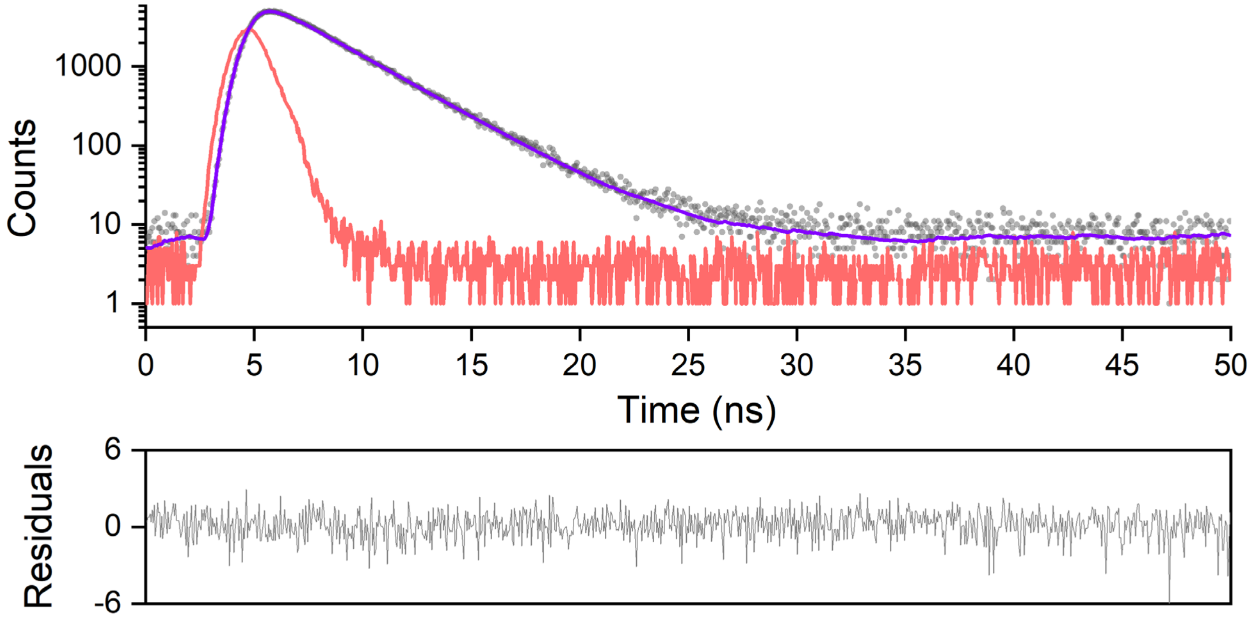

Figure S15. Fluorescence decay kinetics of the **N871b** bound by **S99E** FAST variant in bacterial lysate (bi-exponential fit). Gray dots represent experimental decay data (photon arrivals), blue line shows exponential fit data, red curve denotes instrument function IRF. Residuals of fitting are shown below.

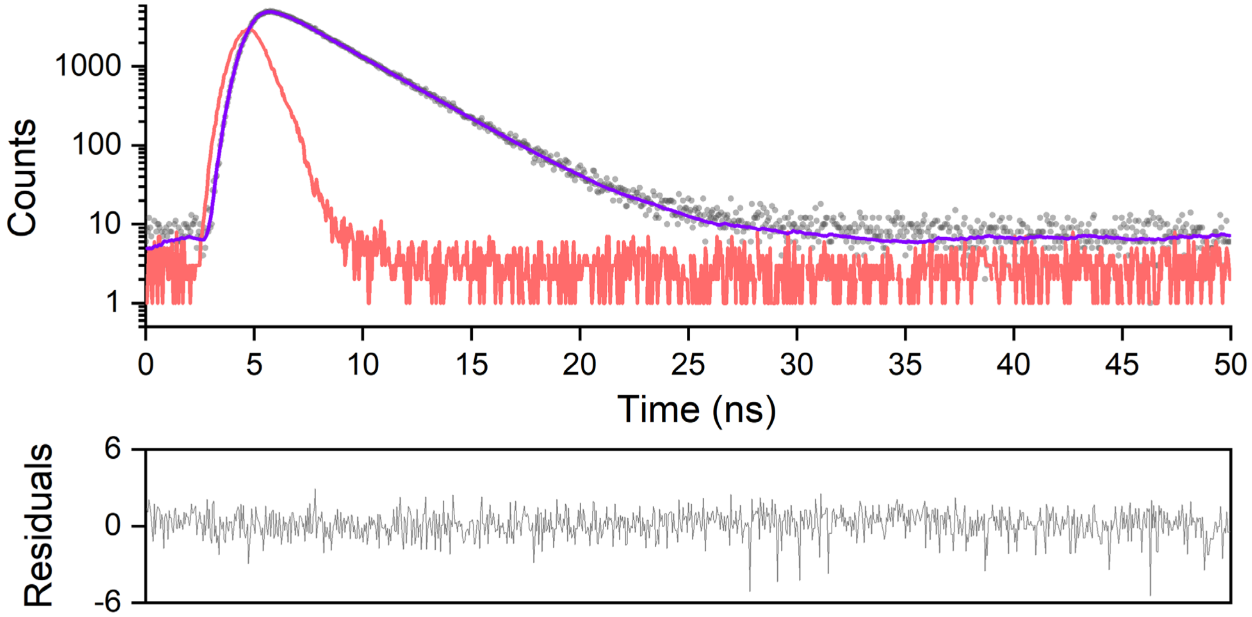

Figure S16. Fluorescence decay kinetics of the **N871b** bound by **S99K** FAST variant in bacterial lysate (bi-exponential fit). Gray dots represent experimental decay data (photon arrivals), blue line shows exponential fit data, red curve denotes instrument function IRF. Residuals of fitting are shown below.

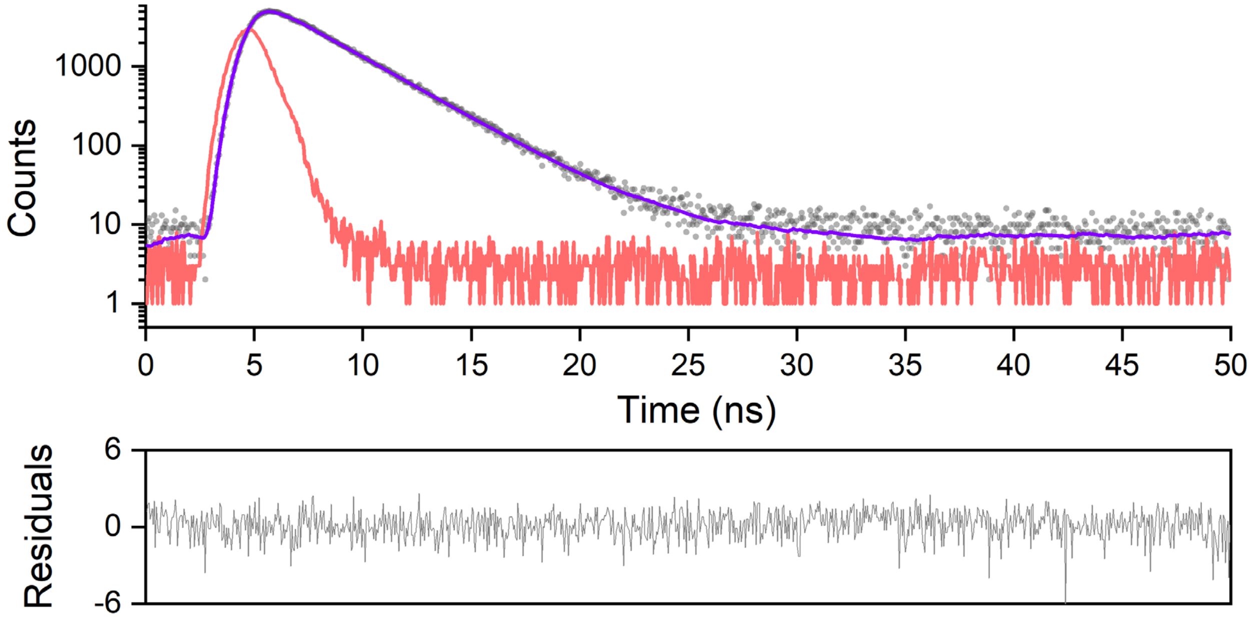

Figure S17. Fluorescence decay kinetics of the **N871b** bound by **S99R** FAST variant in bacterial lysate (bi-exponential fit). Gray dots represent experimental decay data (photon arrivals), blue line shows exponential fit data, red curve denotes instrument function IRF. Residuals of fitting are shown below.

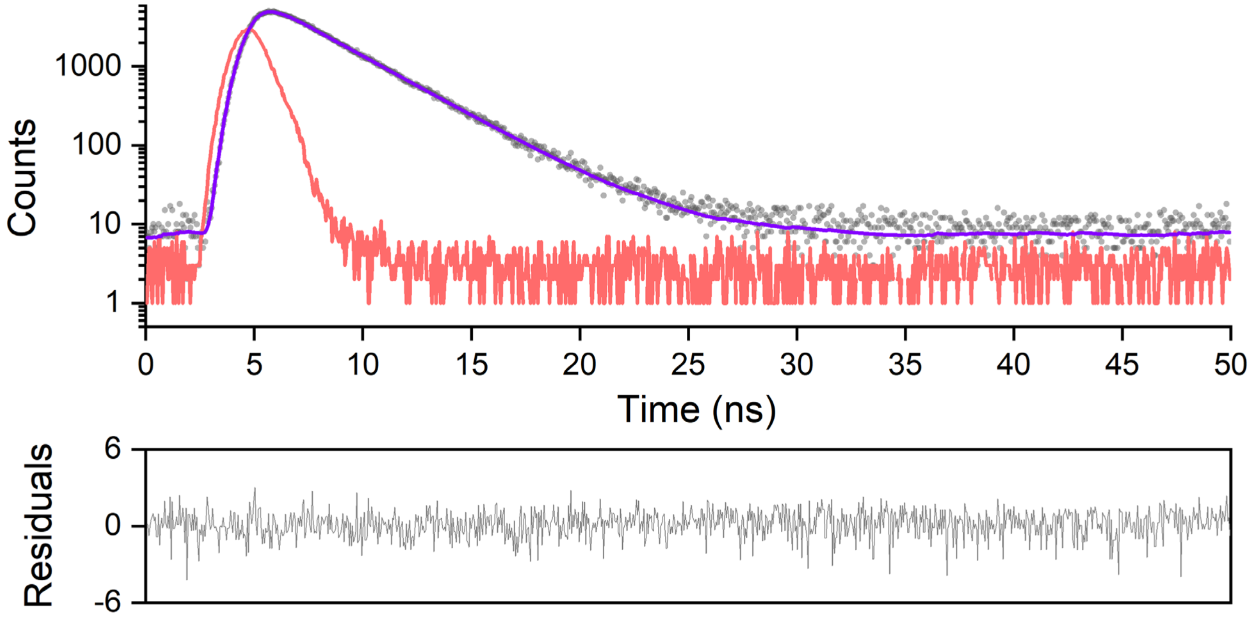

Figure S18. Fluorescence decay kinetics of the **N871b** bound by **V107I** FAST variant in bacterial lysate (bi-exponential fit). Gray dots represent experimental decay data (photon arrivals), blue line shows exponential fit data, red curve denotes instrument function IRF. Residuals of fitting are shown below.

3 Fluorescence lifetime measurements in cells

Table S3. Comparison of bi- and tri-exponential fits obtained *in cellulo* for complexes of H2B-FAST with **N871b.** *SD* is standard deviation, in all cases the number of individual cells taken for analysis is n = 30.

Fit variant	FAST variant	$\tau_1 \pm$ SD, ns	A_1, %	$\tau_2 \pm$ SD, ns	A_2, %	$\tau_3 \pm$ SD, ns	A3, %	τ_i, ns	χ^2
**Bi-exponential**	**FAST (original)**	0.604±0.047	77	2.152±0.145	23	-	-	1.407±0.064	1.13
	**D65K**	0.569±0.028	76	2.044±0.122	23	-	-	1.342±0.059	1.14
	**F62L**	0.441±0.026	82	1.703±0.140	18	-	-	1.009±0.054	1.18
	**P68K**	0.454±0.027	74	1.648±0.075	26	-	-	1.114±0.079	1.14
	**R52Y**	0.569±0.055	64	2.488±0.099	36	-	-	1.926±0.092	1.14
**Tri-exponential**	**FAST (original)**	0.526±0.034	70	1.603±0.236	22	2.769±0.394	8	1.433±0.081	1.12
	**D65K**	0.526±0.058	69	1.472±0.143	24	2.893±0.236	7	1.417±0.059	1.11
	**F62L**	0.390±0.024	73	1.061±0.153	20	2.335±0.225	7	1.065±0.044	1.13
	**P68K**	0.428±0.025	68	1.296±0.126	25	2.311±0.190	7	1.180±0.043	1.15
	**R52Y**	0.546±0.043	64	2.210±0.181	28	3.248±0.472	8	1.974±0.094	1.13

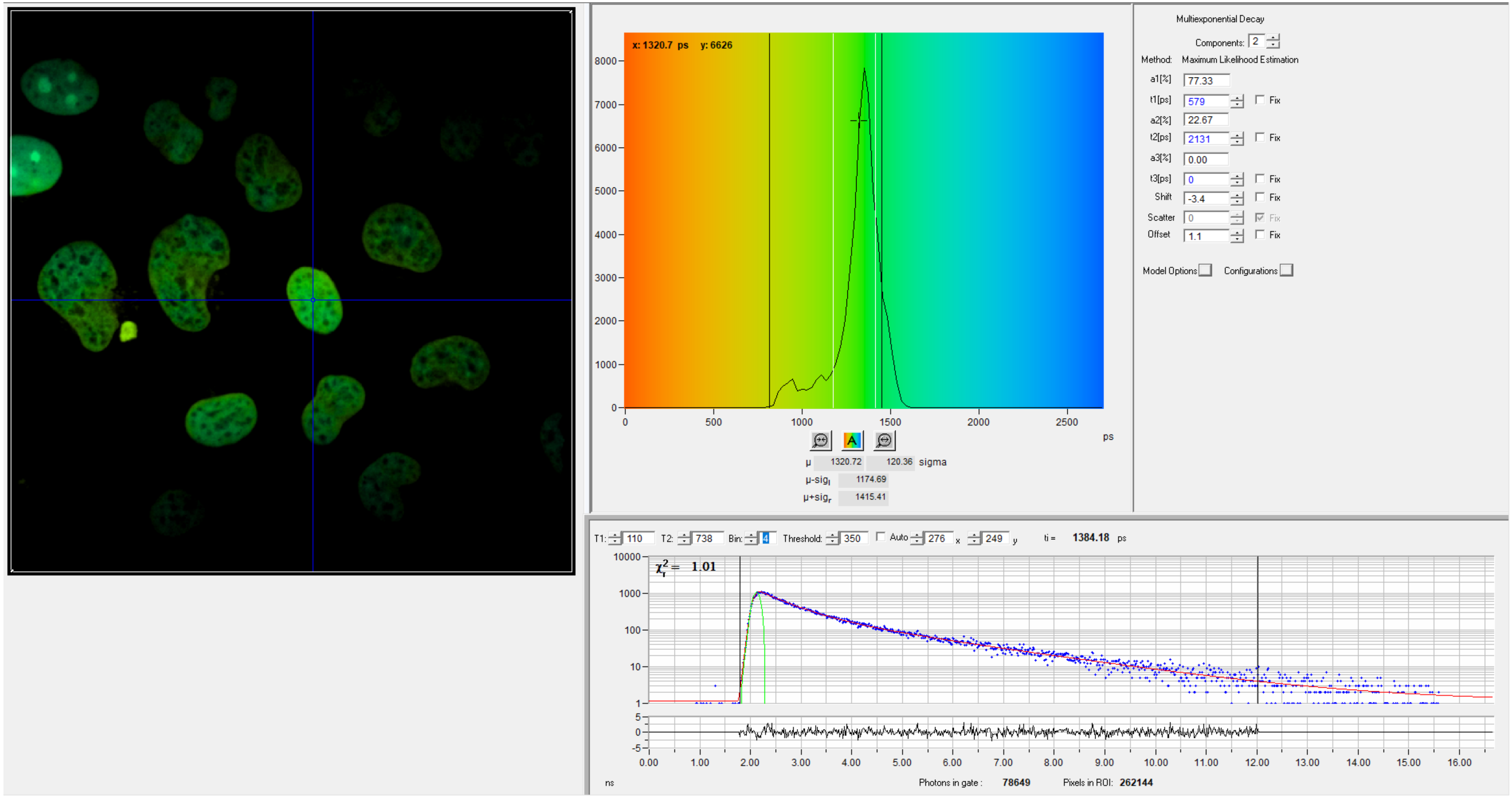

Figure S19. **H2B-FAST (original)** fuse in complex with **N871b**; bi-exponential fit; τ_i color-coding. FLIM scan and corresponding time-resolved fluorescence data analysis of life HeLa cells expressing the original FAST variant fused to histone-2B (H2B) and stained with **N871b**. A screenshot from Becker&Hickl SPCImage data acquisition and analysis window is shown. Bi-exponential fitting of decay data was performed. On the left panel is a FLIM image of HeLa nuclei, color-coded according to intensity-weighted average fluorescence lifetime in each pixel (τ_i). A histogram on the upper right panel displays distribution of τ_i and color legend. The right panel represents bi-exponential fitting model used to fit data and fitting results. The lower data shows data on fluorescence decay. Blue dots represent experimental decay data, red line represents bi-exponential fit, green line represents instrument response function (IRF), fitting residuals shown as black graph below main data plot.

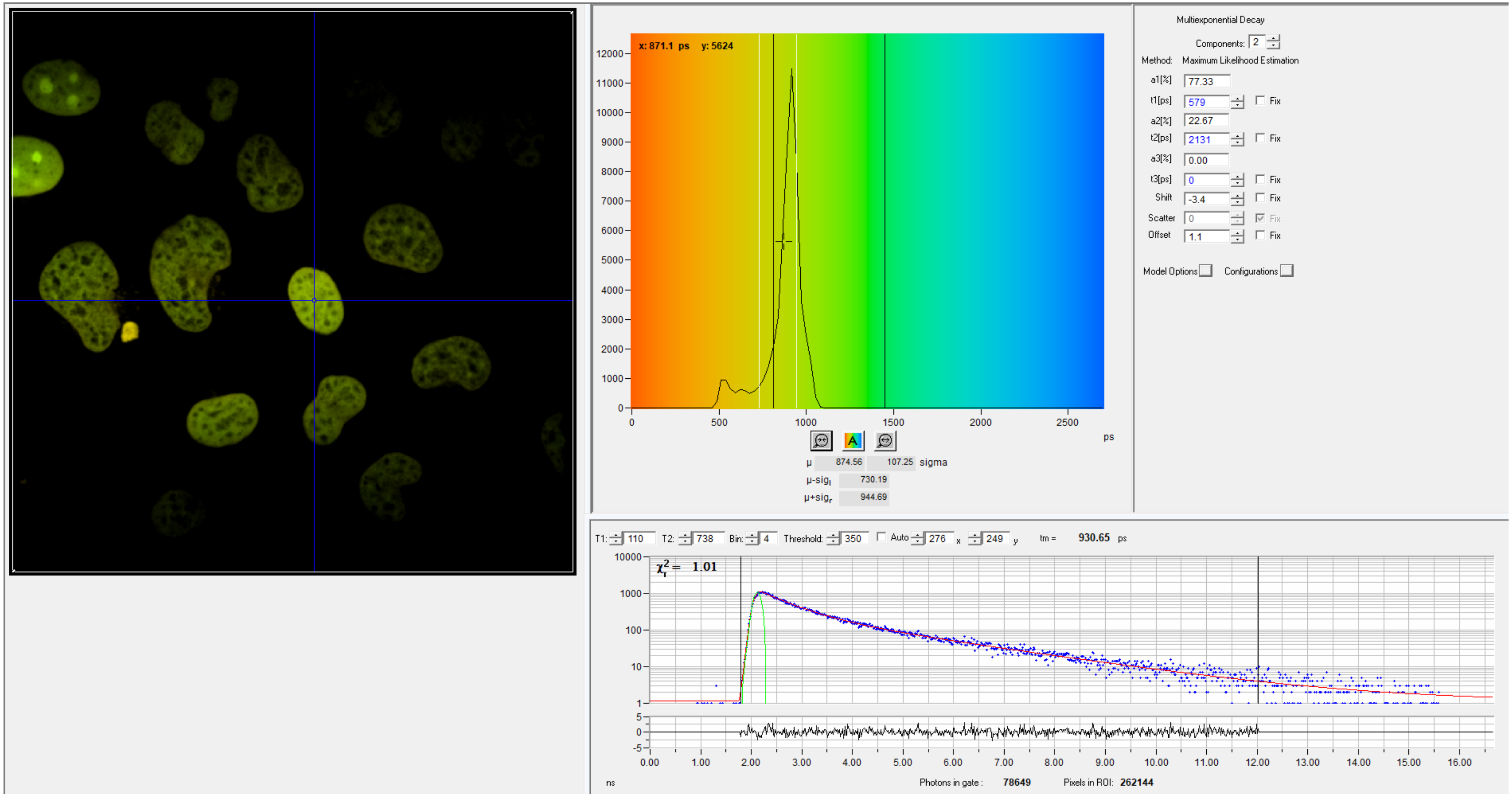

Figure S20. **H2B-FAST (original)** fuse in complex with **N871b**; bi-exponential fit; τ_m color-coding. FLIM scan and corresponding time-resolved fluorescence data analysis of life HeLa cells expressing the original FAST variant fused to histone-2B (H2B) and stained with **N871b**. A screenshot from Becker&Hickl SPCImage data acquisition and analysis window is shown. Bi-exponential fitting of decay data was performed. On the left panel is a FLIM image of HeLa nuclei, color-coded according to amplitude-weighted average fluorescence lifetime in each pixel (τ_i). A histogram on the upper right panel displays distribution of τ_m and color legend. The right panel represents bi-exponential fitting model used to fit data and fitting results. The lower data shows data on fluorescence decay. Blue dots represent experimental decay data, red line represents bi-exponential fit, green line represents instrument response function (IRF), fitting residuals shown as black graph below main data plot.

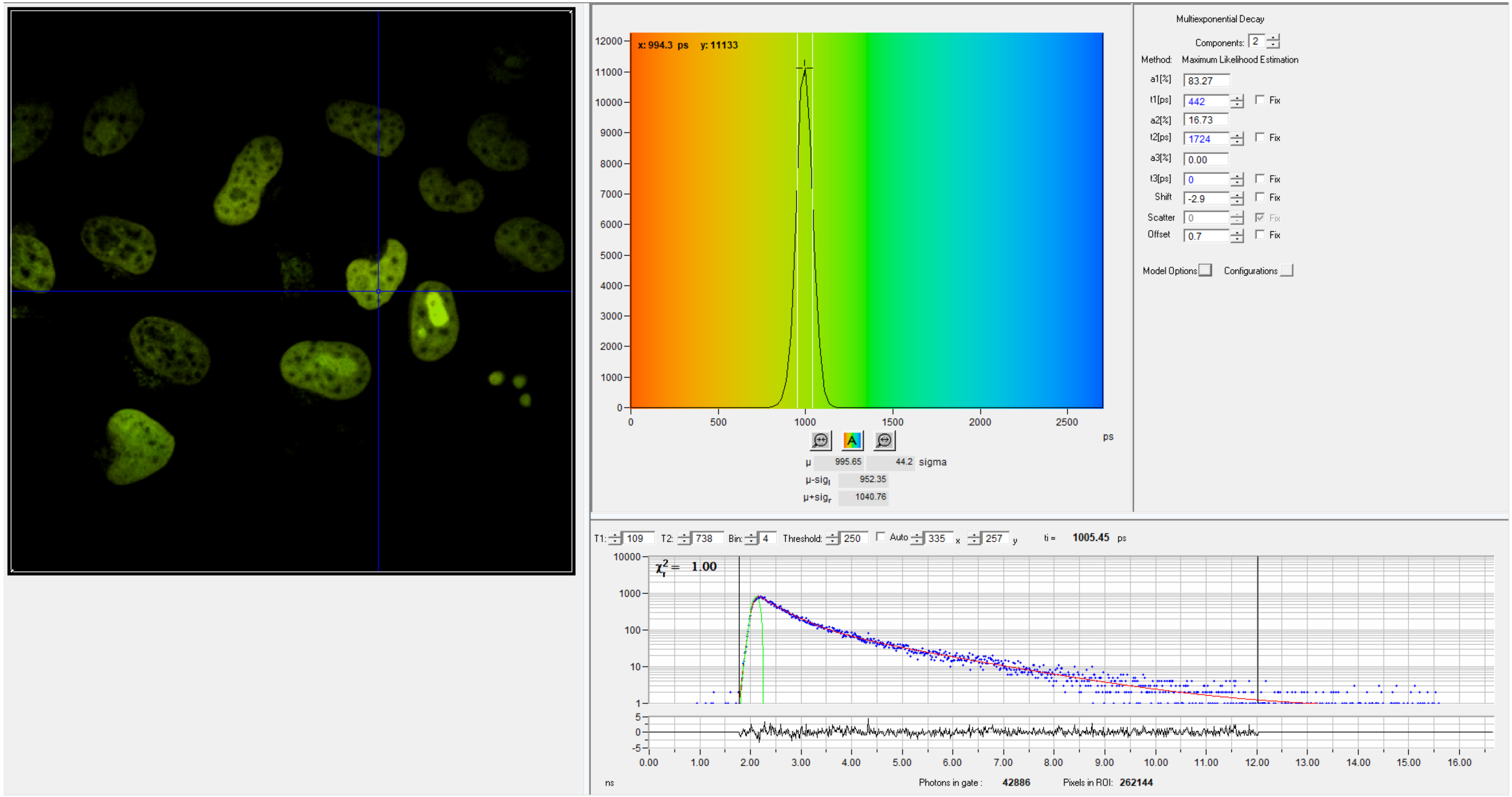

Figure S21. **H2B-F62L** fuse in complex with **N871b**; bi-exponential fit; τ_i color-coding. FLIM scan and corresponding time-resolved fluorescence data analysis of life HeLa cells expressing the F62L FAST variant fused to histone-2B (H2B) and stained with **N871b**. A screenshot from Becker&Hickl SPCImage data acquisition and analysis window is shown. Bi-exponential fitting of decay data was performed. On the left panel is a FLIM image of HeLa nuclei, color-coded according to intensity-weighted average fluorescence lifetime in each pixel (τ_i). A histogram on the upper right panel displays distribution of τ_i and color legend. The right panel represents bi-exponential fitting model used to fit data and fitting results. The lower data shows data on fluorescence decay. Blue dots represent experimental decay data, red line represents bi-exponential fit, green line represents instrument response function (IRF), fitting residuals shown as black graph below main data plot.

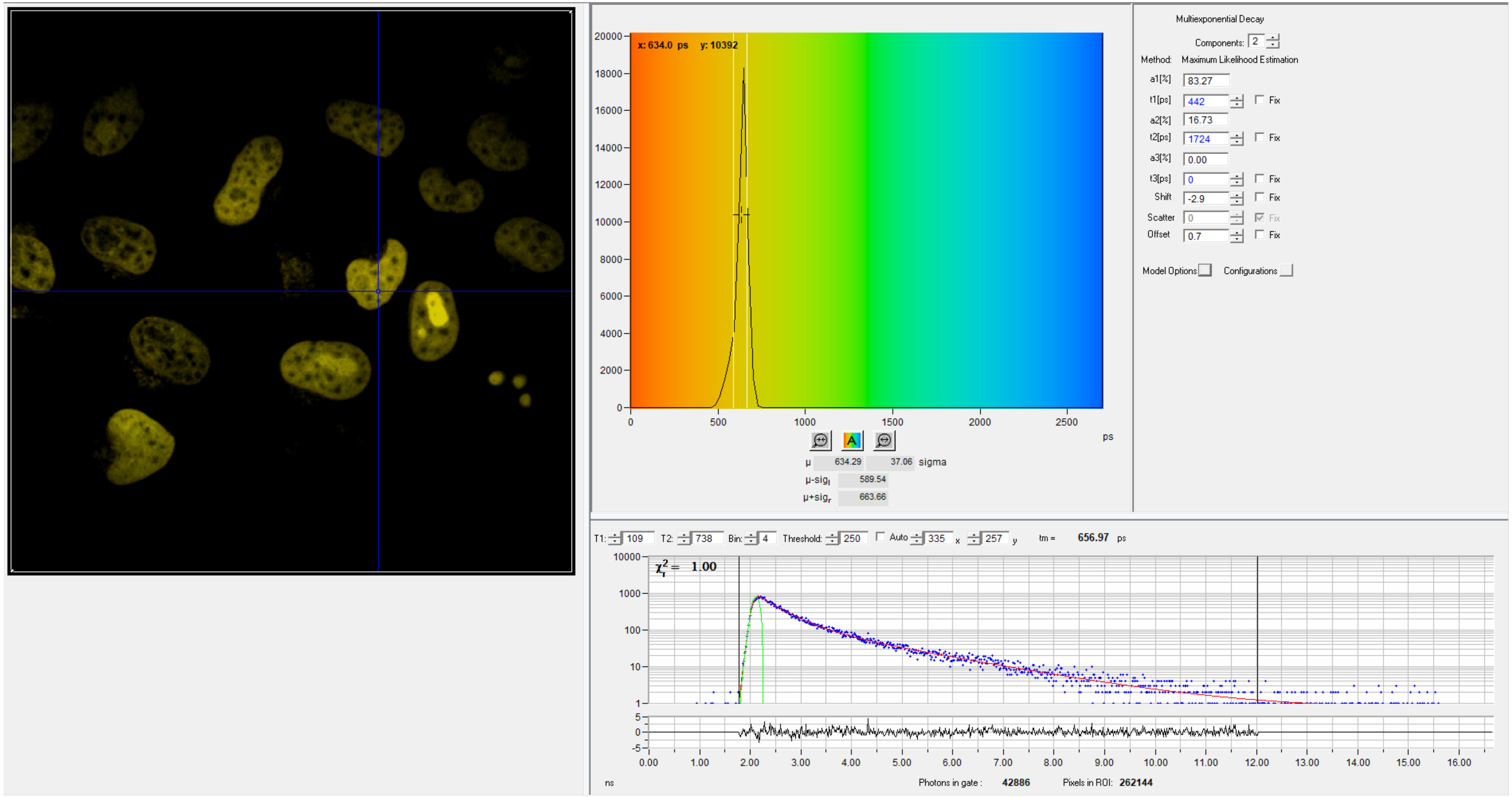

Figure S22. **H2B-F62L** fuse in complex with **N871b**; bi-exponential fit; τ_m color-coding. FLIM scan and corresponding time-resolved fluorescence data analysis of life HeLa cells expressing the F62L FAST variant fused to histone-2B (H2B) and stained with **N871b**. A screenshot from Becker&Hickl SPCImage data acquisition and analysis window is shown. Bi-exponential fitting of decay data was performed. On the left panel is a FLIM image of HeLa nuclei, color-coded according to amplitude-weighted average fluorescence lifetime in each pixel (τ_m). A histogram on the upper right panel displays distribution of τ_m and color legend. The right panel represents bi-exponential fitting model used to fit data and fitting results. The lower data shows data on fluorescence decay. Blue dots represent experimental decay data, red line represents bi-exponential fit, green line represents instrument response function (IRF), fitting residuals shown as black graph below main data plot.

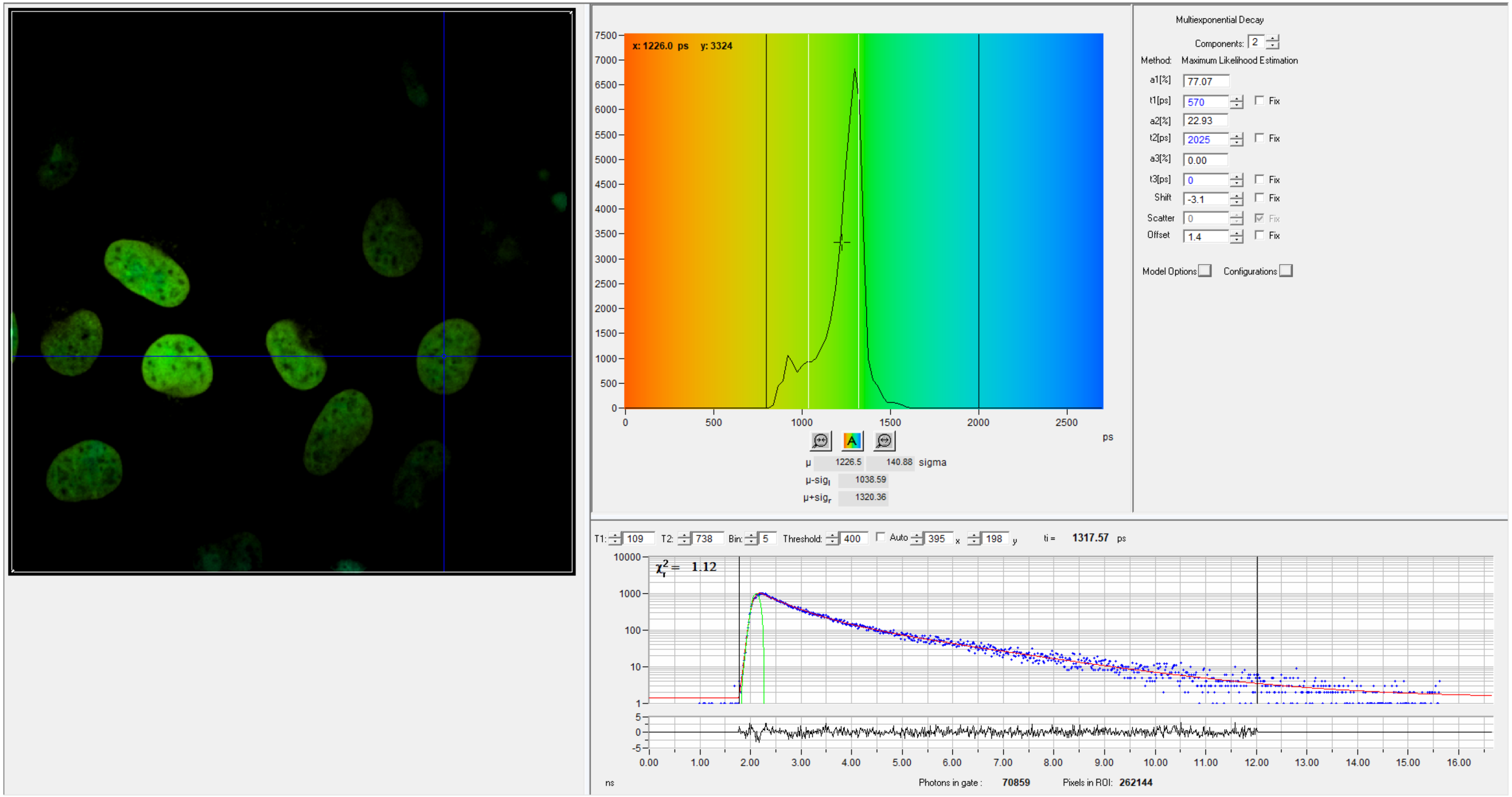

Figure S23. **H2B-D65K** fuse in complex with **N871b**; τ_i color-coding. FLIM scan and corresponding time-resolved fluorescence data analysis of life HeLa cells expressing the D65K FAST variant fused to histone-2B (H2B) and stained with **N871b**. A screenshot from Becker&Hickl SPCImage data acquisition and analysis window is shown. Bi-exponential fitting of decay data was performed. On the left panel is a FLIM image of HeLa nuclei, color-coded according to intensity-weighted average fluorescence lifetime in each pixel (τ_i). A histogram on the upper right panel displays distribution of τ_i and color legend. The right panel represents bi-exponential fitting model used to fit data and fitting results. The lower data shows data on fluorescence decay. Blue dots represent experimental decay data, red line represents bi-exponential fit, green line represents instrument response function (IRF), fitting residuals shown as black graph below main data plot.

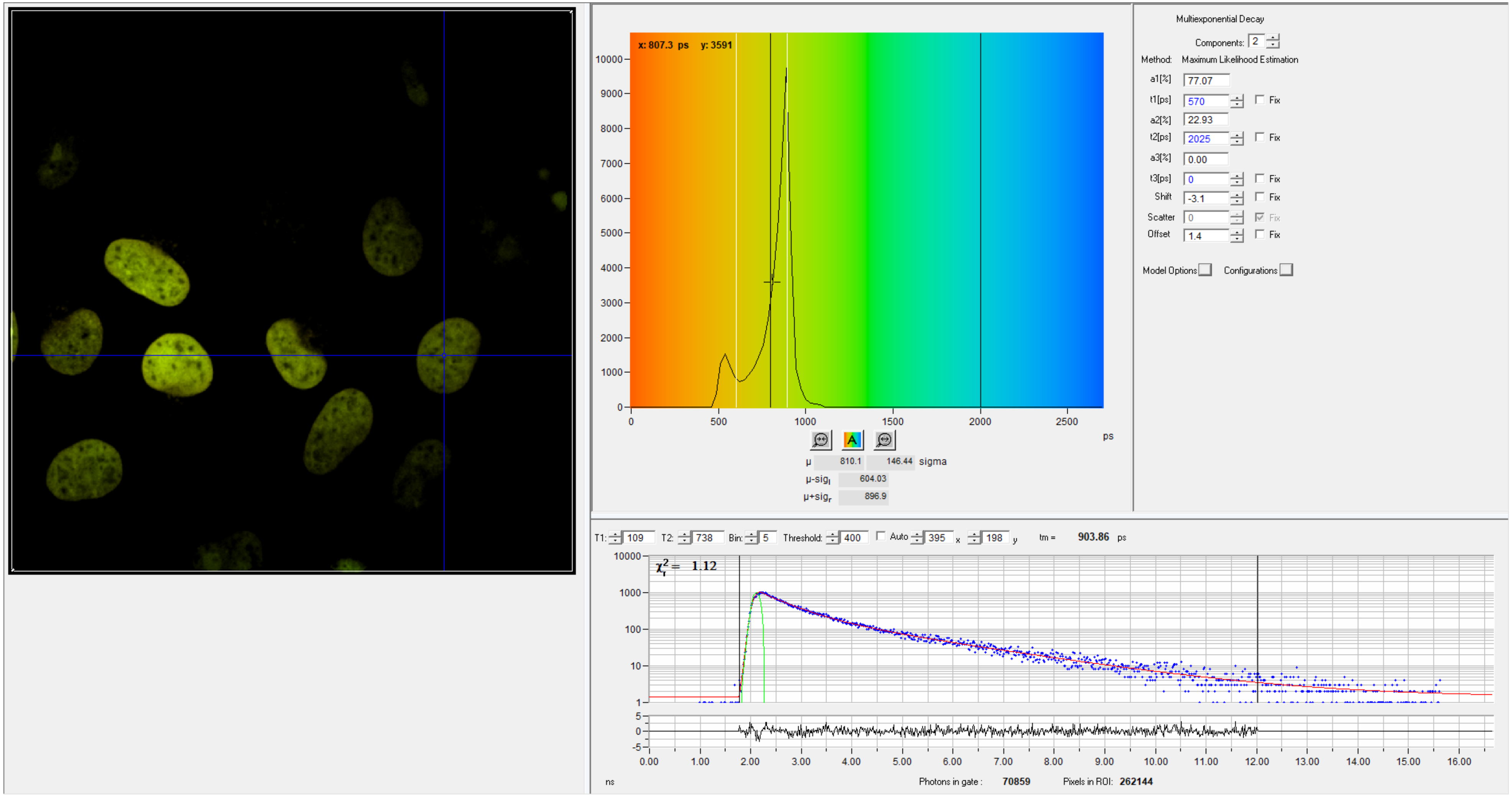

Figure S24. **H2B-D65K** fuse in complex with **N871b**; bi-exponential fit; τ_m color-coding. FLIM scan and corresponding time-resolved fluorescence data analysis of life HeLa cells expressing the D65K FAST variant fused to histone-2B (H2B) and stained with **N871b**. A screenshot from Becker&Hickl SPCImage data acquisition and analysis window is shown. Bi-exponential fitting of decay data was performed. On the left panel is a FLIM image of HeLa nuclei, color-coded according to amplitude-weighted average fluorescence lifetime in each pixel (τ_m). A histogram on the upper right panel displays distribution of τ_i and color legend. The right panel represents bi-exponential fitting model used to fit data and fitting results. The lower data shows data on fluorescence decay. Blue dots represent experimental decay data, red line represents bi-exponential fit, green line represents instrument response function (IRF), fitting residuals shown as black graph below main data plot.

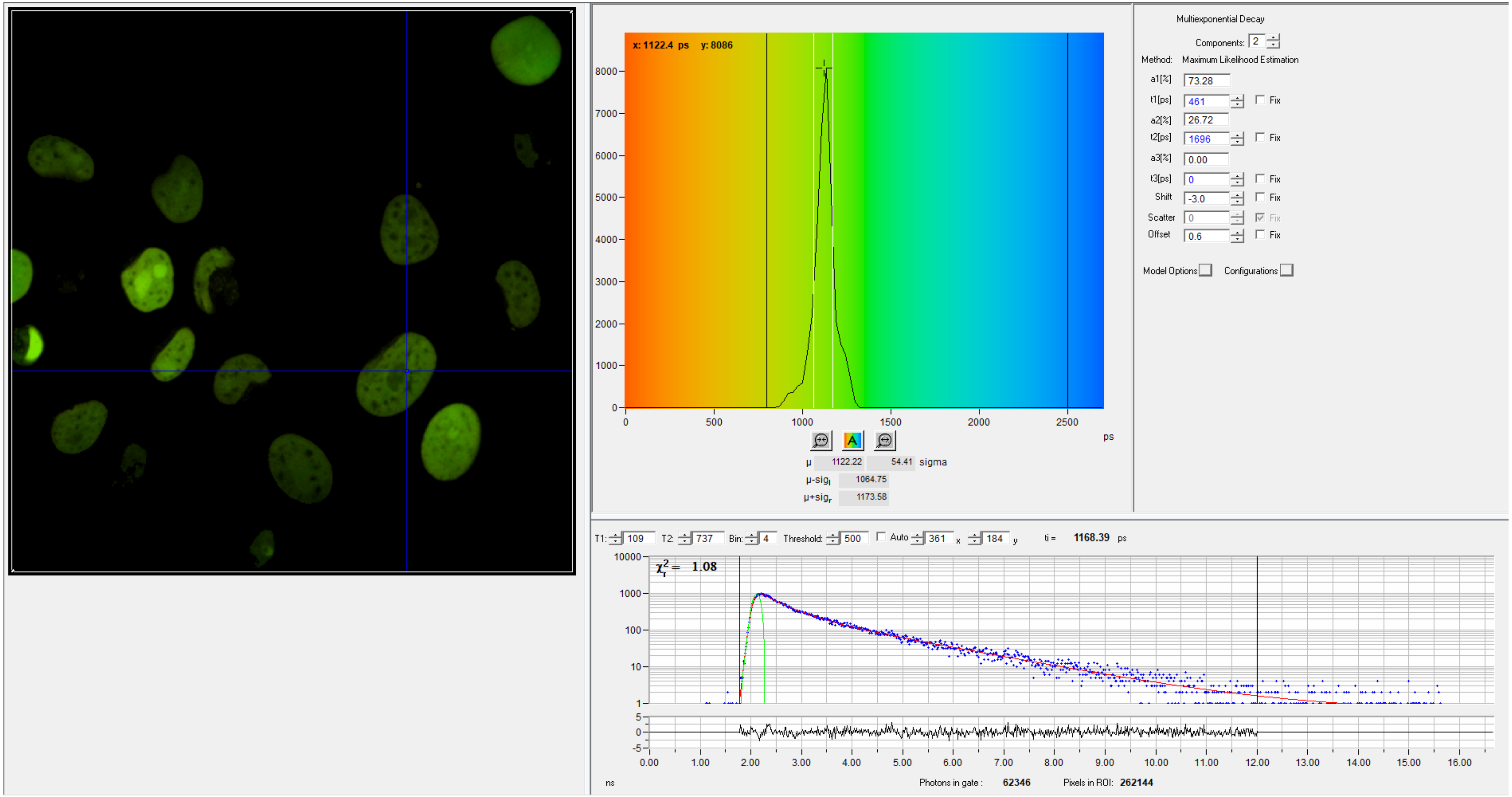

Figure S25. **H2B-P68K** fuse in complex with **N871b**; bi-exponential fit; τ_i color-coding. FLIM scan and corresponding time-resolved fluorescence data analysis of life HeLa cells expressing the P68K FAST variant fused to histone-2B (H2B) and stained with **N871b**. A screenshot from Becker&Hickl SPCImage data acquisition and analysis window is shown. Bi-exponential fitting of decay data was performed. On the left panel is a FLIM image of HeLa nuclei, color-coded according to intensity-weighted average fluorescence lifetime in each pixel (τ_i). A histogram on the upper right panel displays distribution of τ_i and color legend. The right panel represents bi-exponential fitting model used to fit data and fitting results. The lower data shows data on fluorescence decay. Blue dots represent experimental decay data, red line represents bi-exponential fit, green line represents instrument response function (IRF), fitting residuals shown as black graph below main data plot.

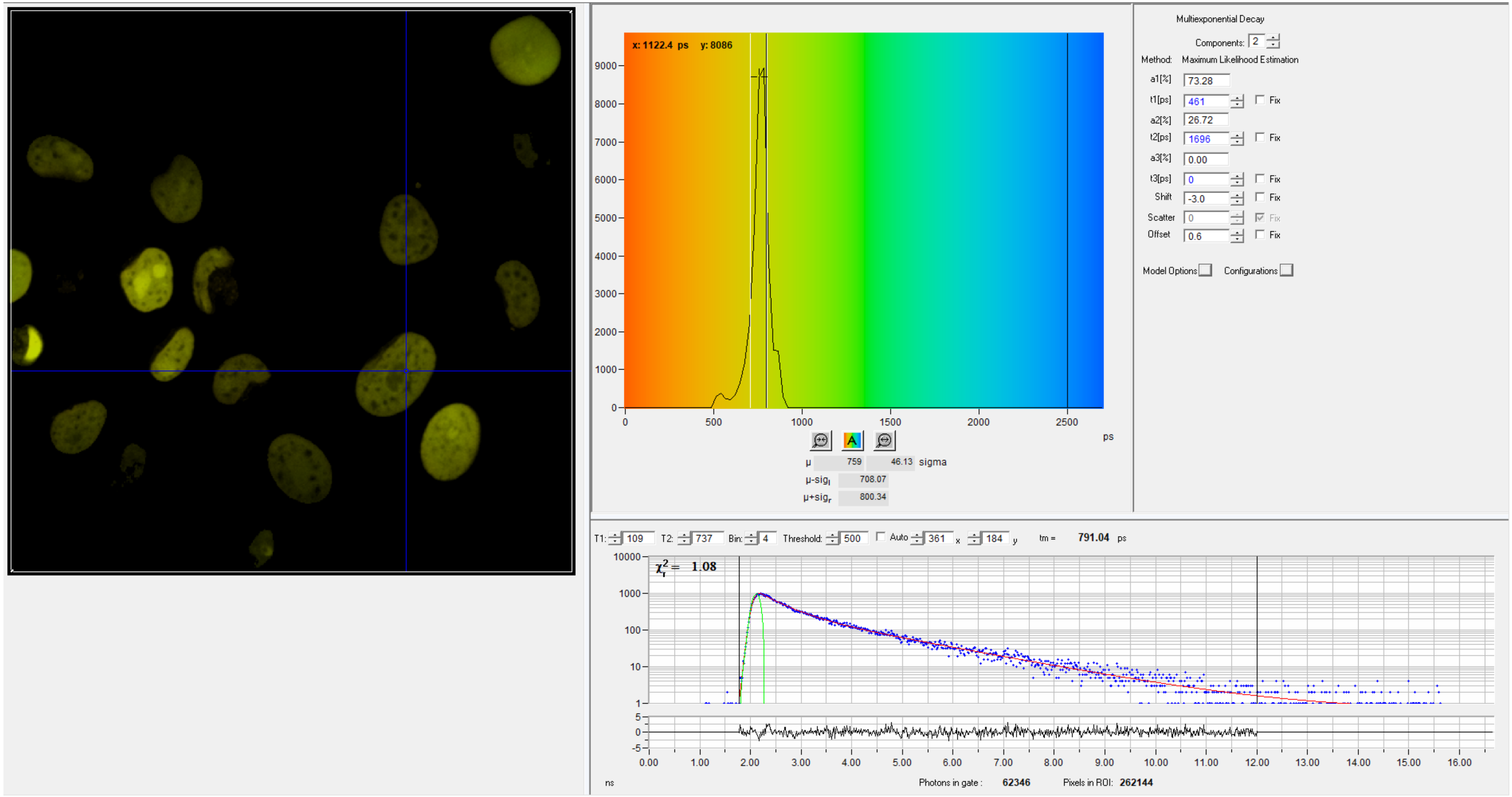

Figure S26. **H2B-P68K** fuse in complex with **N871b**; bi-exponential fit; τ_m color-coding. FLIM scan and corresponding time-resolved fluorescence data analysis of life HeLa cells expressing the P68K FAST variant fused to histone-2B (H2B) and stained with **N871b**. A screenshot from Becker&Hickl SPCImage data acquisition and analysis window is shown. Bi-exponential fitting of decay data was performed. On the left panel is a FLIM image of HeLa nuclei, color-coded according to amplitude-weighted average fluorescence lifetime in each pixel (τ_m). A histogram on the upper right panel displays distribution of τ_m and color legend. The right panel represents bi-exponential fitting model used to fit data and fitting results. The lower data shows data on fluorescence decay. Blue dots represent experimental decay data, red line represents bi-exponential fit, green line represents instrument response function (IRF), fitting residuals shown as black graph below main data plot.

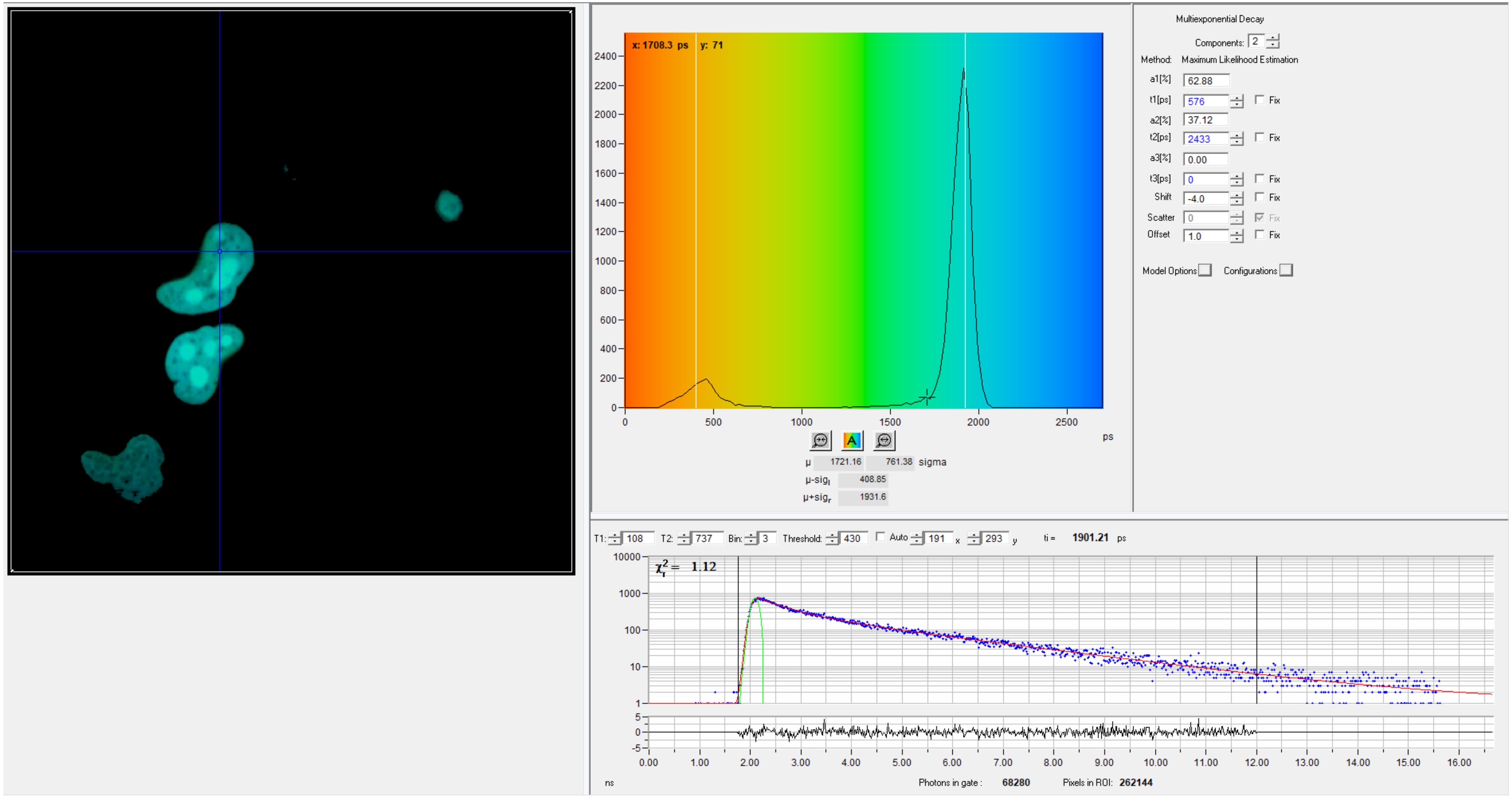

Figure S27. **H2B-R52Y** fuse in complex with **N871b**; bi-exponential fit; τ_i color-coding. FLIM scan and corresponding time-resolved fluorescence data analysis of life HeLa cells expressing the R52Y FAST variant fused to histone-2B (H2B) and stained with **N871b**. A screenshot from Becker&Hickl SPCImage data acquisition and analysis window is shown. Bi-exponential fitting of decay data was performed. On the left panel is a FLIM image of HeLa nuclei, color-coded according to intensity-weighted average fluorescence lifetime in each pixel (τ_i). A histogram on the upper right panel displays distribution of τ_i and color legend. The right panel represents bi-exponential fitting model used to fit data and fitting results. The lower data shows data on fluorescence decay. Blue dots represent experimental decay data, red line represents bi-exponential fit, green line represents instrument response function (IRF), fitting residuals shown as black graph below main data plot.

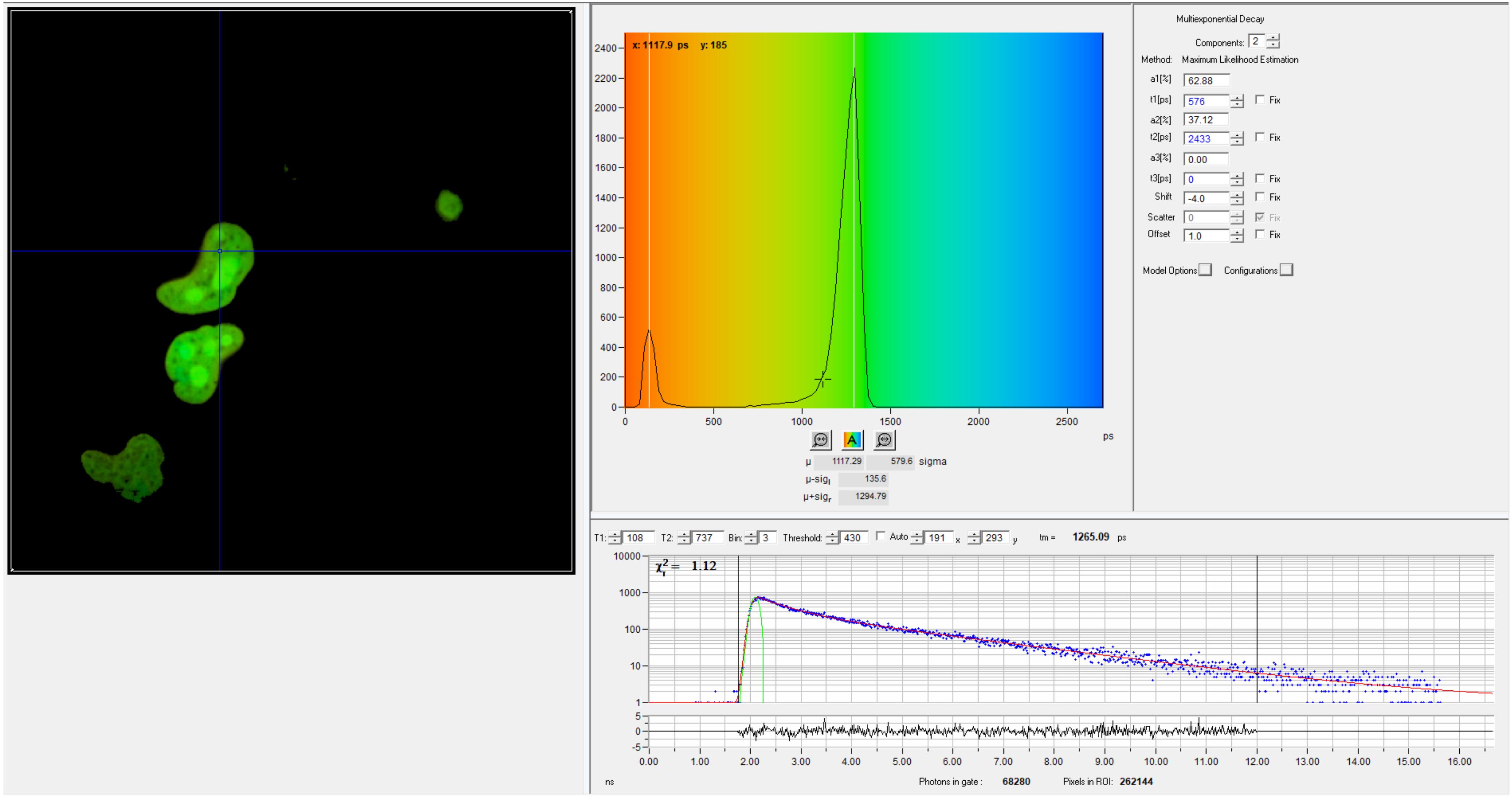

Figure S28. **H2B-R52Y** fuse in complex with **N871b**; bi-exponential fit; τ_m color-coding. FLIM scan and corresponding time-resolved fluorescence data analysis of life HeLa cells expressing the R52Y FAST variant fused to histone-2B (H2B) and stained with **N871b**. A screenshot from Becker&Hickl SPCImage data acquisition and analysis window is shown. Bi-exponential fitting of decay data was performed. On the left panel is a FLIM image of HeLa nuclei, color-coded according to amplitude-weighted average fluorescence lifetime in each pixel (τ_m). A histogram on the upper right panel displays distribution of τ_m and color legend. The right panel represents bi-exponential fitting model used to fit data and fitting results. The lower data shows data on fluorescence decay. Blue dots represent experimental decay data, red line represents bi-exponential fit, green line represents instrument response function (IRF), fitting residuals shown as black graph below main data plot.

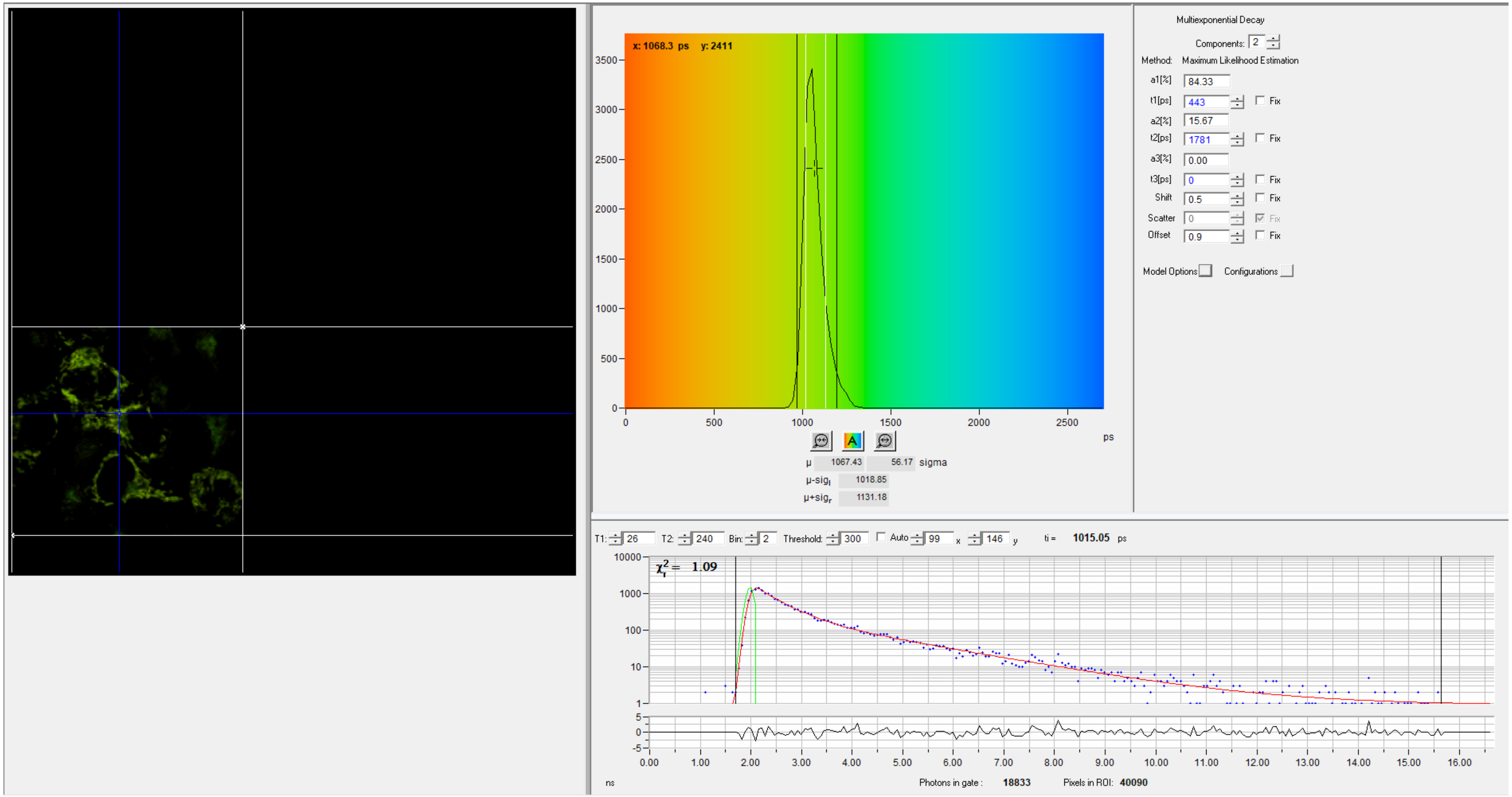

Figure S29. **IMS-F62L** fuse in complex with **N871b**; bi-exponential fit; τ_i color-coding. FLIM scan and corresponding time-resolved fluorescence data analysis of life HeLa cells expressing the F62L FAST variant fused to IMS and stained with **N871b**. A screenshot from Becker&Hickl SPCImage data acquisition and analysis window is shown. Bi-exponential fitting of decay data was performed. On the left panel is a FLIM image of HeLa nuclei, color-coded according to intensity-weighted average fluorescence lifetime in each pixel (τ_i). A histogram on the upper right panel displays distribution of τ_i and color legend. The right panel represents bi-exponential fitting model used to fit data and fitting results. The lower data shows data on fluorescence decay. Blue dots represent experimental decay data, red line represents bi-exponential fit, green line represents instrument response function (IRF), fitting residuals shown as black graph below main data plot.

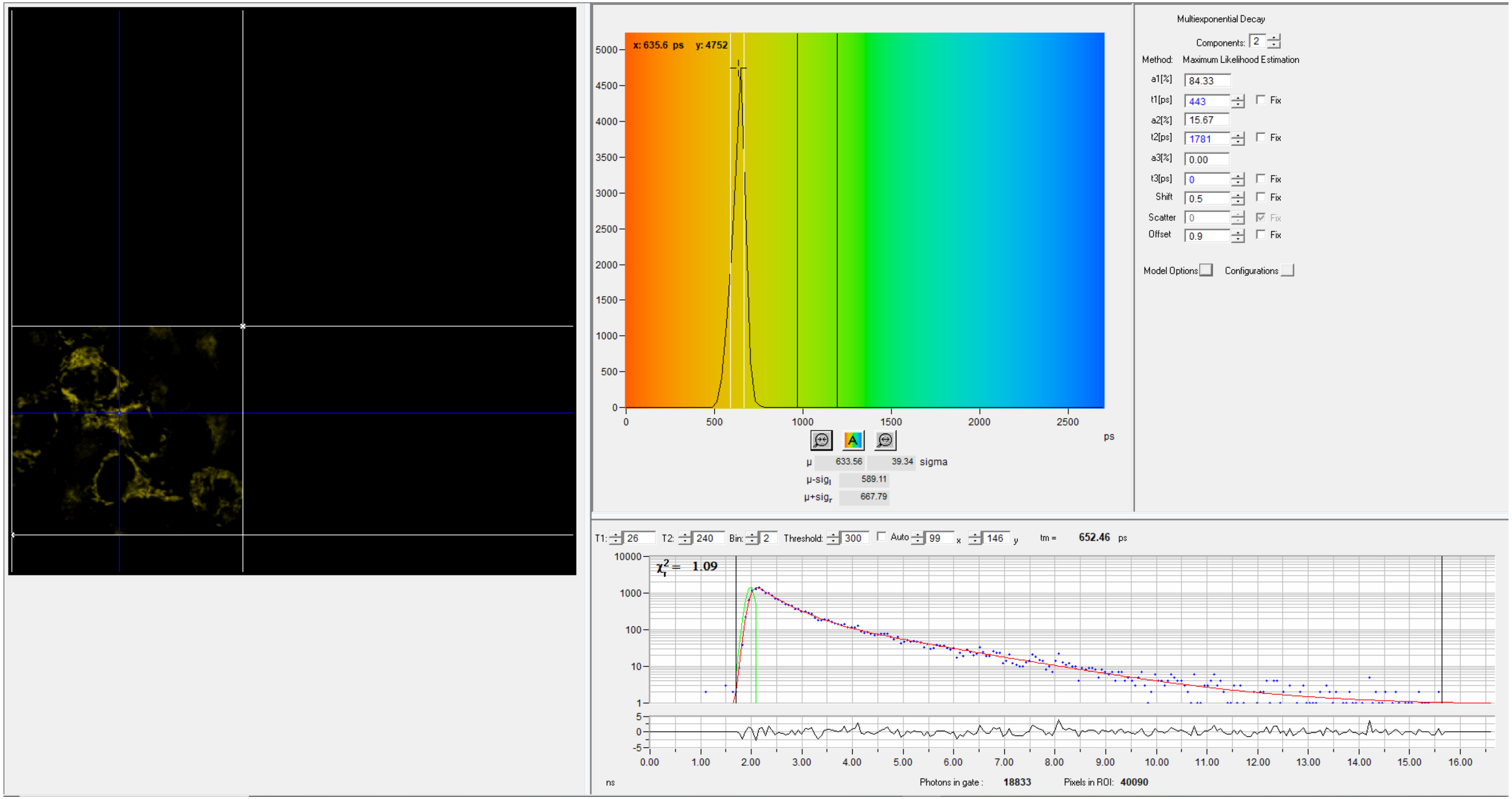

Figure S30. **IMS-F62L** fuse in complex with **N871b**; bi-exponential fit; τ_m color-coding. FLIM scan and corresponding time-resolved fluorescence data analysis of life HeLa cells expressing the F62L FAST variant fused to IMS and stained with **N871b**. A screenshot from Becker&Hickl SPCImage data acquisition and analysis window is shown. Bi-exponential fitting of decay data was performed. On the left panel is a FLIM image of HeLa nuclei, color-coded according to amplitude-weighted average fluorescence lifetime in each pixel (τ_m). A histogram on the upper right panel displays distribution of τ_m and color legend. The right panel represents bi-exponential fitting model used to fit data and fitting results. The lower data shows data on fluorescence decay. Blue dots represent experimental decay data, red line represents bi-exponential fit, green line represents instrument response function (IRF), fitting residuals shown as black graph below main data plot.

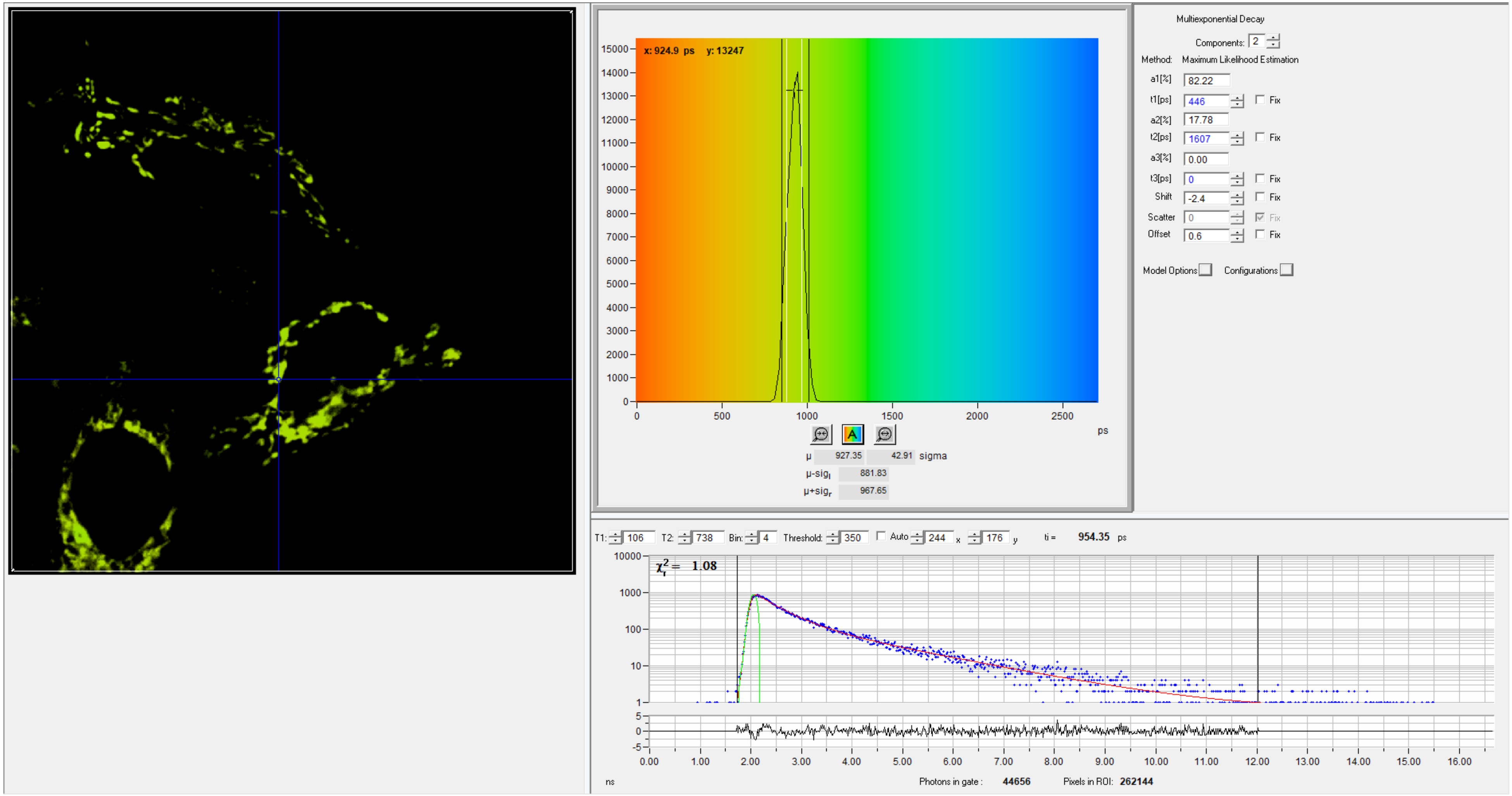

Figure S31. **IMS-P68K** fuse in complex with **N871b**; bi-exponential fit; τ_i color-coding. FLIM scan and corresponding time-resolved fluorescence data analysis of life HeLa cells expressing the P68K FAST variant fused to IMS and stained with **N871b**. A screenshot from Becker&Hickl SPCImage data acquisition and analysis window is shown. Bi-exponential fitting of decay data was performed. On the left panel is a FLIM image of HeLa nuclei, color-coded according to intensity-weighted average fluorescence lifetime in each pixel (τ_i). A histogram on the upper right panel displays distribution of τ_i and color legend. The right panel represents bi-exponential fitting model used to fit data and fitting results. The lower data shows data on fluorescence decay. Blue dots represent experimental decay data, red line represents bi-exponential fit, green line represents instrument response function (IRF), fitting residuals shown as black graph below main data plot.

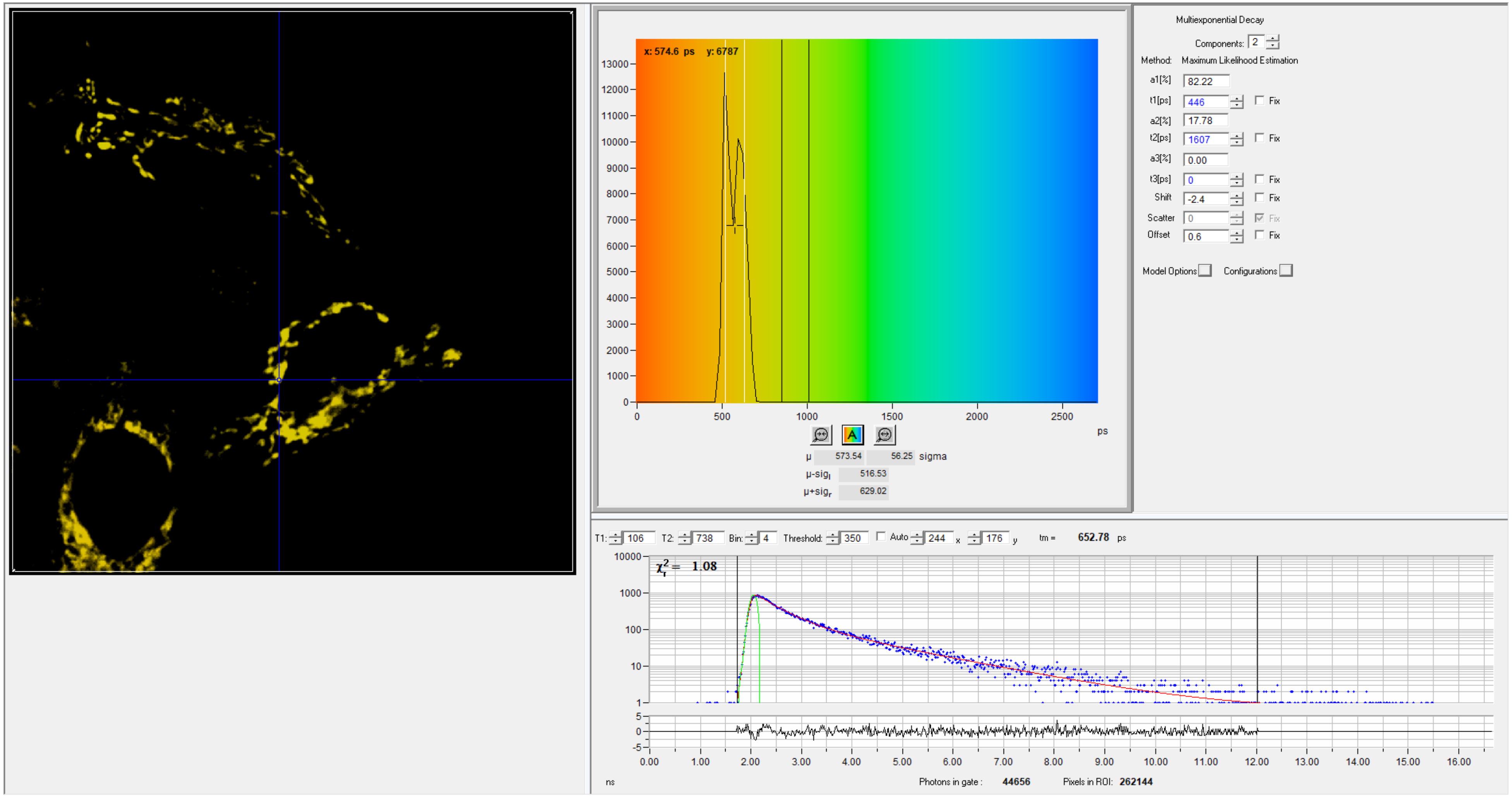

Figure S32. **IMS-P68K** fuse in complex with **N871b**; bi-exponential fit; τ_m color-coding. FLIM scan and corresponding time-resolved fluorescence data analysis of life HeLa cells expressing the P68K FAST variant fused to IMS and stained with **N871b**. A screenshot from Becker&Hickl SPCImage data acquisition and analysis window is shown. Bi-exponential fitting of decay data was performed. On the left panel is a FLIM image of HeLa nuclei, color-coded according to amplitude-weighted average fluorescence lifetime in each pixel (τ_m). A histogram on the upper right panel displays distribution of τ_m and color legend. The right panel represents bi-exponential fitting model used to fit data and fitting results. The lower data shows data on fluorescence decay. Blue dots represent experimental decay data, red line represents bi-exponential fit, green line represents instrument response function (IRF), fitting residuals shown as black graph below main data plot.

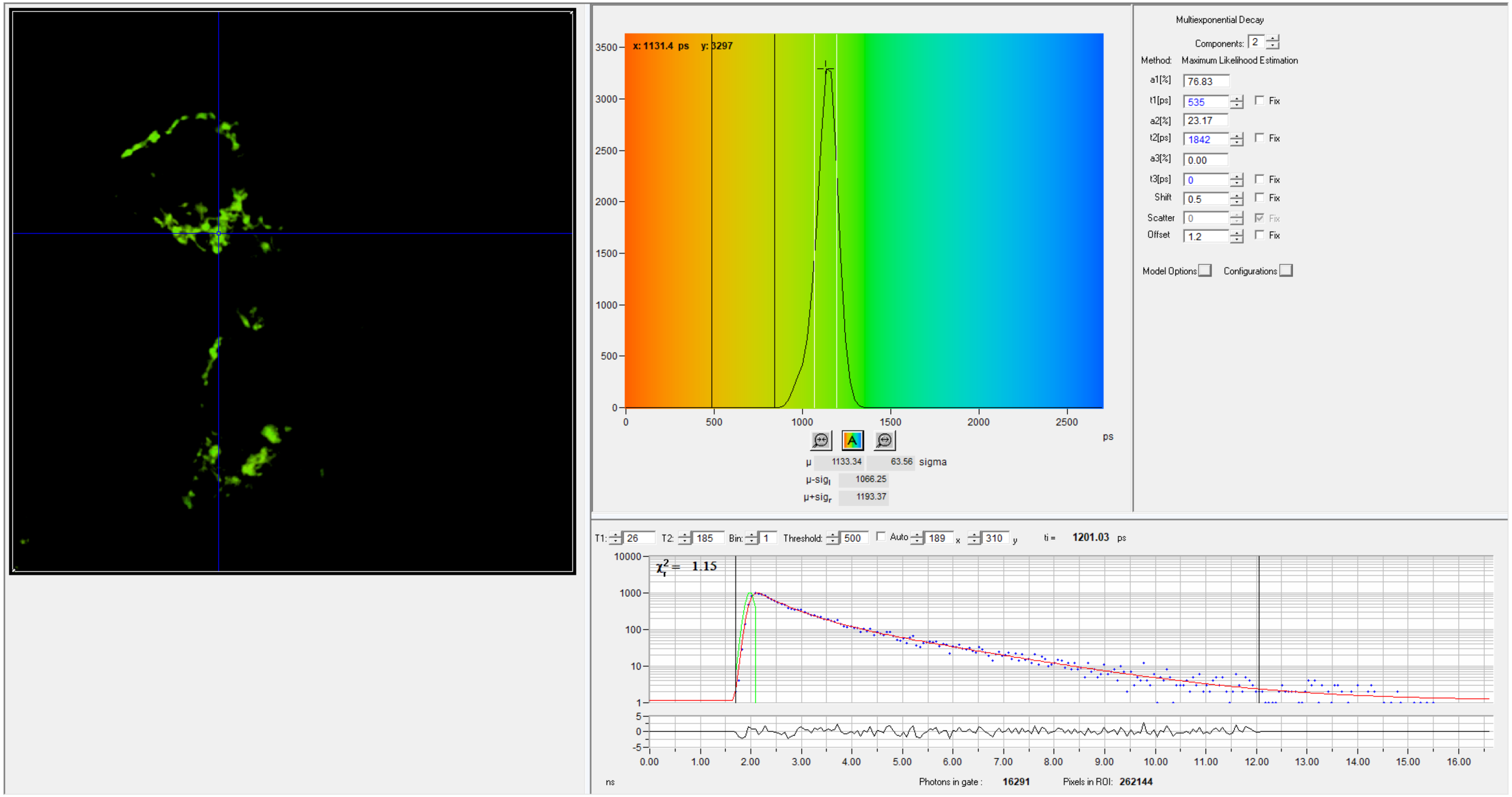

Figure S33. **IMS-D65K** fuse in complex with **N871b**; bi-exponential fit; τ_i color-coding. FLIM scan and corresponding time-resolved fluorescence data analysis of life HeLa cells expressing the D65K FAST variant fused to IMS and stained with **N871b**. A screenshot from Becker&Hickl SPCImage data acquisition and analysis window is shown. Bi-exponential fitting of decay data was performed. On the left panel is a FLIM image of HeLa nuclei, color-coded according to intensity-weighted average fluorescence lifetime in each pixel (τ_i). A histogram on the upper right panel displays distribution of τ_i and color legend. The right panel represents bi-exponential fitting model used to fit data and fitting results. The lower data shows data on fluorescence decay. Blue dots represent experimental decay data, red line represents bi-exponential fit, green line represents instrument response function (IRF), fitting residuals shown as black graph below main data plot.

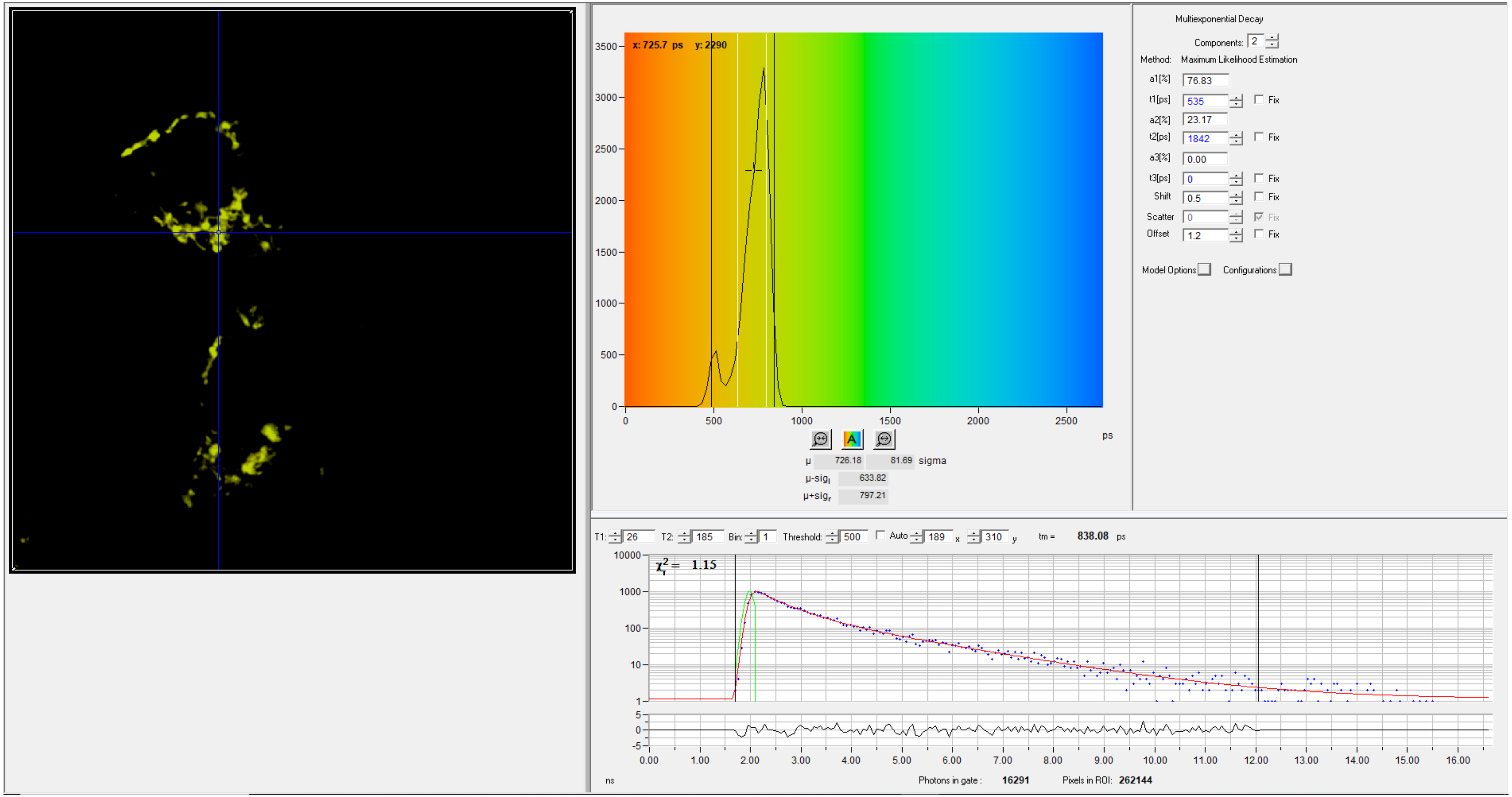

Figure S34. **IMS-D65K** fuse in complex with **N871b**; bi-exponential fit; τ_m color-coding. FLIM scan and corresponding time-resolved fluorescence data analysis of life HeLa cells expressing the D65K FAST variant fused to IMS and stained with **N871b**. A screenshot from Becker&Hickl SPCImage data acquisition and analysis window is shown. Bi-exponential fitting of decay data was performed. On the left panel is a FLIM image of HeLa nuclei, color-coded according to amplitude-weighted average fluorescence lifetime in each pixel (τ_m). A histogram on the upper right panel displays distribution of τ_m and color legend. The right panel represents bi-exponential fitting model used to fit data and fitting results. The lower data shows data on fluorescence decay. Blue dots represent experimental decay data, red line represents bi-exponential fit, green line represents instrument response function (IRF), fitting residuals shown as black graph below main data plot.

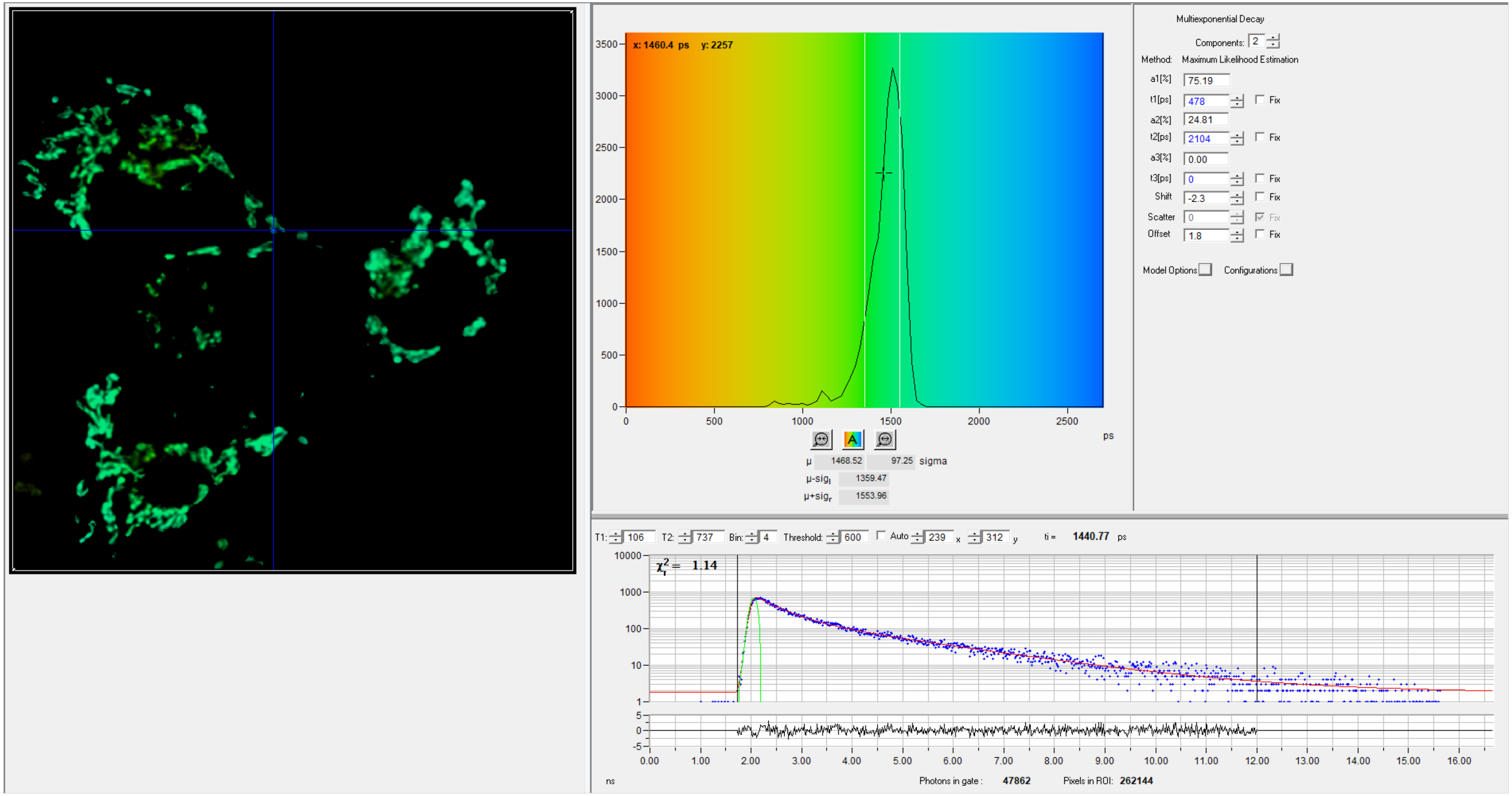

Figure S35. **IMS-R52Y** fuse in complex with **N871b**; bi-exponential fit; τ_i color-coding. FLIM scan and corresponding time-resolved fluorescence data analysis of life HeLa cells expressing the R52Y FAST variant fused to IMS and stained with N871b. A screenshot from Becker&Hickl SPCImage data acquisition and analysis window is shown. Bi-exponential fitting of decay data was performed. On the left panel is a FLIM image of HeLa nuclei, color-coded according to intensity-weighted average fluorescence lifetime in each pixel (τ_i). A histogram on the upper right panel displays distribution of τ_i and color legend. The right panel represents bi-exponential fitting model used to fit data and fitting results. The lower data shows data on fluorescence decay. Blue dots represent experimental decay data, red line represents bi-exponential fit, green line represents instrument response function (IRF), fitting residuals shown as black graph below main data plot.

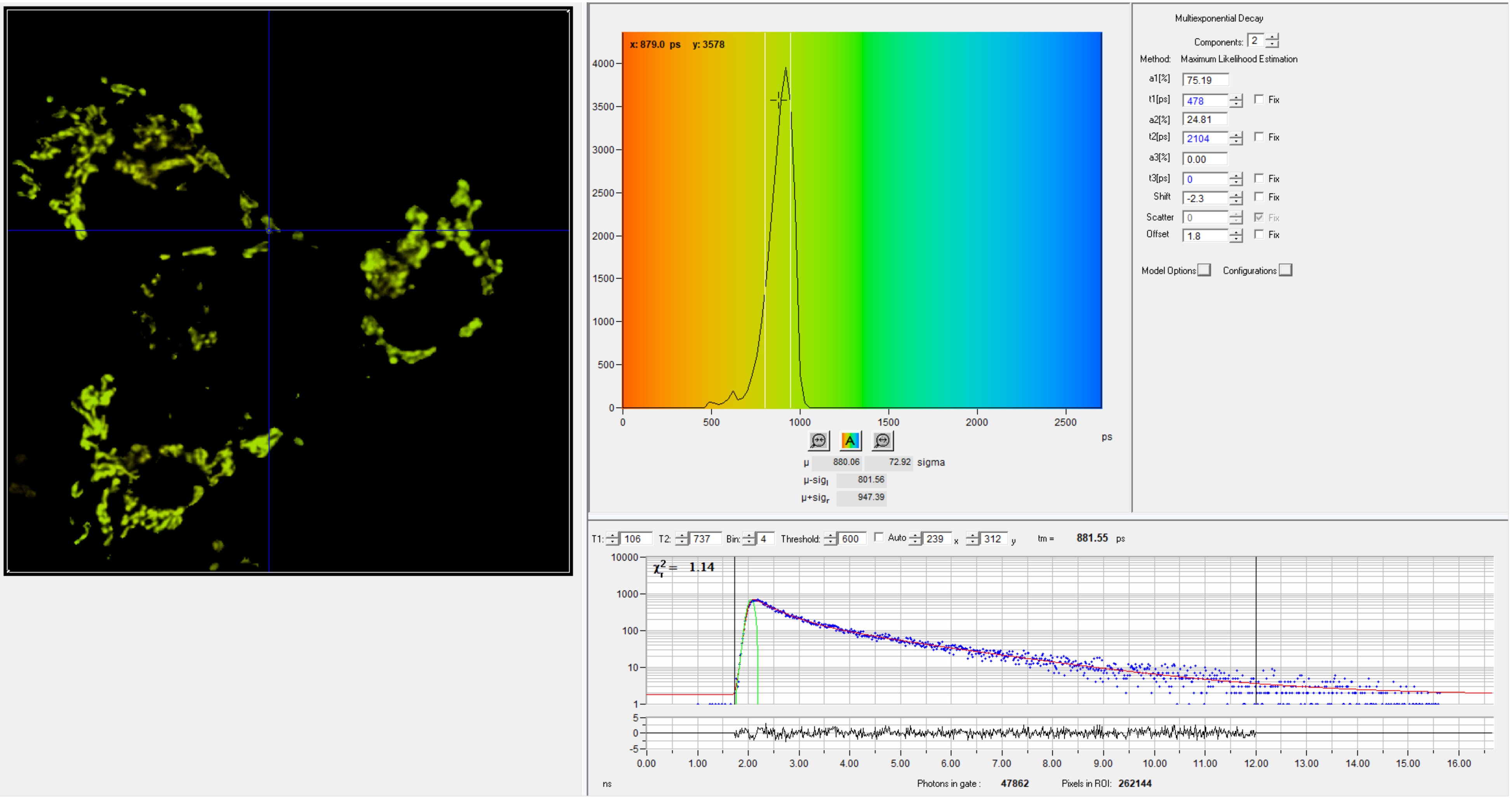

Figure S36. **IMS-R52Y** fuse in complex with **N871b**; bi-exponential fit; τ_m color-coding. FLIM scan and corresponding time-resolved fluorescence data analysis of life HeLa cells expressing the R52Y FAST variant fused to IMS and stained with **N871b**. A screenshot from Becker&Hickl SPCImage data acquisition and analysis window is shown. Bi-exponential fitting of decay data was performed. On the left panel is a FLIM image of HeLa nuclei, color-coded according to amplitude-weighted average fluorescence lifetime in each pixel (τ_m). A histogram on the upper right panel displays distribution of τ_m and color legend. The right panel represents bi-exponential fitting model used to fit data and fitting results. The lower data shows data on fluorescence decay. Blue dots represent experimental decay data, red line represents bi-exponential fit, green line represents instrument response function (IRF), fitting residuals shown as black graph below main data plot.

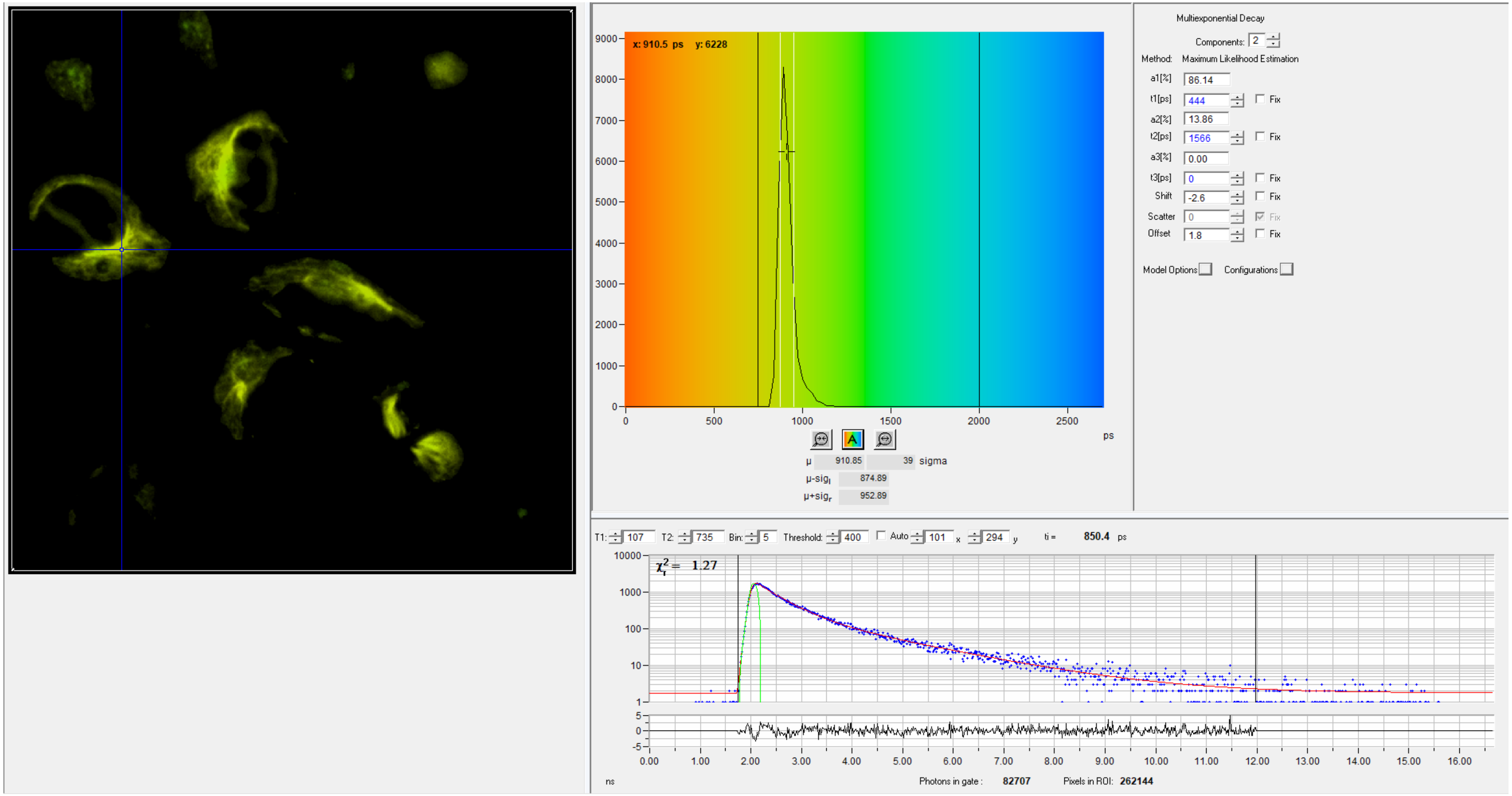

Figure S37. **Vimentin-F62L** fuse in complex with **N871b**; bi-exponential fit; τ_i color-coding. FLIM scan and corresponding time-resolved fluorescence data analysis of life HeLa cells expressing the F62L FAST variant fused to vimentin and stained with **N871b**. A screenshot from Becker&Hickl SPCImage data acquisition and analysis window is shown. Bi-exponential fitting of decay data was performed. On the left panel is a FLIM image of HeLa nuclei, color-coded according to intensity-weighted average fluorescence lifetime in each pixel (τ_i). A histogram on the upper right panel displays distribution of τ_i and color legend. The right panel represents bi-exponential fitting model used to fit data and fitting results. The lower data shows data on fluorescence decay. Blue dots represent experimental decay data, red line represents bi-exponential fit, green line represents instrument response function (IRF), fitting residuals shown as black graph below main data plot.

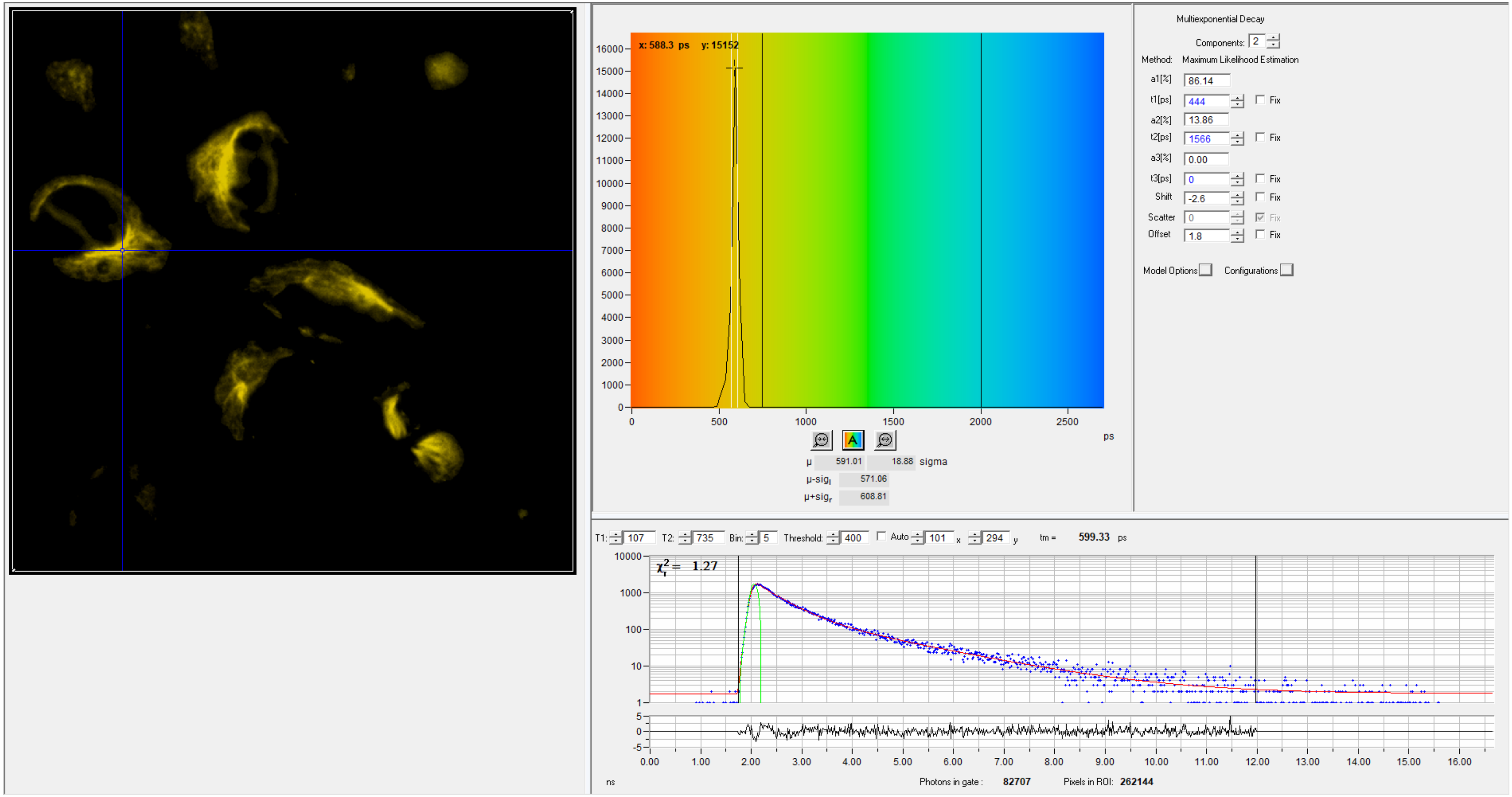

Figure S38. **Vimentin-F62L** fuse in complex with **N871b**; bi-exponential fit; τ_m color-coding. FLIM scan and corresponding time-resolved fluorescence data analysis of life HeLa cells expressing the F62L FAST variant fused to vimentin and stained with **N871b**. A screenshot from Becker&Hickl SPCImage data acquisition and analysis window is shown. Bi-exponential fitting of decay data was performed. On the left panel is a FLIM image of HeLa nuclei, color-coded according to amplitude-weighted average fluorescence lifetime in each pixel (τ_m). A histogram on the upper right panel displays distribution of τ_m and color legend. The right panel represents bi-exponential fitting model used to fit data and fitting results. The lower data shows data on fluorescence decay. Blue dots represent experimental decay data, red line represents bi-exponential fit, green line represents instrument response function (IRF), fitting residuals shown as black graph below main data plot.

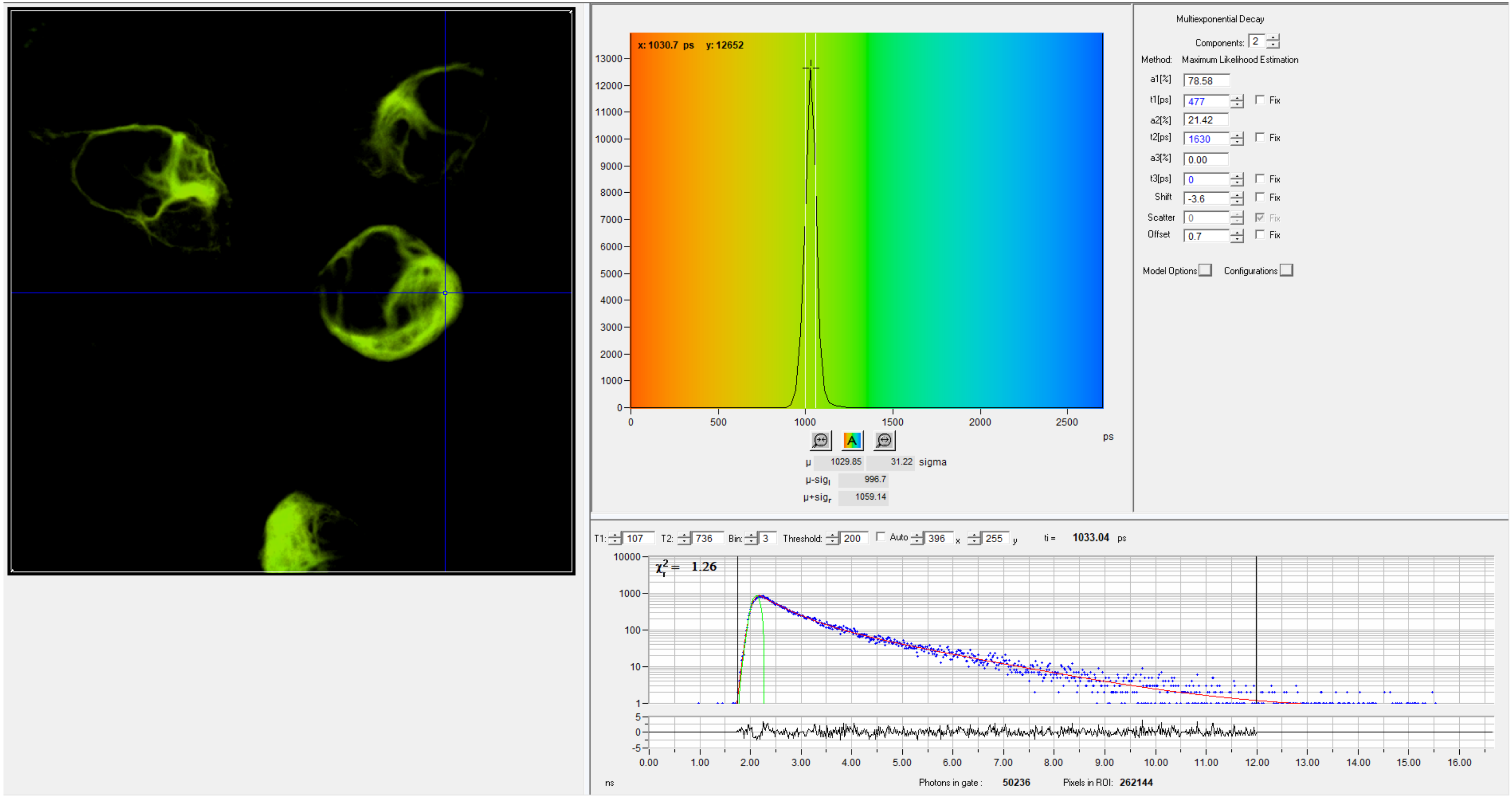

Figure S39. **Vimentin-P68K** fuse in complex with **N871b**; bi-exponential fit; τ_i color-coding. FLIM scan and corresponding time-resolved fluorescence data analysis of life HeLa cells expressing the P68K FAST variant fused to vimentin and stained with **N871b**. A screenshot from Becker&Hickl SPCImage data acquisition and analysis window is shown. Bi-exponential fitting of decay data was performed. On the left panel is a FLIM image of HeLa nuclei, color-coded according to intensity-weighted average fluorescence lifetime in each pixel (τ_i). A histogram on the upper right panel displays distribution of τ_i and color legend. The right panel represents bi-exponential fitting model used to fit data and fitting results. The lower data shows data on fluorescence decay. Blue dots represent experimental decay data, red line represents bi-exponential fit, green line represents instrument response function (IRF), fitting residuals shown as black graph below main data plot.

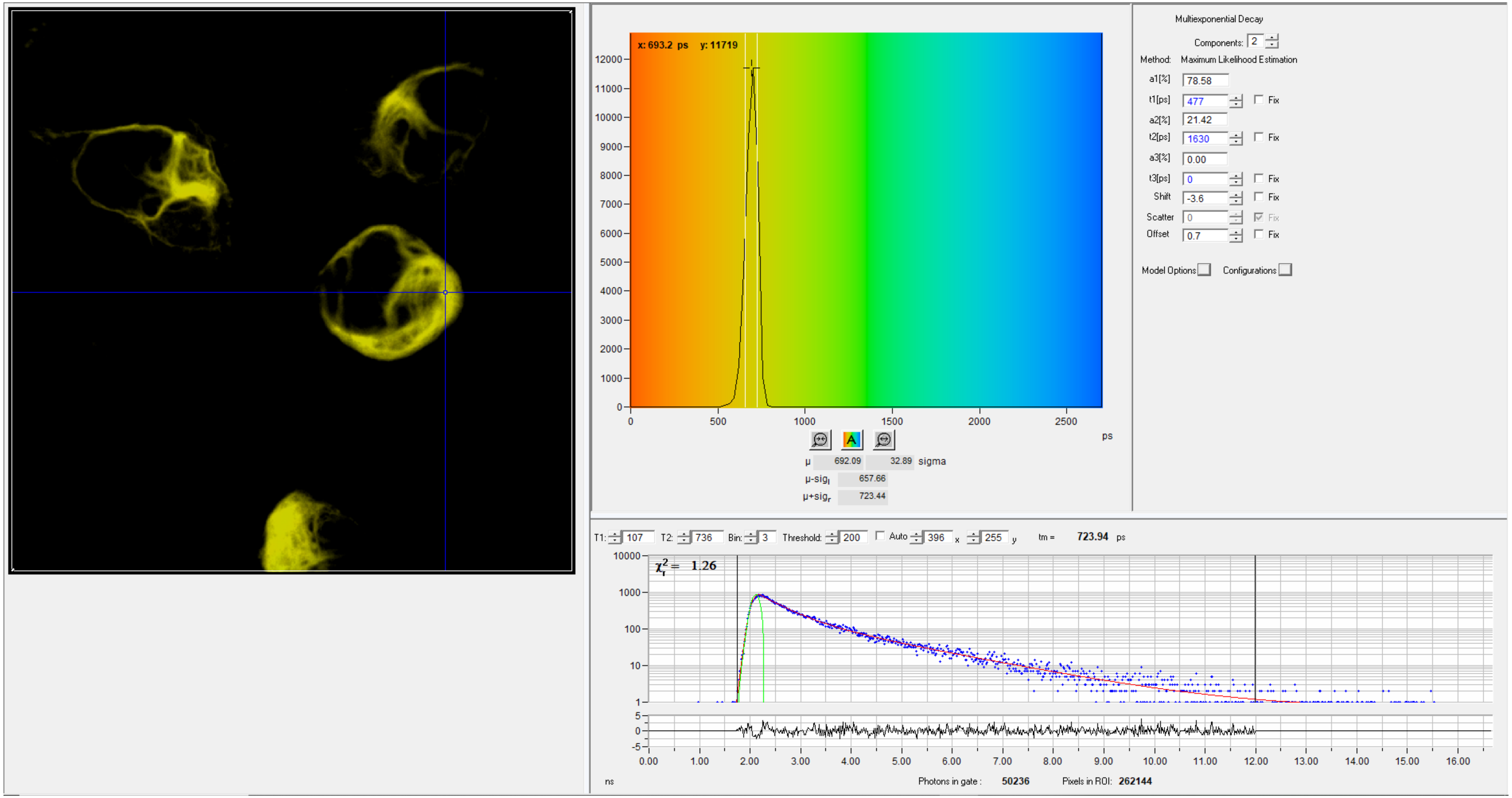

Figure S40. **Vimentin-P68K** fuse in complex with **N871b**; bi-exponential fit; τ_m color-coding. FLIM scan and corresponding time-resolved fluorescence data analysis of life HeLa cells expressing the P68K FAST variant fused to vimentin and stained with **N871b**. A screenshot from Becker&Hickl SPCImage data acquisition and analysis window is shown. Bi-exponential fitting of decay data was performed. On the left panel is a FLIM image of HeLa nuclei, color-coded according to amplitude-weighted average fluorescence lifetime in each pixel (τ_m). A histogram on the upper right panel displays distribution of τ_m and color legend. The right panel represents bi-exponential fitting model used to fit data and fitting results. The lower data shows data on fluorescence decay. Blue dots represent experimental decay data, red line represents bi-exponential fit, green line represents instrument response function (IRF), fitting residuals shown as black graph below main data plot.

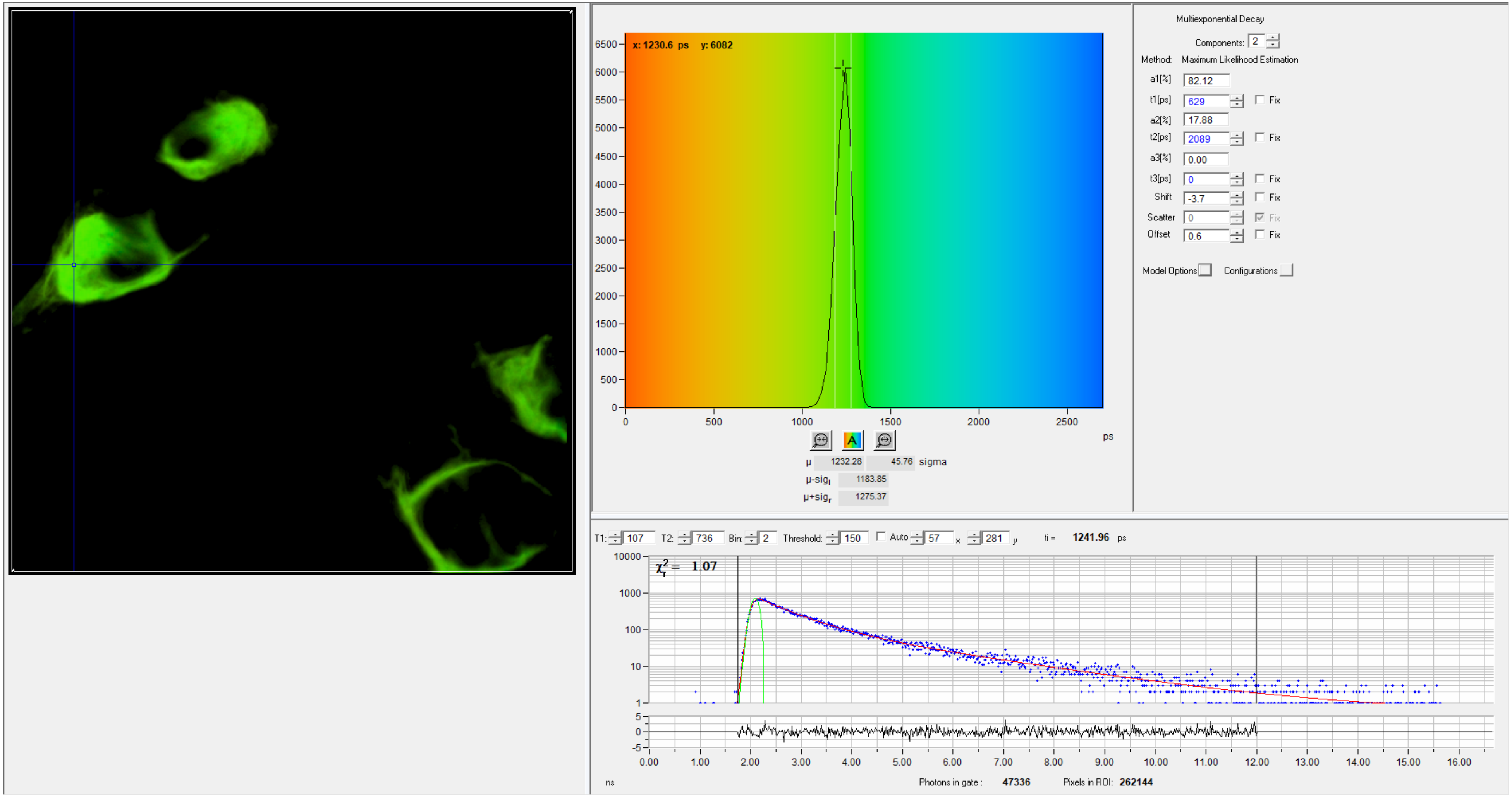

Figure S41. **Vimentin-D65K** fuse in complex with **N871b**; bi-exponential fit; τ_i color-coding. FLIM scan and corresponding time-resolved fluorescence data analysis of life HeLa cells expressing the D65K FAST variant fused to vimentin and stained with **N871b**. A screenshot from Becker&Hickl SPCImage data acquisition and analysis window is shown. Bi-exponential fitting of decay data was performed. On the left panel is a FLIM image of HeLa nuclei, color-coded according to intensity-weighted average fluorescence lifetime in each pixel (τ_i). A histogram on the upper right panel displays distribution of τ_i and color legend. The right panel represents bi-exponential fitting model used to fit data and fitting results. The lower data shows data on fluorescence decay. Blue dots represent experimental decay data, red line represents bi-exponential fit, green line represents instrument response function (IRF), fitting residuals shown as black graph below main data plot.

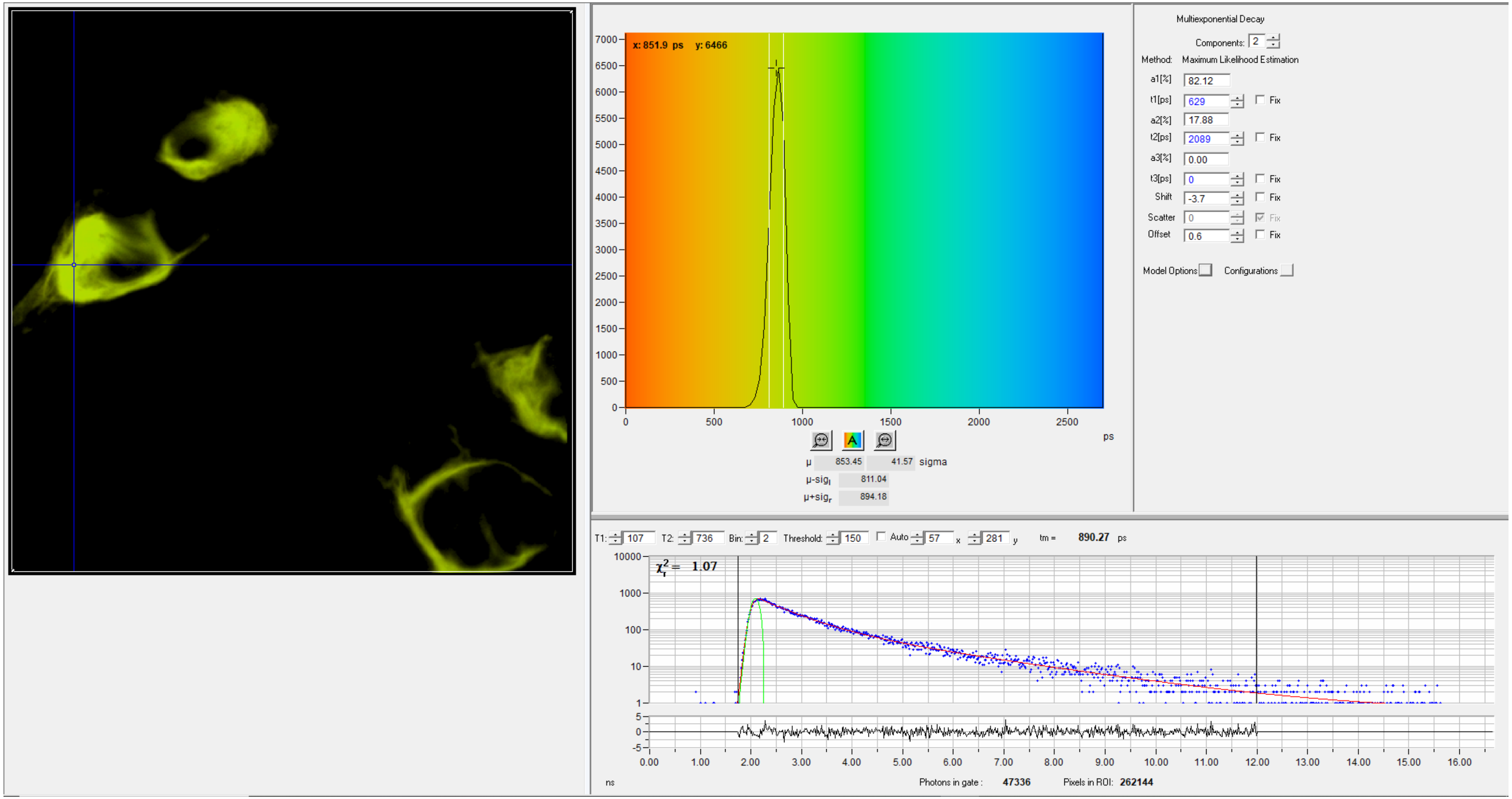

Figure S42. **Vimentin-D65K** fuse in complex with **N871b**; bi-exponential fit; τ_m color-coding. FLIM scan and corresponding time-resolved fluorescence data analysis of life HeLa cells expressing the D65K FAST variant fused to vimentin and stained with **N871b**. A screenshot from Becker&Hickl SPCImage data acquisition and analysis window is shown. Bi-exponential fitting of decay data was performed. On the left panel is a FLIM image of HeLa nuclei, color-coded according to amplitude-weighted average fluorescence lifetime in each pixel (τ_m). A histogram on the upper right panel displays distribution of τ_m and color legend. The right panel represents bi-exponential fitting model used to fit data and fitting results. The lower data shows data on fluorescence decay. Blue dots represent experimental decay data, red line represents bi-exponential fit, green line represents instrument response function (IRF), fitting residuals shown as black graph below main data plot.

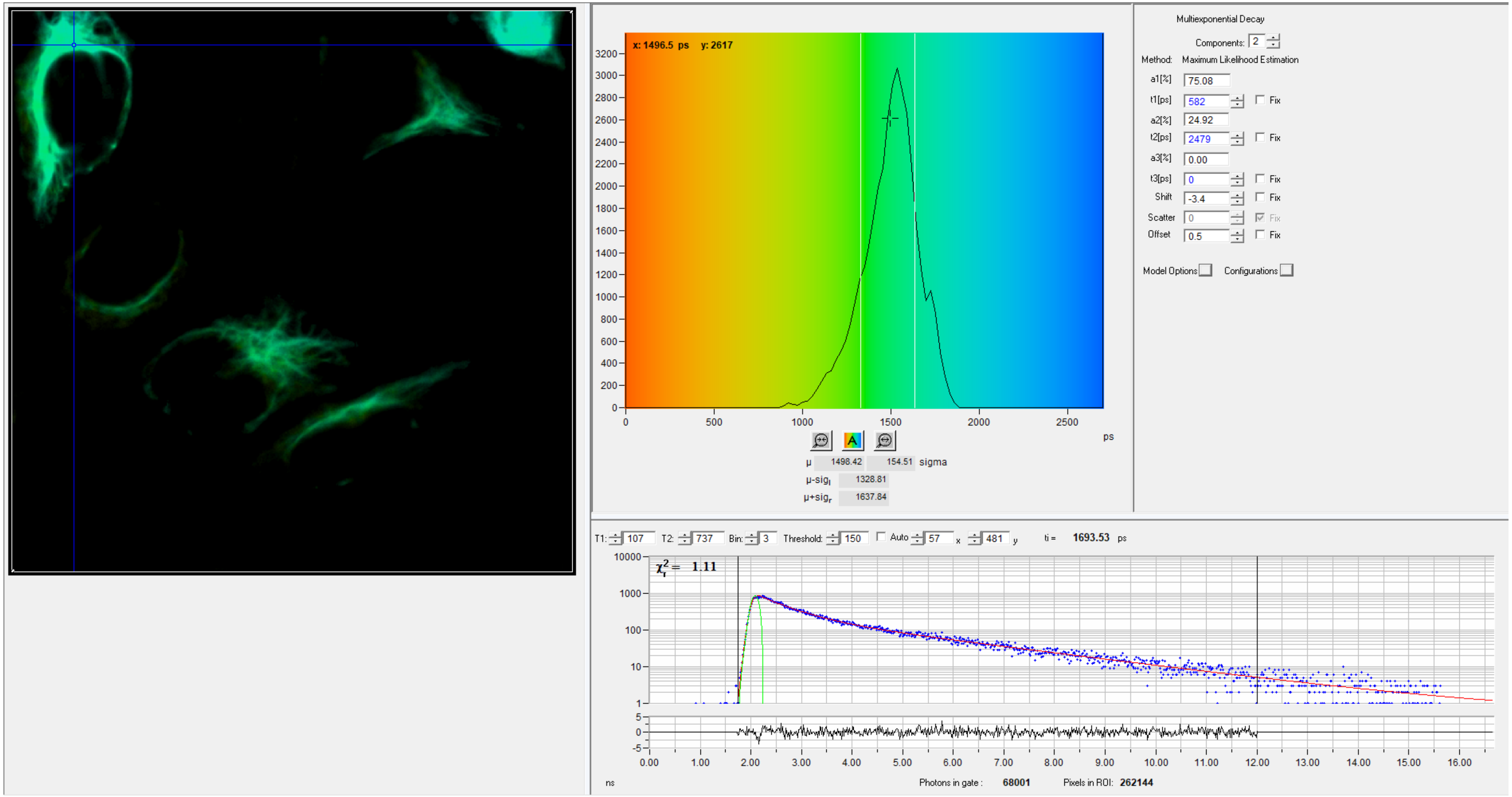

Figure S43. **Vimentin-R52Y** fuse in complex with **N871b**; bi-exponential fit; τ_i color-coding. FLIM scan and corresponding time-resolved fluorescence data analysis of life HeLa cells expressing the R52Y FAST variant fused to vimentin and stained with **N871b**. A screenshot from Becker&Hickl SPCImage data acquisition and analysis window is shown. Bi-exponential fitting of decay data was performed. On the left panel is a FLIM image of HeLa nuclei, color-coded according to intensity-weighted average fluorescence lifetime in each pixel (τ_i). A histogram on the upper right panel displays distribution of τ_i and color legend. The right panel represents bi-exponential fitting model used to fit data and fitting results. The lower data shows data on fluorescence decay. Blue dots represent experimental decay data, red line represents bi-exponential fit, green line represents instrument response function (IRF), fitting residuals shown as black graph below main data plot.

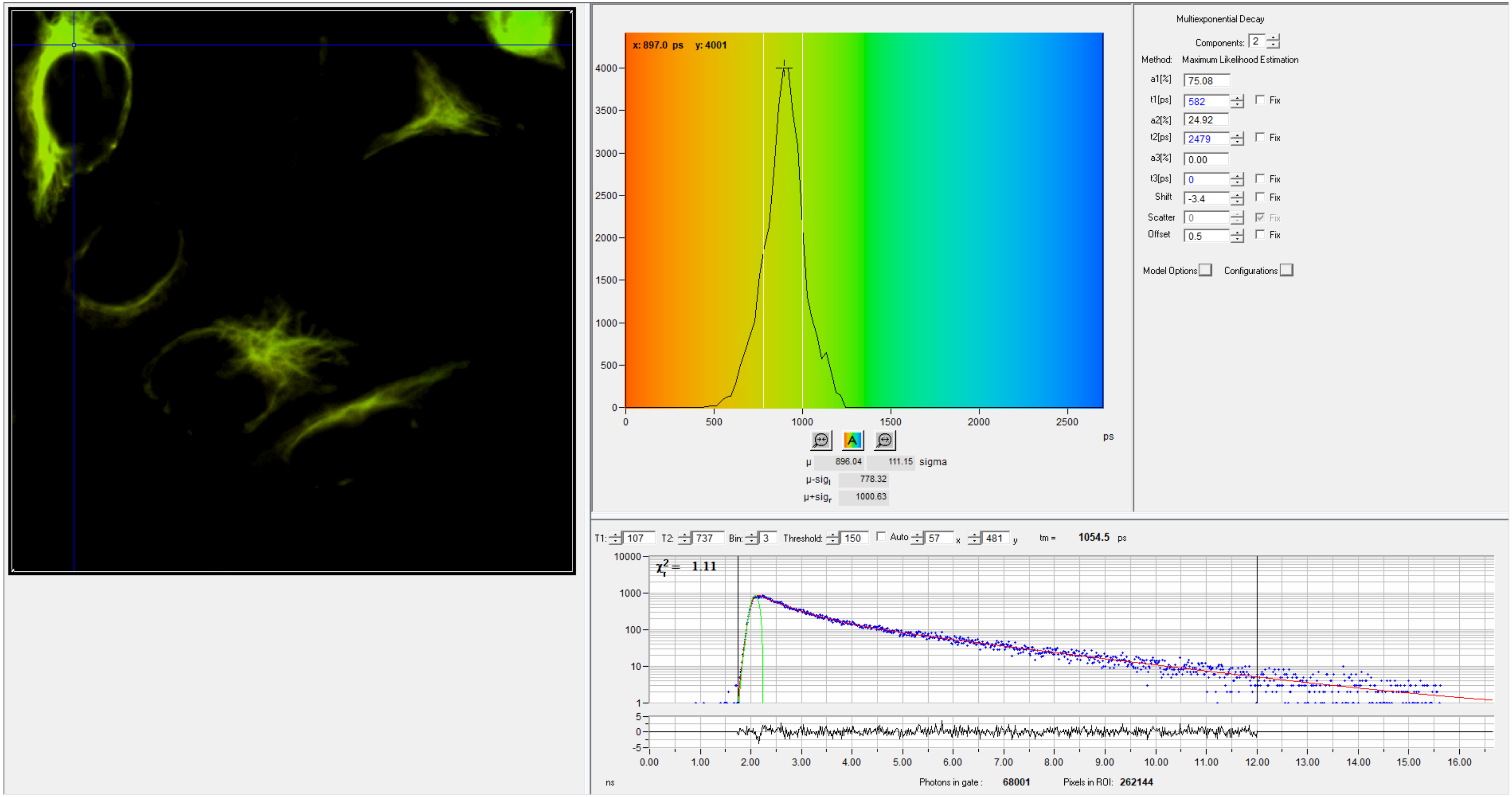

Figure S44. **Vimentin-R52Y** fuse in complex with **N871b**; bi-exponential fit; τ_m color-coding. FLIM scan and corresponding time-resolved fluorescence data analysis of life HeLa cells expressing the R52Y FAST variant fused to vimentin and stained with **N871b**. A screenshot from Becker&Hickl SPCImage data acquisition and analysis window is shown. Bi-exponential fitting of decay data was performed. On the left panel is a FLIM image of HeLa nuclei, color-coded according to amplitude-weighted average fluorescence lifetime in each pixel (τ_m). A histogram on the upper right panel displays distribution of τ_m and color legend. The right panel represents bi-exponential fitting model used to fit data and fitting results. The lower data shows data on fluorescence decay. Blue dots represent experimental decay data, red line represents bi-exponential fit, green line represents instrument response function (IRF), fitting residuals shown as black graph below main data plot.

Table S4. Comparison of average fluorescence lifetimes (τ_i and τ_m) of **N871b** fluorogen in complexes with FAST variants expressed in HeLa Kyoto cells as fuses with H2B, IMS and vimentin. Bi-exponential fitting model. *SD* is standard deviation; *n* is a number of individual cells taken for analysis.

FAST variant	τ_i ± SD, ns			τ_m ± SD, ns		
	H2B	IMS	Vimentin	H2B	IMS	Vimentin
**D65K**	1.342 ± 0.059<br>n = 30	1.254 ± 0.067<br>n = 30	1.295 ± 0.043<br>n = 18	0.916 ± 0.036<br>n = 30	0.794 ± 0.075<br>n = 30	0.903 ± 0.032<br>n = 18
**F62L**	1.009 ± 0.054<br>n = 30	1.060 ± 0.170<br>n = 30	0.897 ± 0.046<br>n = 22	0.663 ± 0.026<br>n = 30	0.659 ± 0.026<br>n = 30	0.617 ± 0.025<br>n = 22
**P68K**	1.114 ± 0.079<br>n = 30	0.978 ± 0.032<br>n = 17	1.027 ± 0.051<br>n = 22	0.765 ± 0.040<br>n = 30	0.643 ± 0.027<br>n = 17	0.689 ± 0.077<br>n = 22
**R52Y**	1.926 ± 0.092<br>n = 30	1.512 ± 0.072<br>n = 23	1.591 ± 0.070<br>n = 20	1.251 ± 0.099<br>n = 30	0.917 ± 0.069<br>n = 23	0.974 ± 0.067<br>n = 20

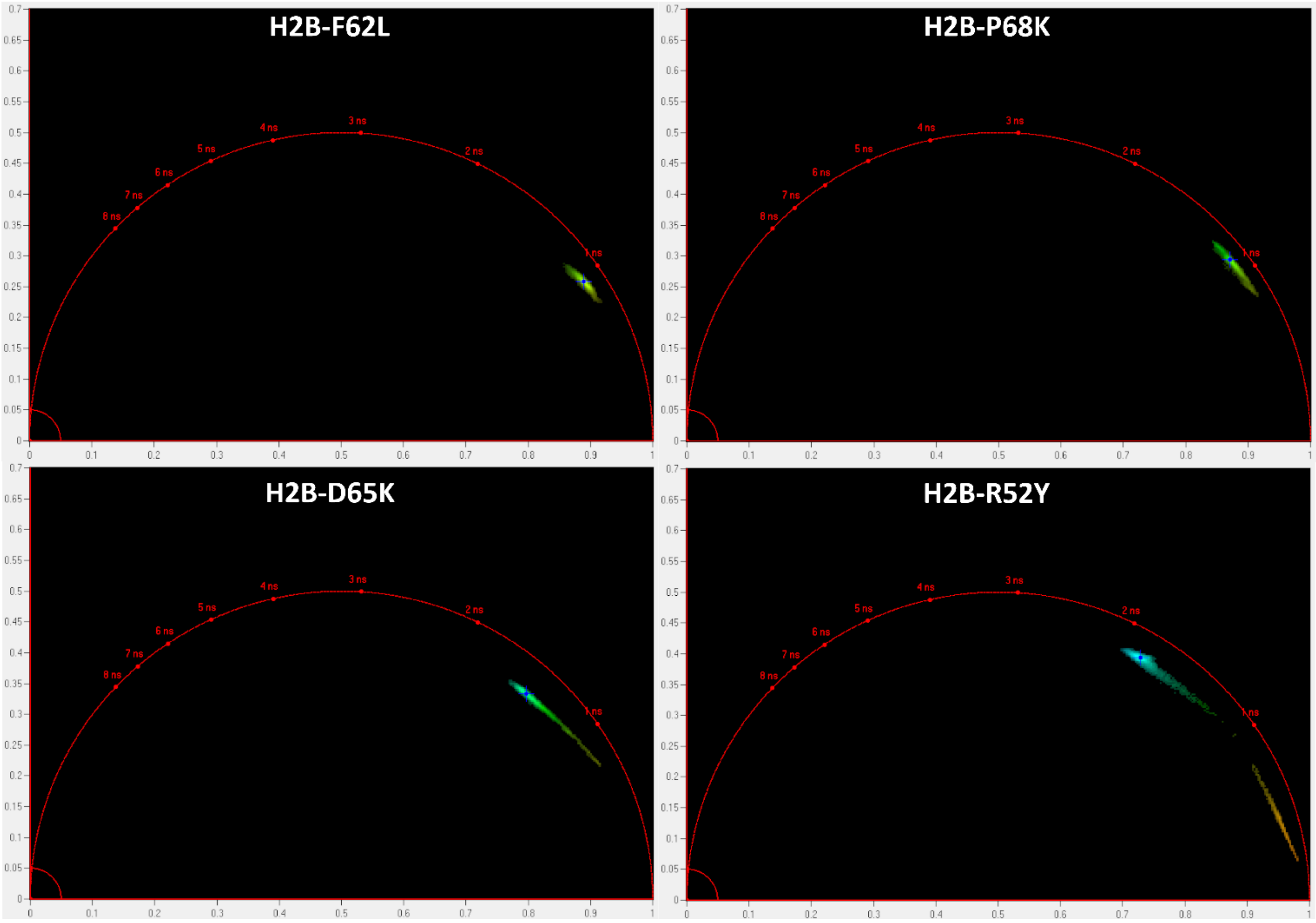

Figure S45. Phasor plots of selected FAST variants fuses with H2B in HeLa Kyoto cells, τ_i color-coding

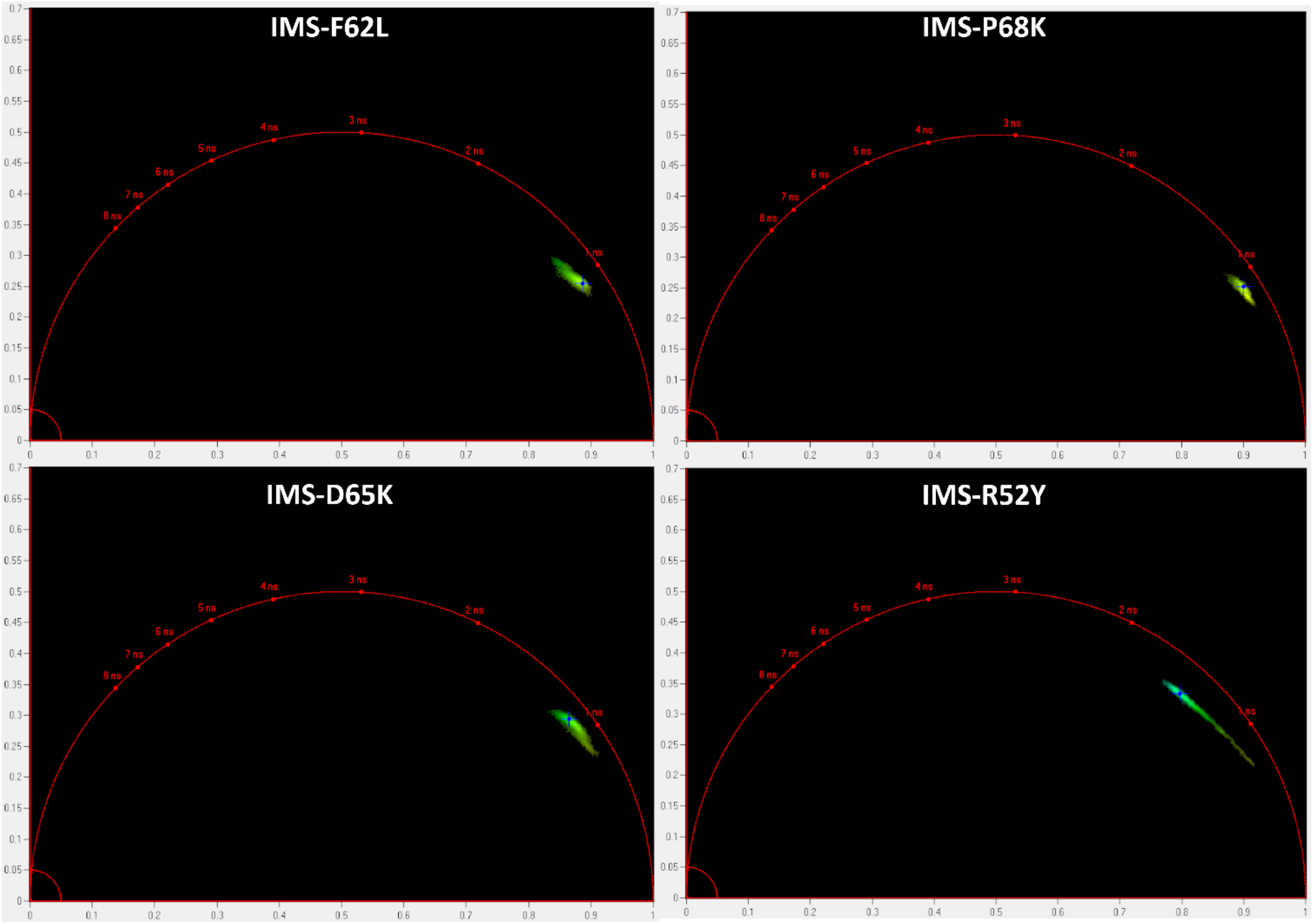

Figure S46. Phasor plots of selected FAST variants fuses with IMS in HeLa Kyoto cells, τ_i color-coding

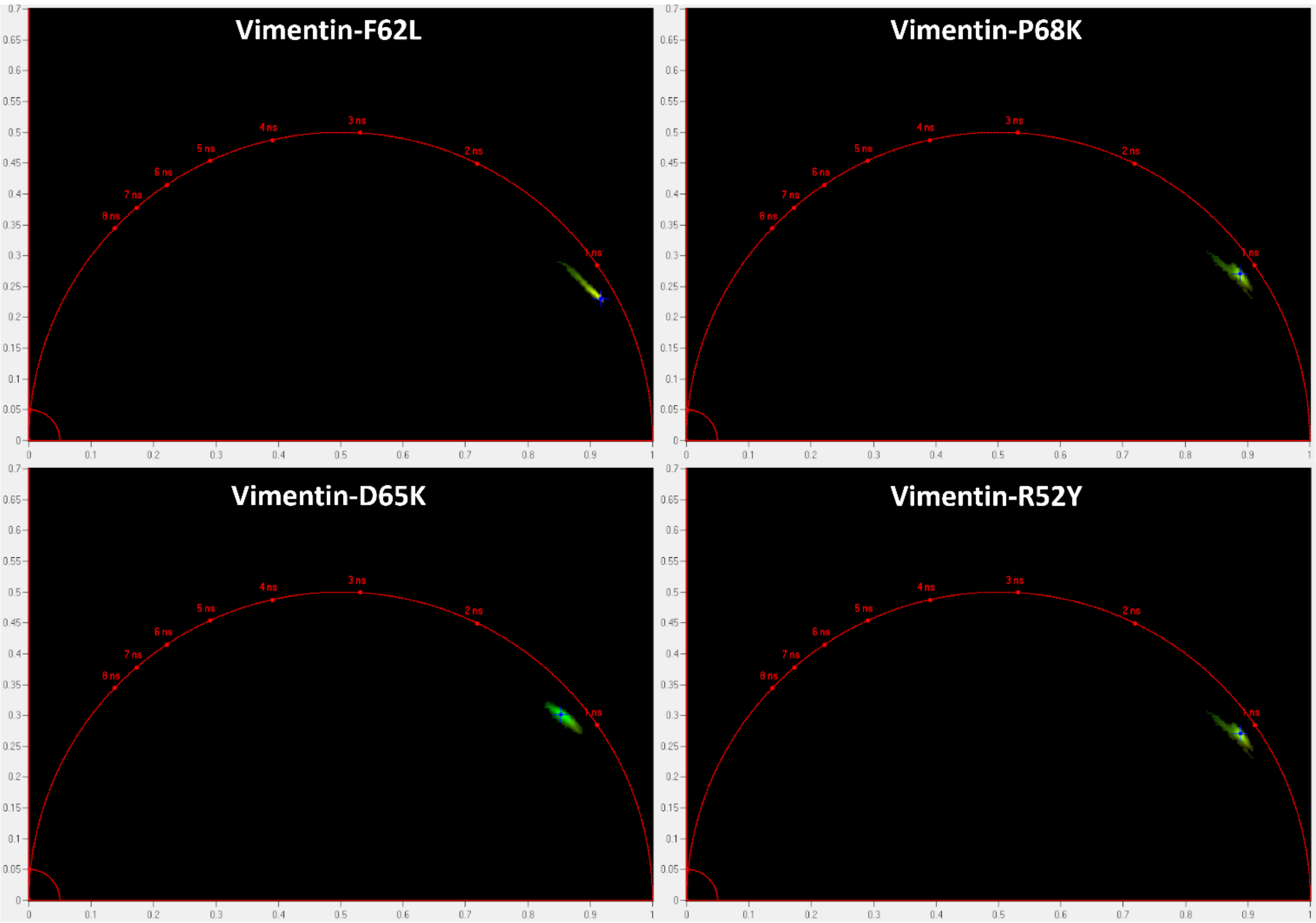

Figure S47. Phasor plots of selected FAST variants fuses with vimentin in HeLa Kyoto cells, τ_i color-coding

4 FLIM-based visualization of multiple intracellular targets

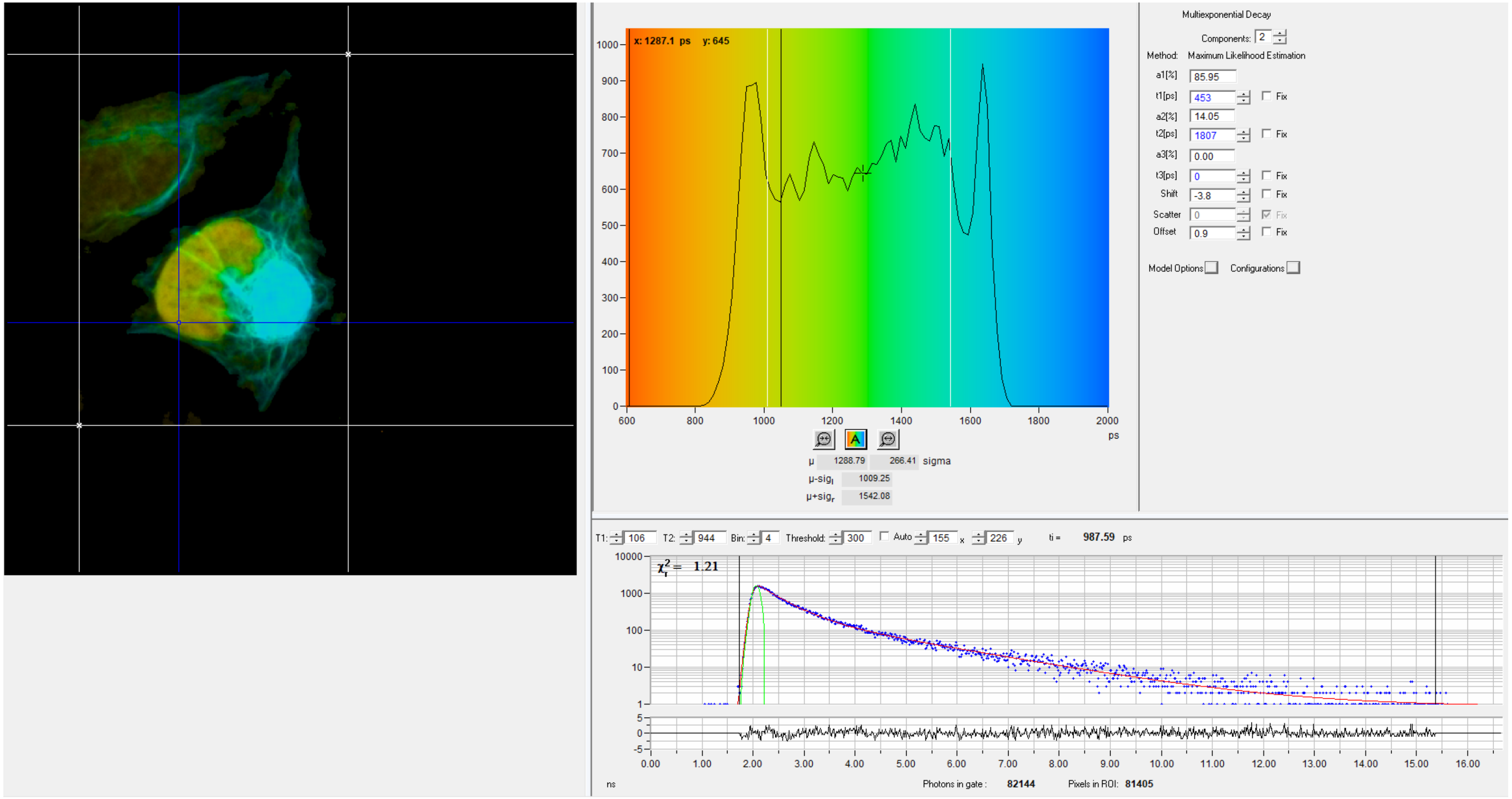

Figure S48. H2B-F62L and vimentin-R52Y fuses in complex with **N871b**; bi-exponential fit; τ_i color-coding. FLIM scan and corresponding time-resolved fluorescence data analysis of life HeLa cells expressing F62L and R52Y variants fused to H2B and vimentin respectively, and stained with **N871b**. A screenshot from Becker&Hickl SPCImage data acquisition and analysis window is shown. Bi-exponential fitting of decay data was performed. On the left panel is a FLIM image of HeLa nuclei and vimentin, color-coded according to intensity-weighted average fluorescence lifetime in each pixel (τ_i). A histogram on the upper right panel displays distribution of τ_i and color legend. The right panel represents bi-exponential model used to fit data and fitting results. The lower data represents bi-exponential fit, red line represents bi-exponential fit, green line represents instrument response function (IRF), fitting residuals shown as black graph below main data plot.

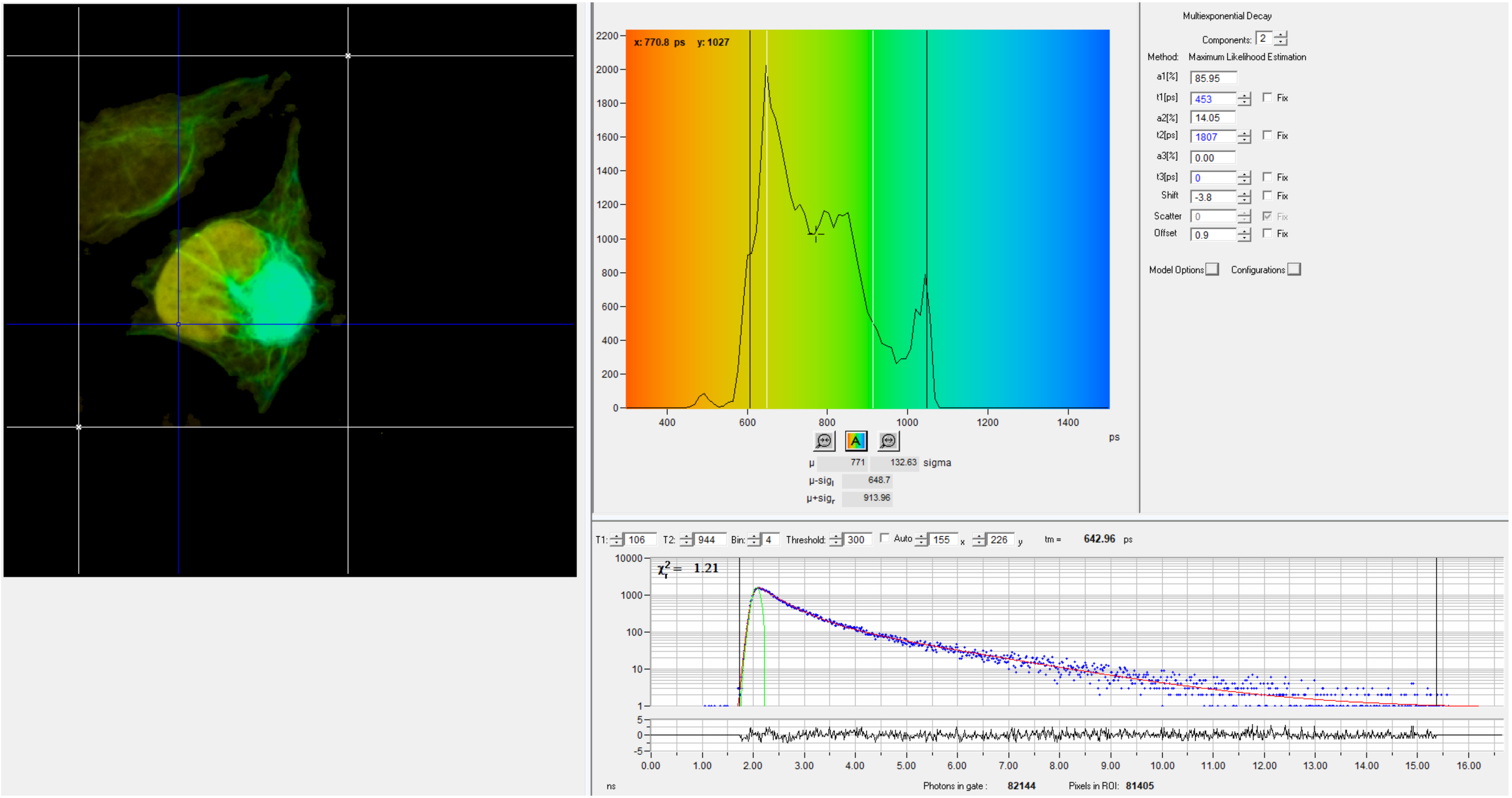

Figure S49. H2B-F62L and vimentin-R52Y fuses in complex with **N871b**; bi-exponential fit; τ_m color-coding. FLIM scan and corresponding time-resolved fluorescence data analysis of life HeLa cells expressing F62L and R52Y variants fused to H2B and vimentin respectively, and stained with **N871b**. A screenshot from Becker&Hickl SPCImage data acquisition and analysis window is shown. Bi-exponential fitting of decay data was performed. On the left panel is a FLIM image of HeLa nuclei and vimentin, color-coded according to amplitude-weighted average fluorescence lifetime in each pixel (τ_m). A histogram on the upper right panel displays distribution of τ_m and color legend. The right panel represents bi-exponential model used to fit data and fitting results. The lower data represents bi-exponential fit, red line represents bi-exponential fit, green line represents instrument response function (IRF), fitting residuals shown as black graph below main data plot.

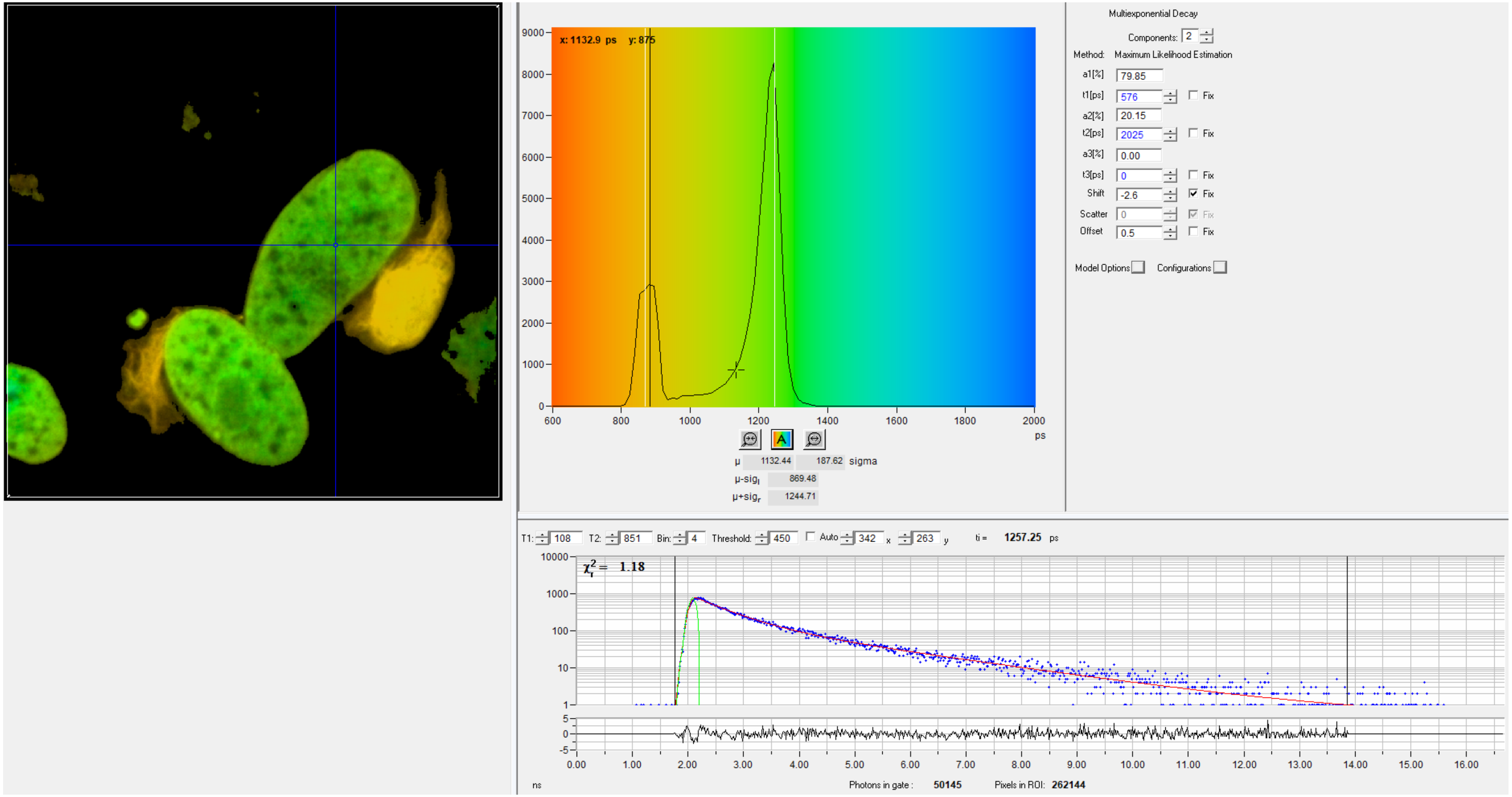

Figure S50. H2B-D65K and vimentin-F62L fuses in complex with **N871b**; bi-exponential fit; τ_i color-coding. FLIM scan and corresponding time-resolved fluorescence data analysis of life HeLa cells expressing D65K and F62L variants fused to H2B and vimentin respectively, and stained with **N871b**. A screenshot from Becker&Hickl SPCImage data acquisition and analysis window is shown. Bi-exponential fitting of decay data was performed. On the left panel is a FLIM image of HeLa nuclei and vimentin, color-coded according to intensity-weighted average fluorescence lifetime in each pixel (τ_i). A histogram on the upper right panel displays distribution of τ_i and color legend. The right panel represents bi-exponential model used to fit data and fitting results. The lower data represents bi-exponential fit, red line represents bi-exponential fit, green line represents instrument response function (IRF), fitting residuals shown as black graph below main data plot.

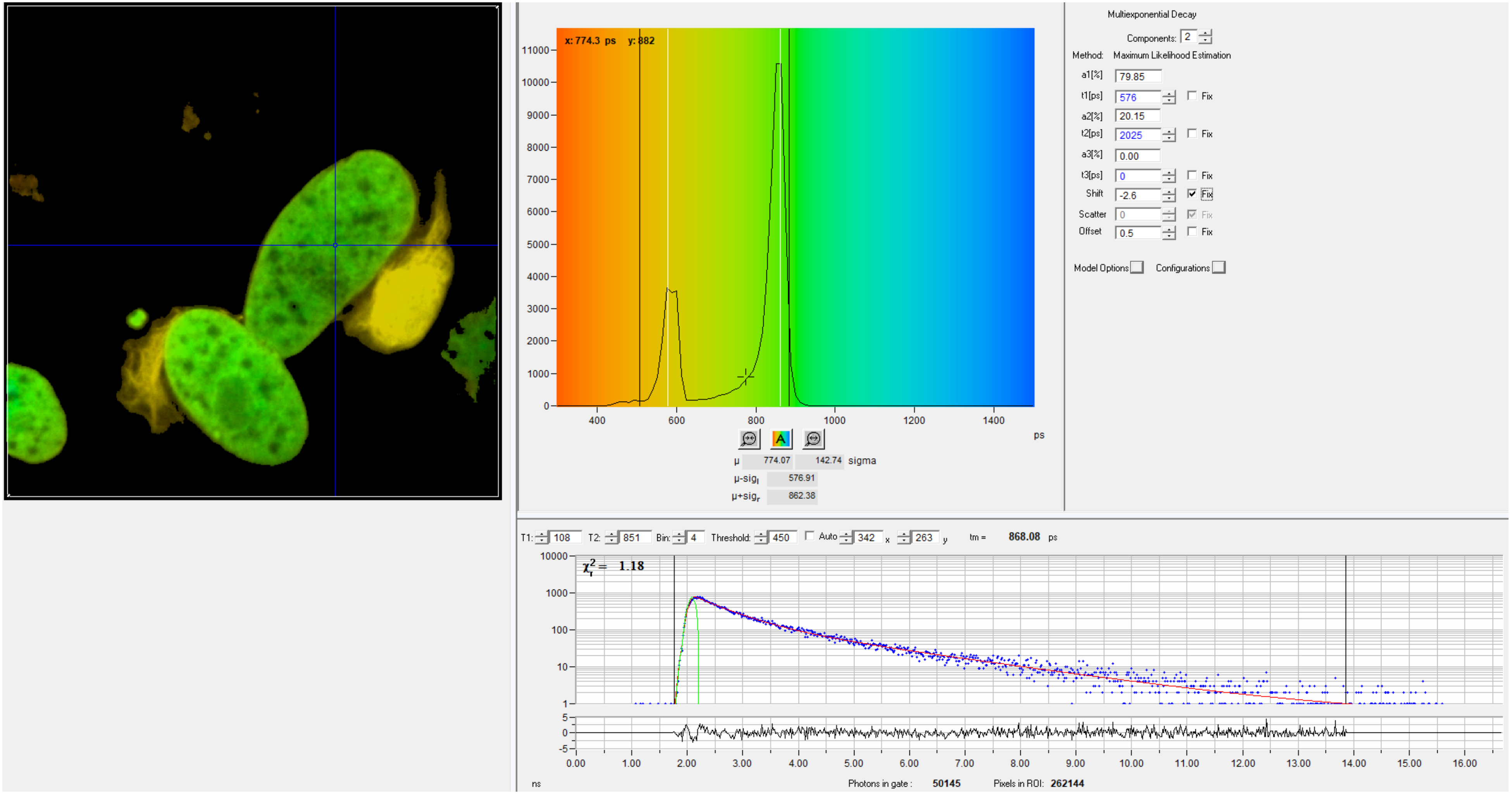

Figure S51. H2B-D65K and vimentin-F62L fuses in complex with **N871b**; bi-exponential fit; τ_m color-coding. FLIM scan and corresponding time-resolved fluorescence data analysis of life HeLa cells expressing D65K and F62L variants fused to H2B and vimentin respectively, and stained with **N871b**. A screenshot from Becker&Hickl SPCImage data acquisition and analysis window is shown. Bi-exponential fitting of decay data was performed. On the left panel is a FLIM image of HeLa nuclei and vimentin, color-coded according to amplitude-weighted average fluorescence lifetime in each pixel (τ_m). A histogram on the upper right panel displays distribution of τ_m and color legend. The right panel represents bi-exponential model used to fit data and fitting results. The lower data represents bi-exponential fit, red line represents bi-exponential fit, green line represents instrument response function (IRF), fitting residuals shown as black graph below main data plot.

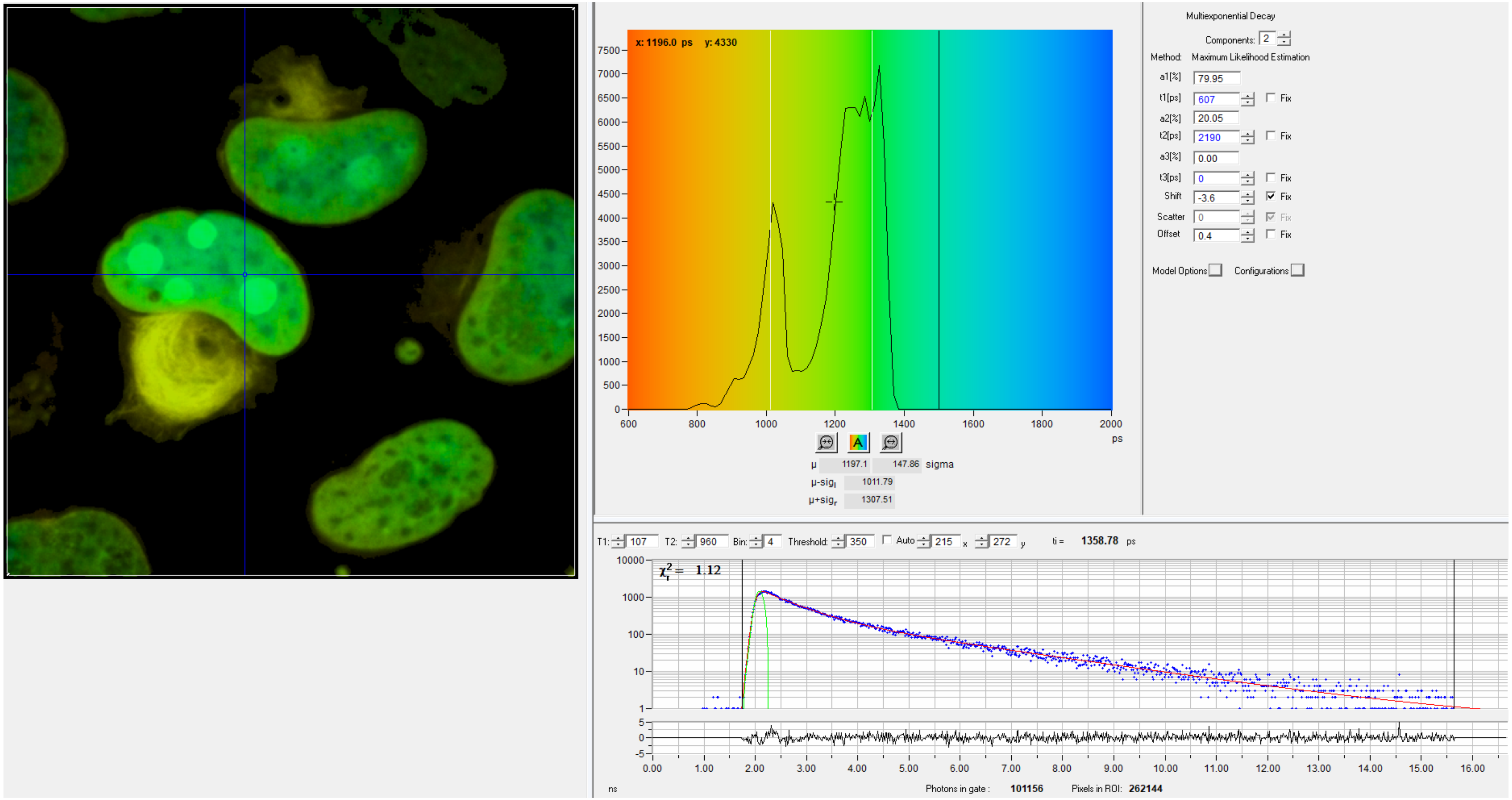

Figure S52. H2B-D65K and vimentin-P68K fuses in complex with **N871b**; bi-exponential fit; τ_i color-coding. FLIM scan and corresponding time-resolved fluorescence data analysis of life HeLa cells expressing D65K and P68K variants fused to H2B and vimentin respectively, and stained with **N871b**. A screenshot from Becker&Hickl SPCImage data acquisition and analysis window is shown. Bi-exponential fitting of decay data was performed. On the left panel is a FLIM image of HeLa nuclei and vimentin, color-coded according to intensity-weighted average fluorescence lifetime in each pixel (τ_i). A histogram on the upper right panel displays distribution of τ_i and color legend. The right panel represents bi-exponential model used to fit data and fitting results. The lower data represents bi-exponential fit, red line represents bi-exponential fit, green line represents instrument response function (IRF), fitting residuals shown as black graph below main data plot.

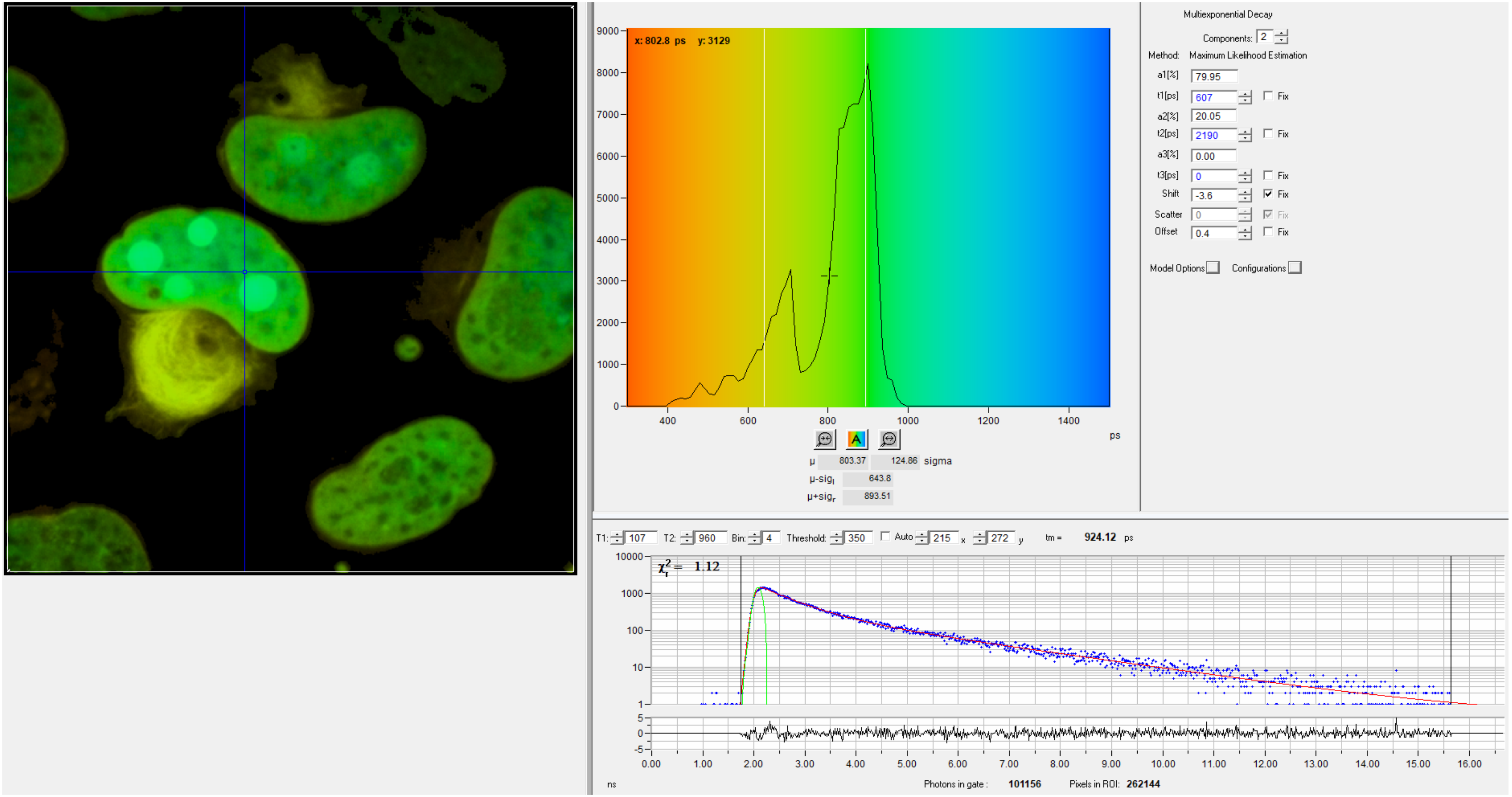

Figure S53. H2B-D65K and vimentin-P68K fuses in complex with **N871b**; bi-exponential fit; τ_m color-coding. FLIM scan and corresponding time-resolved fluorescence data analysis of life HeLa cells expressing D65K and P68K variants fused to H2B and vimentin respectively, and stained with **N871b**. A screenshot from Becker&Hickl SPCImage data acquisition and analysis window is shown. Bi-exponential fitting of decay data was performed. On the left panel is a FLIM image of HeLa nuclei and vimentin, color-coded according to amplitude-weighted average fluorescence lifetime in each pixel (τ_m). A histogram on the upper right panel displays distribution of τ_m and color legend. The right panel represents bi-exponential model used to fit data and fitting results. The lower data represents bi-exponential fit, red line represents bi-exponential fit, green line represents instrument response function (IRF), fitting residuals shown as black graph below main data plot.

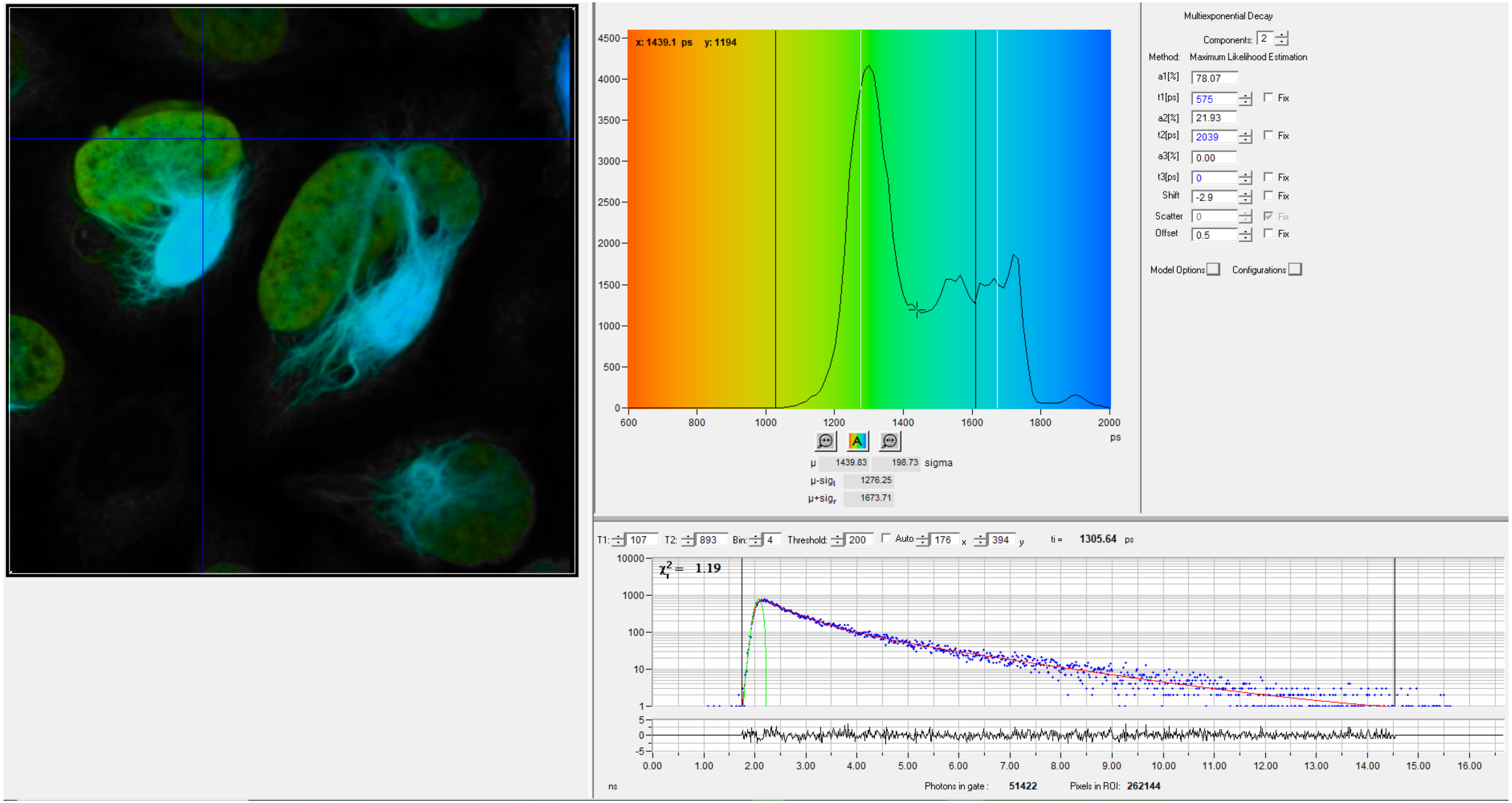

Figure S54. H2B-D65K and vimentin-R52Y fuses in complex with **N871b**; bi-exponential fit; τ_i color-coding. FLIM scan and corresponding time-resolved fluorescence data analysis of life HeLa cells expressing D65K and R52Y variants fused to H2B and vimentin respectively, and stained with **N871b**. A screenshot from Becker&Hickl SPCImage data acquisition and analysis window is shown. Bi-exponential fitting of decay data was performed. On the left panel is a FLIM image of HeLa nuclei and vimentin, color-coded according to intensity-weighted average fluorescence lifetime in each pixel (τ_i). A histogram on the upper right panel displays distribution of τ_i and color legend. The right panel represents bi-exponential model used to fit data and fitting results. The lower data represents bi-exponential fit, red line represents bi-exponential fit, green line represents instrument response function (IRF), fitting residuals shown as black graph below main data plot.

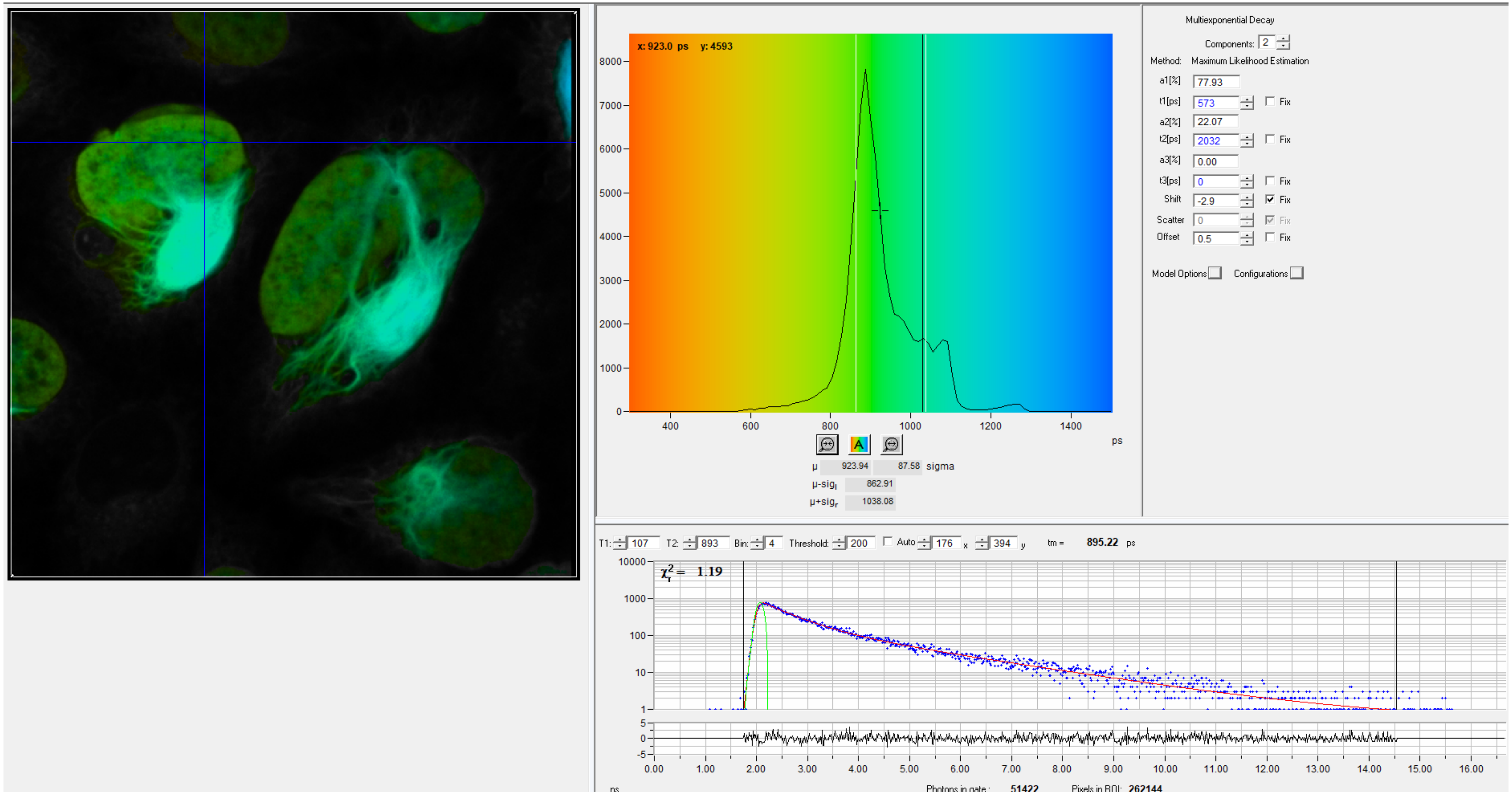

Figure S55. H2B-D65K and vimentin-R52Y fuses in complex with **N871b**; bi-exponential fit; τ_m color-coding. FLIM scan and corresponding time-resolved fluorescence data analysis of life HeLa cells expressing D65K and R52Y variants fused to H2B and vimentin respectively, and stained with **N871b**. A screenshot from Becker&Hickl SPCImage data acquisition and analysis window is shown. Bi-exponential fitting of decay data was performed. On the left panel is a FLIM image of HeLa nuclei and vimentin, color-coded according to amplitude-weighted average fluorescence lifetime in each pixel (τ_m). A histogram on the upper right panel displays distribution of τ_m and color legend. The right panel represents bi-exponential model used to fit data and fitting results. The lower data represents bi-exponential fit, red line represents bi-exponential fit, green line represents instrument response function (IRF), fitting residuals shown as black graph below main data plot.

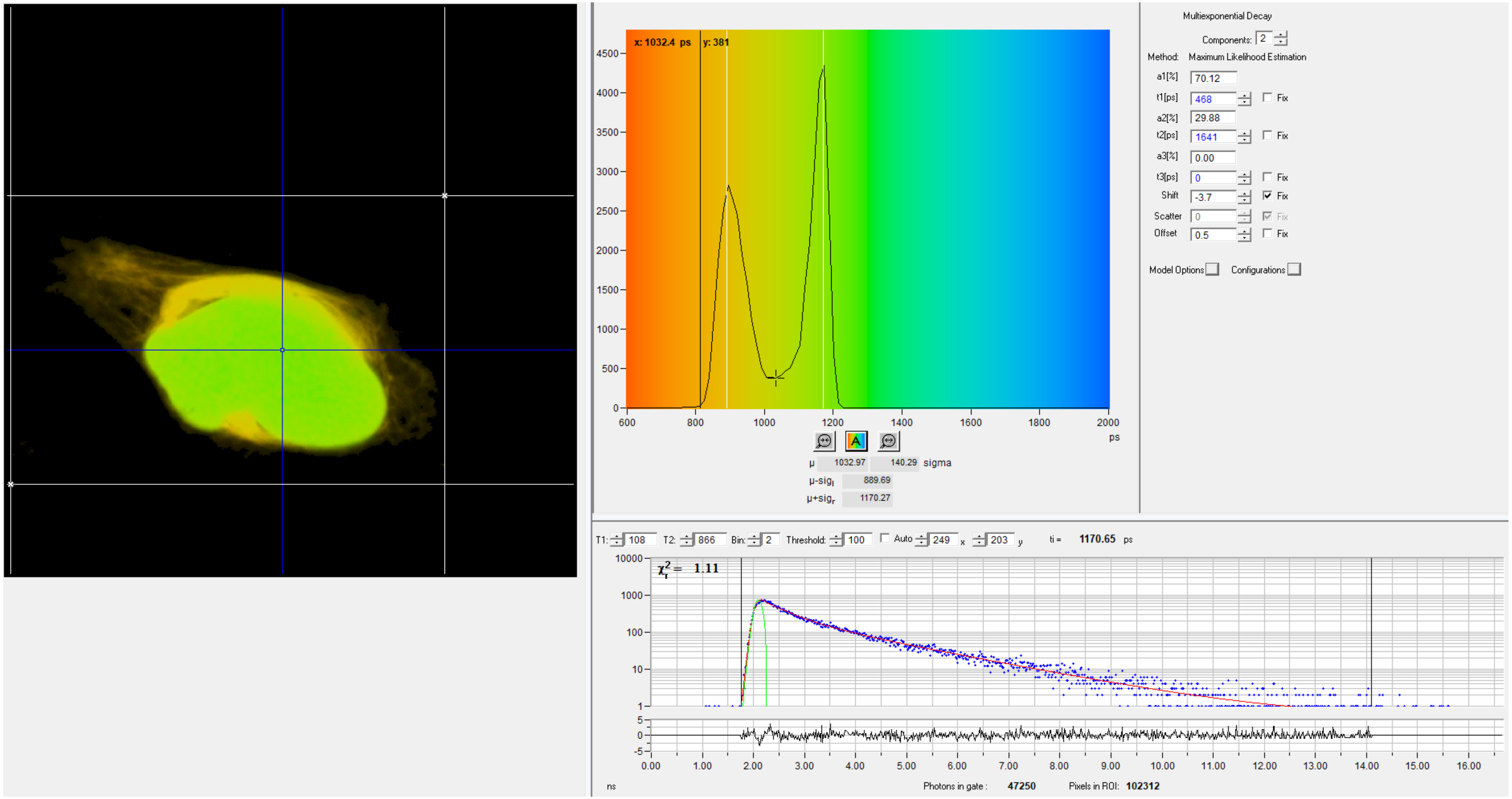

Figure S56. H2B-P68K and vimentin-F62L fuses in complex with **N871b**; bi-exponential fit; τ_i color-coding. FLIM scan and corresponding time-resolved fluorescence data analysis of life HeLa cells expressing P68K and F62L variants fused to H2B and vimentin respectively, and stained with **N871b**. A screenshot from Becker&Hickl SPCImage data acquisition and analysis window is shown. Bi-exponential fitting of decay data was performed. On the left panel is a FLIM image of HeLa nuclei and vimentin, color-coded according to intensity-weighted average fluorescence lifetime in each pixel (τ_i). A histogram on the upper right panel displays distribution of τ_i and color legend. The right panel represents bi-exponential model used to fit data and fitting results. The lower data represents bi-exponential fit, red line represents bi-exponential fit, green line represents instrument response function (IRF), fitting residuals shown as black graph below main data plot.

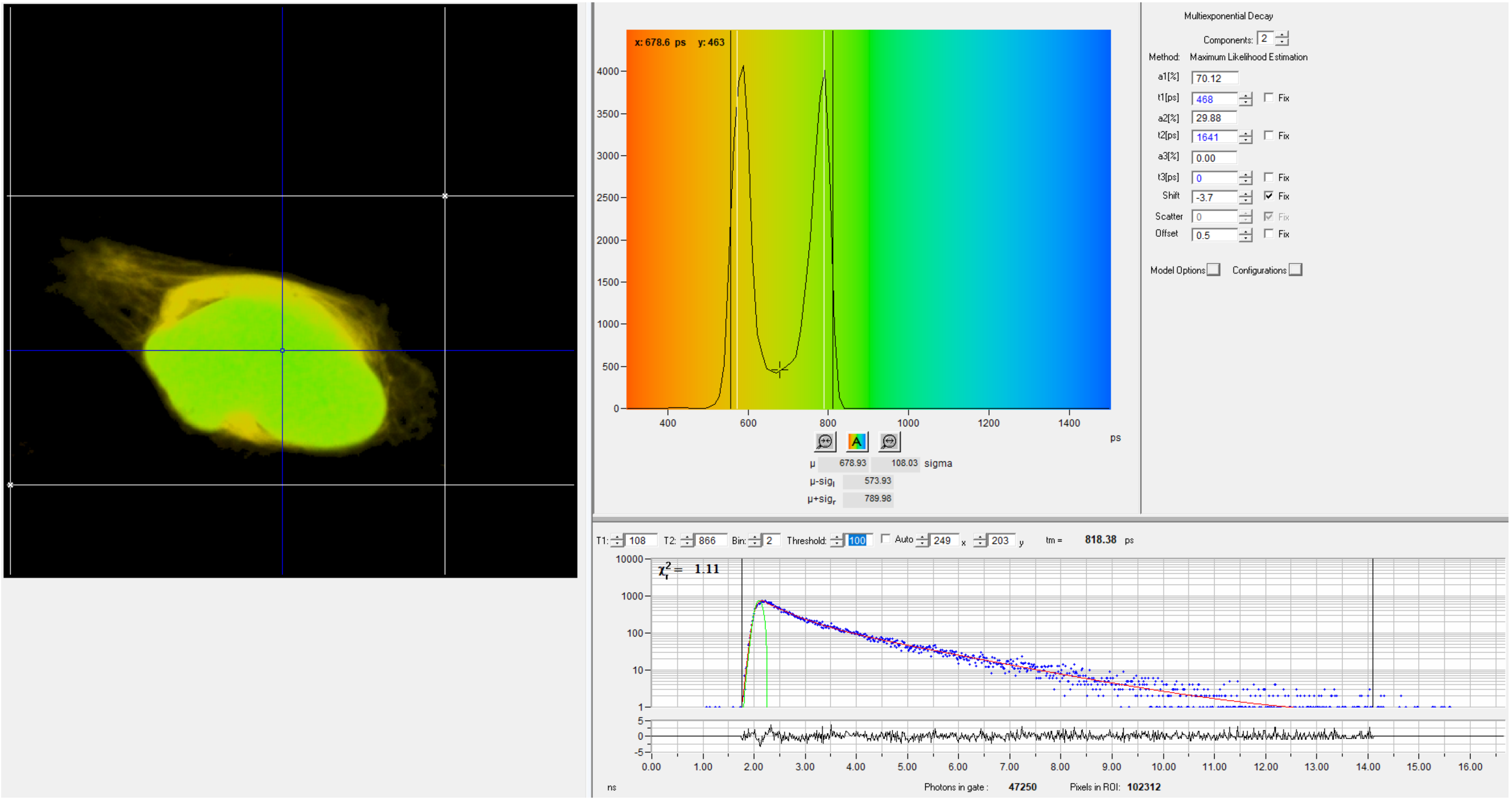

Figure S57. H2B-P68K and vimentin-F62L fuses in complex with **N871b**; bi-exponential fit; τ_m color-coding. FLIM scan and corresponding time-resolved fluorescence data analysis of life HeLa cells expressing P68K and F62L variants fused to H2B and vimentin respectively, and stained with **N871b**. A screenshot from Becker&Hickl SPCImage data acquisition and analysis window is shown. Bi-exponential fitting of decay data was performed. On the left panel is a FLIM image of HeLa nuclei and vimentin, color-coded according to amplitude-weighted average fluorescence lifetime in each pixel (τ_m). A histogram on the upper right panel displays distribution of τ_m and color legend. The right panel represents bi-exponential model used to fit data and fitting results. The lower data represents bi-exponential fit, red line represents bi-exponential fit, green line represents instrument response function (IRF), fitting residuals shown as black graph below main data plot.

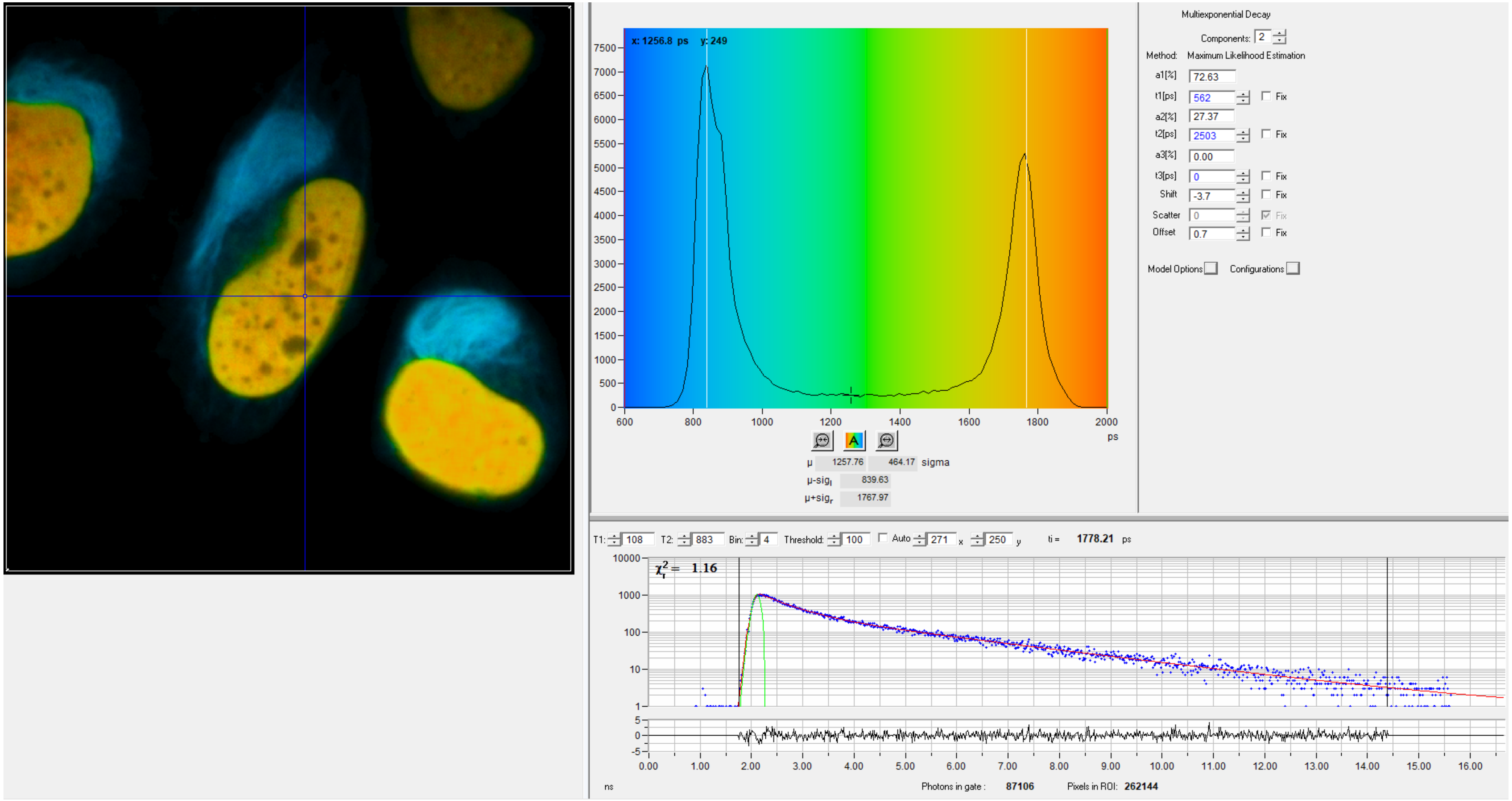

Figure S58. H2B-R52Y and vimentin-F62L fuses in complex with **N871b**; bi-exponential fit; τ_i color-coding. FLIM scan and corresponding time-resolved fluorescence data analysis of life HeLa cells expressing R52Y and F62L variants fused to H2B and vimentin respectively, and stained with **N871b**. A screenshot from Becker&Hickl SPCImage data acquisition and analysis window is shown. Bi-exponential fitting of decay data was performed. On the left panel is a FLIM image of HeLa nuclei and vimentin, color-coded according to intensity-weighted average fluorescence lifetime in each pixel (τ_i). A histogram on the upper right panel displays distribution of τ_i and color legend. The right panel represents bi-exponential model used to fit data and fitting results. The lower data represents bi-exponential fit, red line represents bi-exponential fit, green line represents instrument response function (IRF), fitting residuals shown as black graph below main data plot.

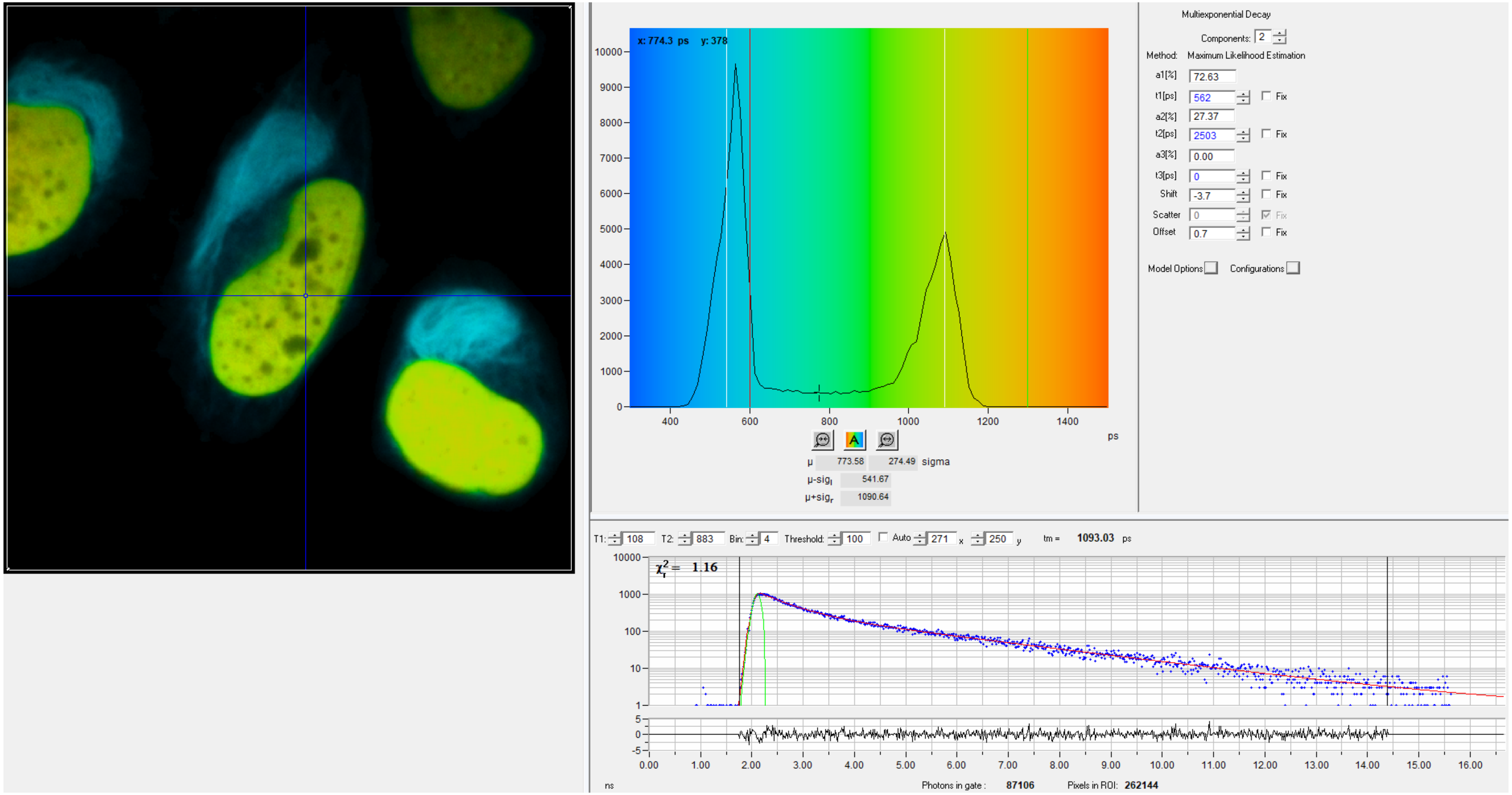

Figure S59. H2B-R52Y and vimentin-F62L fuses in complex with **N871b**; bi-exponential fit; τ_m color-coding. FLIM scan and corresponding time-resolved fluorescence data analysis of life HeLa cells expressing R52Y and F62L variants fused to H2B and vimentin respectively, and stained with **N871b**. A screenshot from Becker&Hickl SPCImage data acquisition and analysis window is shown. Bi-exponential fitting of decay data was performed. On the left panel is a FLIM image of HeLa nuclei and vimentin, color-coded according to amplitude-weighted average fluorescence lifetime in each pixel (τ_m). A histogram on the upper right panel displays distribution of τ_m and color legend. The right panel represents bi-exponential model used to fit data and fitting results. The lower data represents bi-exponential fit, red line represents bi-exponential fit, green line represents instrument response function (IRF), fitting residuals shown as black graph below main data plot.

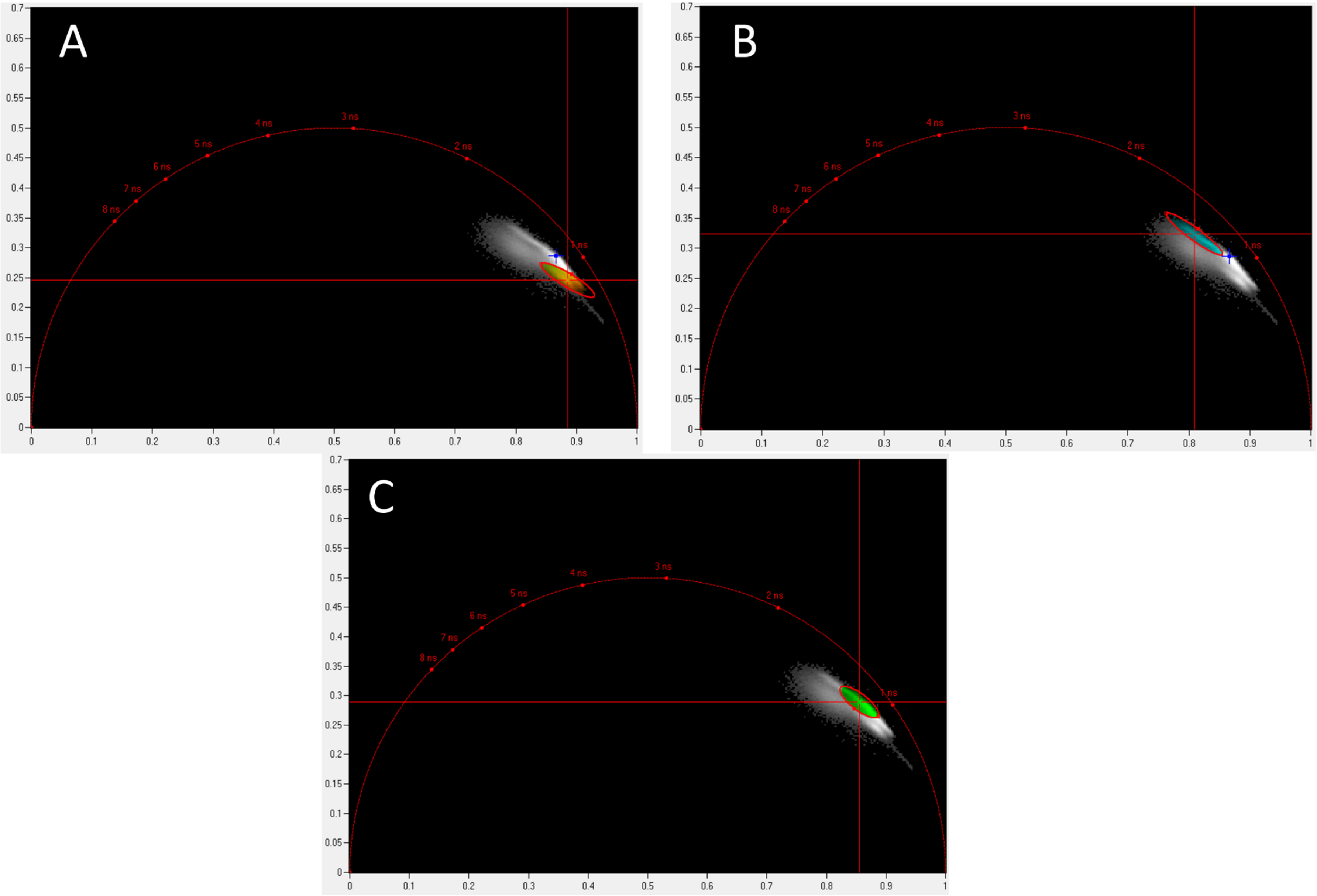

Figure S60. Phasor plots for figure 3. A - IMS-F62L cluster, B - H2B-R52Y cluster, C - vimentin-P68K cluster

5 Compound N871b description

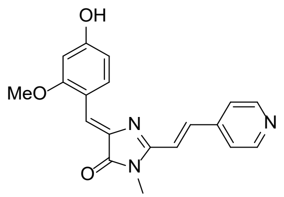

5-((Z)-4-hydroxy-2-methoxybenzylidene)-3-methyl-2-((E)-2-(pyridin-4-yl)vinyl)-3,5-dihydro-4H-imidazol-4-one (N871b)

Dark red solid; mp = 263–265 ºC; ^{1}H NMR (300 MHz, 303 K, DMSO-d_6) δ ppm 8.83 (d, *J*=8.6 Hz, 1H), 8.65 (d, *J*=5.9 Hz, 2H), 7.89 (d, *J*=15.8 Hz, 1H), 7.80 (d, *J*=5.9 Hz, 2H), 7.47 (d, *J*=15.8 Hz, 1H), 7.40 (s, 1H), 6.55 (dd, *J*=8.7, 2.2 Hz, 1H), 6.47 (d, *J*=2.2 Hz, 1H), 3.86 (s, 3H), 3.28 (s, 3H); ^{13}C NMR (75 MHz, 303 K, DMSO-d_6) δ ppm 169.9, 162.1, 160.9, 157.5, 150.2, 142.3, 136.2, 135.9, 134.4, 121.9, 120.5, 118.9, 114.5, 108.8, 98.6, 55.7, 26.5; HRMS (ESI) m/z: 336.1337 found (calcd for $C_{19}H_{18}N_3O_3^+$, $[M+H]^+$ 336.1343).

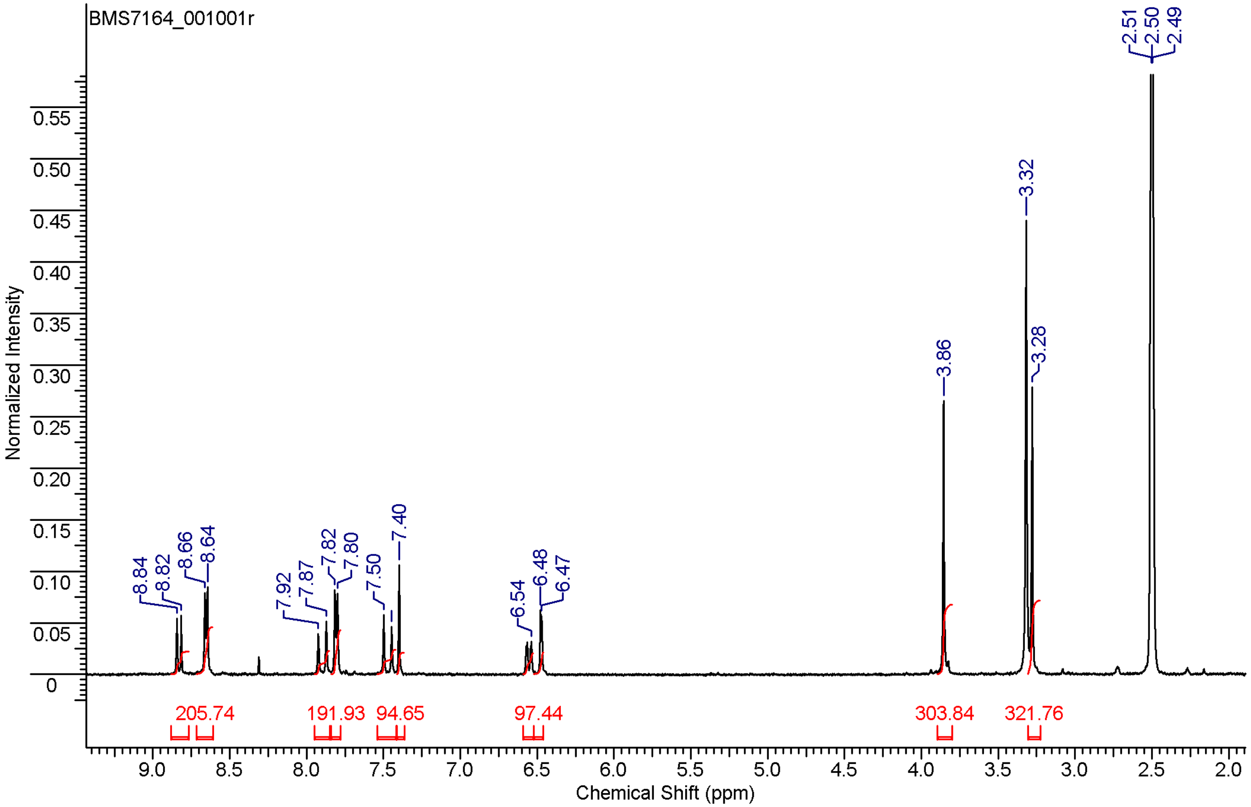

Figure S61. ^{1}H NMR spectrum of compound **N871b**.

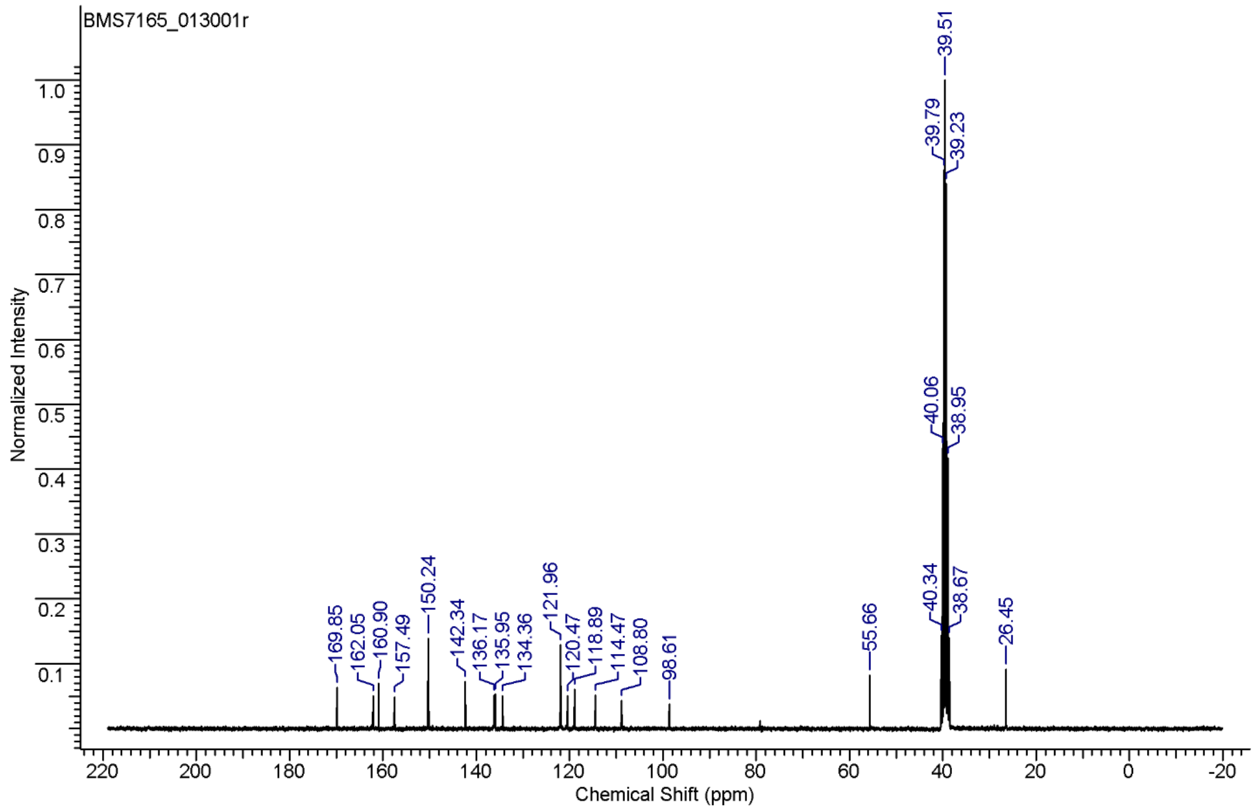

Figure S62. ^{13}C NMR spectrum of compound **N871b**.

6 References

1. Goncharuk, M. V.; Baleeva, N. S.; Nolde, D. E.; Gavrikov, A. S.; Mishin, A. V.; Mishin, A. S.; Sosorev, A. Y.; Arseniev, A. S.; Goncharuk, S. A.; Borshchevskiy, V. I.; Efremov, R. G.; Mineev, K. S.; Baranov, M. S. Structure-Based Rational Design of an Enhanced Fluorogen-Activating Protein for Fluorogens Based on GFP Chromophore. *Commun. Biol.* **2022**, *5* (1), 706. https://doi.org/10.1038/s42003-022-03662-9.
2. Bogdanova, Y. A.; Solovyev, I. D.; Baleeva, N. S.; Myasnyanko, I. N.; Gorshkova, A. A.; Gorbachev, D. A.; Gilvanov, A. R.; Goncharuk, S. A.; Goncharuk, M. V.; Mineev, K. S.; Arseniev, A. S.; Bogdanov, A. M.; Savitsky, A. P.; Baranov, M. S. Fluorescence Lifetime Multiplexing with Fluorogen Activating Protein FAST Variants. *Commun. Biol.* **2024**, 7 (1), 799. https://doi.org/10.1038/s42003-024-06501-1.
